# Single‐Nucleus Transcriptome Reveals Cellular Heterogeneity and Transcriptional Response to Heat Stress in Skeletal Muscle

**DOI:** 10.1002/jcsm.70217

**Published:** 2026-02-12

**Authors:** Ziyin Han, Li Chen, Zhiyu Lei, Jiaman Zhang, Ziyu Chen, Xiaolan Fan, Bo Zeng, Anan Jiang, Hai Xiang, Hua Li, Mingzhou Li, Long Jin

**Affiliations:** ^1^ State Key Laboratory of Swine and Poultry Breeding Industry, College of Animal Science and Technology Sichuan Agricultural University Chengdu China; ^2^ Guangdong Provincial Key Laboratory of Animal Molecular Design and Precise Breeding Foshan University Foshan China; ^3^ Chongqing Academy of Animal Science Chongqing China; ^4^ Key Laboratory of Animal Resource Evaluation and Utilization (Pigs) Ministry of Agriculture and Rural Affairs Chongqing China; ^5^ Key Laboratory of Agricultural Bioinformatics, Ministry of Education Sichuan Agricultural University Chengdu China

**Keywords:** bulk RNA‐seq, cell–cell communication, heat stress, muscle stem cells, myonuclei, snRNA‐seq

## Abstract

**Background:**

Heat stress can induce skeletal muscle injury. Typical characteristics of heat‐exposed muscle tissues include apoptosis, oxidative stress and autophagy. The understanding of molecular mechanisms underlying heat stress‐induced muscle injury is limited, especially at the single‐cell transcription level.

**Methods:**

We collected skeletal muscles from 12‐week‐old female C57BL/6J mice in control (NC) and four heat stress groups. The experimental scheme comprised five groups: NC (25.5°C ± 0.5°C), HS0 (after ~3‐h heat exposure, 41.5°C ± 0.5°C), HS8, HS16 and HS24 group (recovery at 25.5°C ± 0.5°C for 8, 16 and 24 h, respectively). Skeletal muscles were subjected to HE staining (*n* = 6), TUNEL staining (*n* = 3) and transmission electron microscopy (*n* = 3). Transcripts were measured at the tissue (*n* = 5 or 6) and single‐nucleus levels (*n* = 2).

**Results:**

Histologically, myofibrillar structure deformation, mitochondrial swelling and fusion, intramuscular triglyceride accumulation and autophagy occurred in the muscles subjected to heat stress. At the tissue level, a gene cluster associated with the response to heat exhibited an increasing trend of transcription in the HS0 versus NC groups, and the levels decreased to those in the NC group after 16 h. At the single‐cell level, 134 320 high‐quality myonuclei were collected from the muscles and annotated as seven cell types, including myonuclei, muscle stem cells (MuSCs) and immune cells. We identified the larger number of differentially expressed genes in the myonuclei. After heat stress, new cell clusters appeared in type IIa/IIx (HS8 group) and IIb (HS0 group) myonuclei but not in type I myonuclei. Generally, immediate early genes, highly expressed genes and transcription factor regulons identified in new cell clusters induced by heat were related to responses to heat, heat shock, oxidative stress and antioxidative stress. To repair the injured muscles, MuSCs highly expressed the development‐related genes, such as *Atp2a1*, *Ckm*, *Myh1*, *Aldoa*, *Pde4d* and *Pdlim5*. Analysis of cell–cell communication showed that *Dag1* and *Egf* signals associated with myonuclei were the underlying pathways that participated in repair tissues.

**Conclusions:**

We constructed the largest transcriptomic dataset, to date, for heat‐exposed skeletal muscles. At tissue resolution, the response of muscles to heat stress was eliminated after a recovery period of 16 h. At single‐nucleus resolution, the myofibre was the most heat‐sensitive cell type, and type IIb myonuclei were the most heat‐sensitive subtype of myonuclei. To our knowledge, this is the first study to reveal the molecular mechanisms underlying heat‐induced muscle injury and repair at the single‐cell transcriptional level.

## 1 Introduction

Heat stress (HS) events caused by extreme heat have plagued multiple regions, such as Eastern Asia, Europe, America and East Africa, in recent decades [1, 2, 3]. Severe HS can cause death in humans and animals. Even in individuals who survive HS, intracorporeal damage affects their health [4, 5]. Under high‐temperature conditions, organismal thermoregulation is inadequate, leading to disrupted energy balance [6]. Skeletal muscle, constituting 40%–50% of body mass in adult humans, is key for basal metabolic rate and whole‐body energy metabolism [7]. Thus, skeletal muscles are highly susceptible to heat injury [8, 9]. Two case reports indicated the occurrence of rhabdomyolysis in HS‐exposed patients with body temperature of 40.8°C and 41°C [10]. Cell damage and apoptosis caused by oxidative stress in heat‐exposed muscles cannot be ignored. Chen et al. [11] reported that heat‐exposed mice (41.1°C ± 0.7°C) showed higher levels of reactive oxygen species (ROS) with the activation of heat shock factor 1 and glucocorticoid receptor. Sharma et al. [12] reported that the free radical content of skeletal muscle was higher in rats subjected to HS than in the control group. In the above study, the authors also reported significant correlation of apoptotic proteins, including *caspase‐9*, *caspase‐3*, *Bc12* and *Bax*, with HS in soleus (SOL) and gastrocnemius (Gas). Thus, it is necessary to elucidate the precise mechanisms through which heat‐related injuries affect muscle health.

Although previous studies have provided partial phenotypes and molecular mechanisms for HS‐exposed skeletal muscles, their findings remain accurate only at the organisational level. Skeletal muscle is heterogeneous, comprising multiple cell types, including multiple fibre types, fibroadipogenic progenitors (FAPs), endothelial cells (ECs) and immune cells. The physiological functions of skeletal muscles depend on the cooperation of genes in these cell types. To date, gene expression in heat‐exposed muscles has not been elucidated at single‐cell resolution, which limits our understanding of the molecular mechanisms underlying heat‐induced muscle injury.

Herein, we constructed a bulk RNA‐sequencing atlas and single‐nucleus datasets of heat‐exposed skeletal muscles, including SOL, Gas and tibialis anterior (TA). At the tissue level, we found that the transcriptional response of muscles to HS was eliminated after a 16‐h recovery period. At single‐nucleus resolution, myonuclei displayed higher thermal sensitivity compared with other cell types. Based on this dataset, we clarify, for the first time, the cellular heterogeneity and transcriptional response to HS in skeletal muscles at single‐nucleus resolution.

## 2 Methods

### 2.1 Sample Collection

Animal experiments were approved by the Animal Ethics and Welfare Committee of the Sichuan Agricultural University (animal licence number: 20210189). All efforts were made to reduce animal suffering. Eleven‐week‐old female C57BL/6J mice, purchased from Vital River Laboratories (Beijing, China), were acclimatised for 1 week at the Animal Experiment Centre of Sichuan Agricultural University. A maximum of five mice were cohoused at controlled temperature (25°C ± 0.5°C), relative humidity (RH, 50% ± 10%) and 12‐h light/12‐h dark cycle (light on, 7:00 AM).

The treatment protocol was based on previous studies [11, 12, 13]. The experimental mice were randomly divided into five groups: a negative control (NC) and four HS groups (HS0, HS8, HS16 and HS24). Mice in NC did not receive heat treatment. In HS groups (RH, 50% ± 10%), mice were heat exposed for approximate 3 h, without food and water; this included approximate 1 h of heating to reach a predetermined ambient temperature (41.5°C ± 0.5°C) [11, 12, 14]. At this time point, the caged mice had a core temperature (Tc) > 41°C [15]. We measured body temperature and weight in mice at baseline (NC group; *n* = 9), during heat exposure (60 and 90 min; *n* = 9) and at exposure termination (180 min; HS0 group; *n* = 4). To prevent intermittent heat exposure, mice used for measurements of body temperature and weight at during heat exposure (60 and 90 min; *n* = 9) were not returned to the 41.5°C ± 0.5°C environment for subsequent experiments. Mice in HS0 immediately underwent cervical dislocation, followed by decapitation, and three skeletal muscles, SOL, Gas and TA, were dissected. The mice in the other HS groups resumed their free access to food and water [11, 14]. The SOL, Gas and TA were collected from these mice after 8, 16 and 24 h (the HS8, HS16 and HS24 groups, respectively) of recovery.

All samples were preserved in 4% paraformaldehyde (Biyuntian, China), transmission electron microscopy (TEM) fixative (Servicebio, China) and sterile DNAase/RNase‐free cryovials (Thermo Fisher Scientific, Carlsbad, USA). The skeletal muscle in cryovials was snap‐frozen in liquid nitrogen and stored at −80°C until processing.

### 2.2 Histological Observations

To examine HS‐induced damage, skeletal muscles preserved in 4% paraformaldehyde were used to prepare paraffin sections. The sections were subjected to HE stains (*n* = 6), Masson's trichrome staining (*n* = 9) and apoptosis detection (*n* = 3) using a TUNEL cell apoptosis detection kit (Servicebio, China). Single immunohistochemistry (IHC, *n* = 6) and double immunofluorescence staining (IF, *n* = 6) were used to character thermosensitive populations of myonuclei. Skeletal muscles preserved in TEM fixative were used to observe HS‐induced changes at the subcellular level (*n* = 3). A detailed description of these methods is presented in the Supporting Information.

### 2.3 Bulk RNA‐seq Analysis of Heat‐Exposed Skeletal Muscle

We measured the transcript levels of heat‐exposed skeletal muscles at tissue resolution to observe gene expression patterns. Total RNA was extracted from SOL, Gas and TA obtained from all the NC, HS0, HS8, HS16 and HS24 group (*n* = 6 or 5). A total of 84 high‐quality RNA samples were used to construct cDNA libraries, which were sequenced. The sequences were aligned to the mouse reference genome (*GRCm39*) and used for downstream analyses. A description of the downstream analyses is provided in the Supporting Information.

### 2.4 Heterogeneity of Heat‐Exposed Skeletal Muscle

Eighteen samples were collected from NC, HS0 and HS8 of three tissues (SOL, Gas and TA) for single‐nucleus RNA sequencing (snRNA‐seq) (*n* = 2). cDNA libraries were prepared using a GEXSCOPE Single NucleusRNA‐seq Kit (Singleron Biotechnologies, China). Sequence analysis was performed on the snRNA‐seq libraries using a DNBSEQ‐T7 instrument (BGI, China), and 150‐bp paired‐end raw reads were obtained.

We annotated the skeletal muscle cell types and subtypes according to the expression of known marker genes (Table S1). Raw reads were aligned to the mouse reference genome (*GRCm39*) to generate gene expression matrices for high‐quality nuclei. A high‐quality nucleus expressed no fewer than 300 genes and no more than 10% mitochondrial genes, and a gene that expressed in no fewer than three nuclei was used for downstream analysis [16]. The gene expression values were normalised and scaled based on the NormalizeData and ScaleData functions. Subsequently, batch effects were removed using the Harmony R package (Version 1.0). Finally, cell clusters were defined as distinct cell types based on expression levels of known marker genes (Table S1).

### 2.5 Pseudotime Analysis, Differentiation Potential Prediction and RNA Velocity

The differentiation trajectory was calculated using the DDRTree method in the Monocle2 package to comprehensively characterise the physiological state of muscle stem cells (MuSCs). The known biological background was applied to order the cells when running the orderCells and root_state functions. Branch expression analysis modelling (BEAM) was performed to identify the genes that separated cells into branches.

To predict the relative differentiation state of cells, we performed CytoTRACE (v0.1.0) using default parameters (https://cytotrace.stanford.edu). A CytoTRACE score closer to 1 indicates a lower degree of differentiation, whereas that closer to 0 indicates a higher degree of differentiation. RNA velocity was calculated using the velocyto R package (https://github.com/velocyto‐team/velocyto.R).

### 2.6 Gene Expression Characteristics

To identify the association between highly collaborative gene sets and phenotypes, the high‐dimensional weighted gene coexpression network analysis (hdWGCNA) R package (v0.1.1.9002) was used for coexpression analysis (https://github.com/smorabit/hdWGCNA). First, the MetacellsByGroups function was used to construct expression matrices for the same cell types from different samples. Then, coexpression network analysis was performed using the ConstructNetwork function. Finally, the modules eigengenes and connectivity were successively computed using the ModuleEigengenes and ModuleConnectivity functions.

To decipher the gene transcription dynamics in heat‐exposed muscle cells, we identified the differentially expressed genes (DEGs) between adjacent groups. Subsequently, we investigated the expression levels of immediate early genes (IEGs) in part of cell types (Table S2). Additionally, the time‐series expression profiles were construction using Mufzz algorithm. The metabolic activity of the heat‐exposed skeletal muscles was quantified using the VISION method and Kyoto Encyclopedia of Genes and Genomes (KEGG) pathways built into scMetabolism (v0.2.1).

### 2.7 Analysis of Transcription Factor (TF) Expression

Single‐Cell Regulatory Network Inference and Clustering (SCENIC) was performed in R to reconstruct gene‐regulatory networks (GRNs) and identify the TF activity of cell types and subtypes. The SCENIC workflow was run as described on the SCENIC website (https://github.com/aertslab/SCENIC).

### 2.8 Cell–Cell Interactions (CCIs)

The physiological function of the skeletal muscle depends on the communication between various cell types and subtypes. We detected CCIs of seven cell types using the CellChat algorithm to decipher the molecular mechanisms after HS exposure.

### 2.9 Statistical Analysis

Statistical analysis of phenotypic characteristics was performed using the GraphPad Prism 9 software (GraphPad, USA) and one‐way analysis of variance (ANOVA). Data are presented as mean ± standard deviation (SD), and *p* < 0.05 (*) indicates a significant difference.

## 3 Data Availability

Details of data availability are provided in the Supporting Information.

## 4 Results

### 4.1 Histology of the HS‐Exposed Skeletal Muscle

**FIGURE 1:**
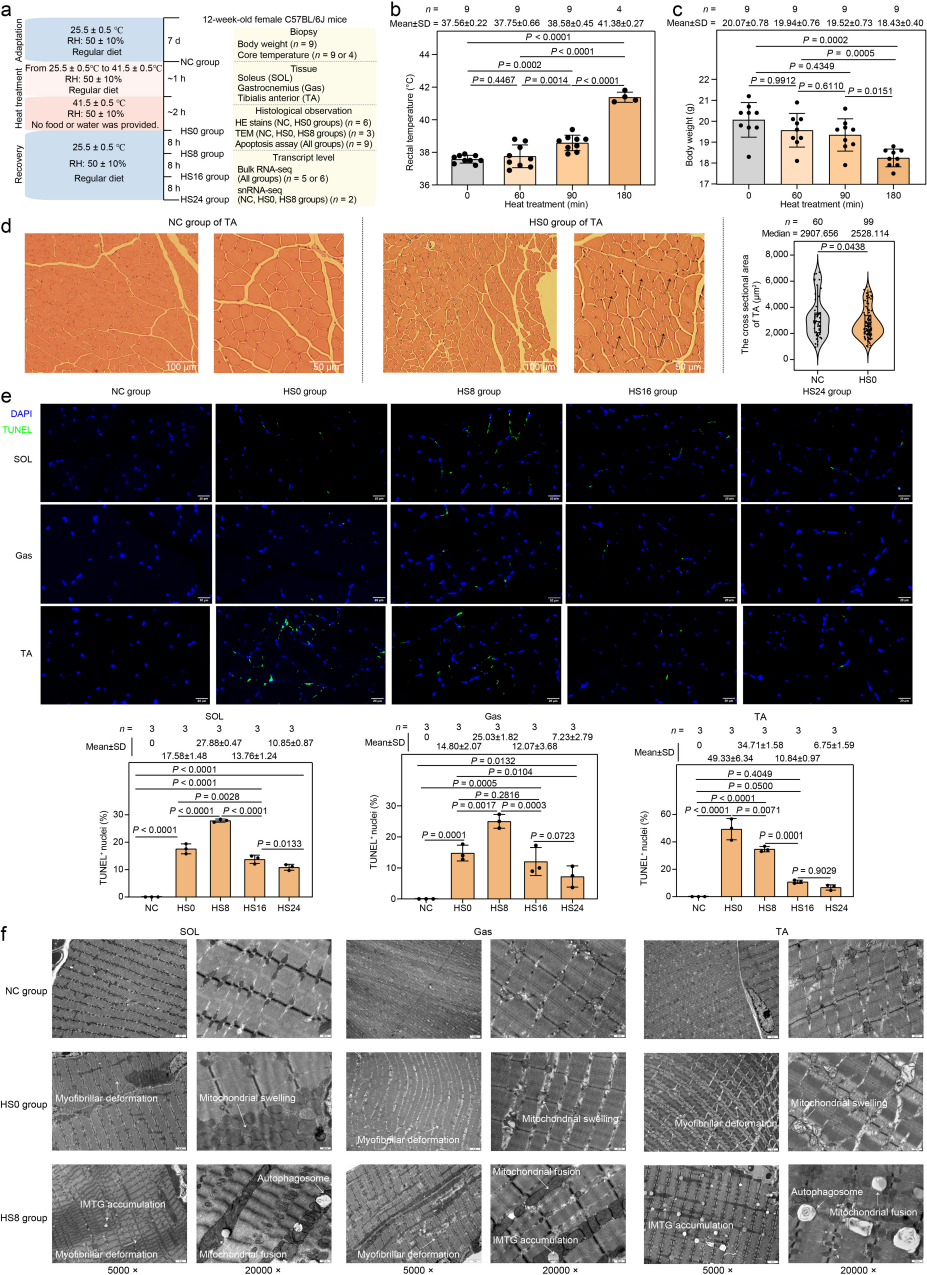
Workflow and phenotypic assessment. (a) Experimental protocol for heat stress and phenotypic assessment. The sample size was described in various determinations. (b) Ambient temperature monitoring of mice before (0 min; NC group), during (60 and 90 min) and after (180 min; HS0 group) heat treatment. (c) Rectal temperature of mice before (0 min; NC group), during (60 and 90 min) and after (180 min; HS0 group) heat treatment. (d) Representative photomicrographs of tibialis anterior (TA) stained with haematoxylin and eosin (HE). The black arrows indicated myolysis (middle panel). Cross‐sectional area (CSA) of TA based on HE staining (right panel). The values ‘60’ (NC group) and ‘99’ (HS0 group) represented the total number of myofibres for cross‐sectional area measurements. Myofibres were derived from three biological replicates, each with two technical replicates (*n* = 6). The CSA of each myofibre was shown in Table S5. (e) Immunofluorescence images showing apoptosis of cells in skeletal muscles (TUNEL staining) in the NC and two treated groups. Normal nuclei were stained with DAPI (blue) and apoptotic nuclei were stained with TUNEL (green). The value of *n* represented the number of nuclei counted for the apoptosis rate assay across all groups (NC, HS0, HS8, HS16 and HS24). These myonuclei were obtained from three biological replicates (*n* = 3). Apoptosis rate of each biological replicate was shown in Table S8. (b–e) Data were presented as mean ± standard deviation (SD), and *p* < 0.05 (*) indicates a significant difference. (f) Subcellular structures of skeletal muscles visualised using transmission electron microscopy.

We collected SOL, Gas and TA from NC, HS0, HS8, HS16 and HS24 group (Figure 1a). In HS0 group, the core temperature of mice gradually increased to 41.38°C ± 0.27°C (Figure 1b and Table S3), and the mice exhibited a significant weight loss (*p* = 0.0002) compared with NC mice (Figure 1c and Table S4). Histological examination confirmed myolysis (Figures 1d and S1). The cross‐sectional area (CSA) of skeletal muscles significantly decreased (SOL, *p* < 0.0001; Gas, *p* = 0.0013; TA, *p* = 0.0438) after heat treatment (Figures 1d and S1 and Tables S5–S7), which align with a previous observation [12]. The apoptosis rate of skeletal muscle significantly increased in HS0 versus NC group, and the apoptosis rate in HS0 group was higher for SOL than for Gas (Figure 1e and Table S8), consistent with a previous study in rats [12]. The apoptosis rate in glycolytic TA reached a maximum in HS0 whereas that for oxidative SOL and Gas was maximum in HS8, indicating that the response mechanisms to HS differ between oxidative and glycolytic muscles. At the subcellular level, the muscle showed myofibrillar structural deformation, mitochondrial swelling and fusion, intramuscular triglyceride accumulation and autophagy immediately after heat treatment (Figure 1f), implying that the skeletal muscle suffered HS‐induced oxidative stress, as reported previously [11].

### 4.2 Transcriptional Profile of Heat‐Exposed Skeletal Muscle at Bulk RNA‐seq Level

**FIGURE 2:**
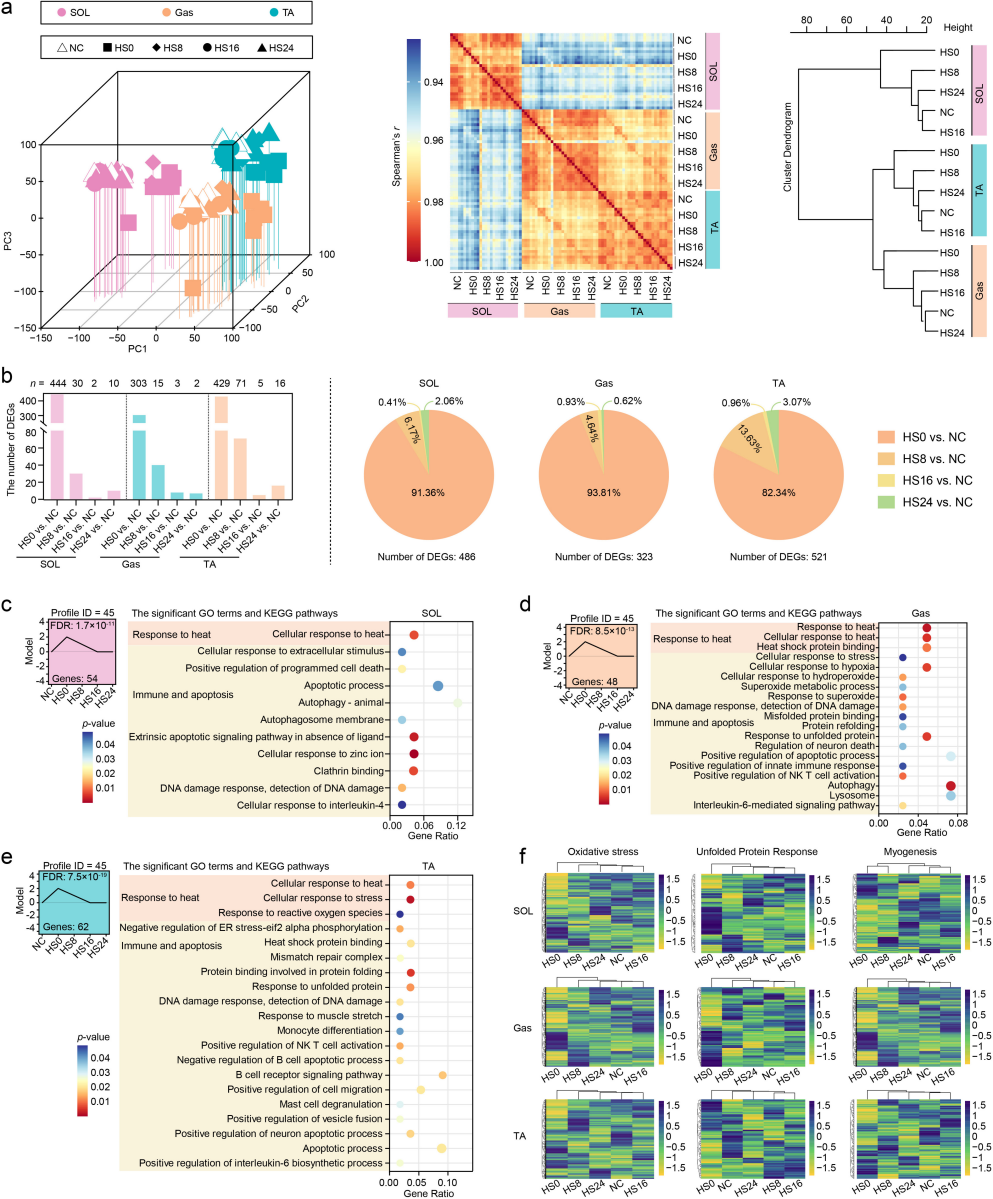
Expression profiling of mice skeletal muscles using bulk RNA sequencing. (a) Principal component analysis (left panel), Spearman coefficient (middle panel) and Euclidean distance (right panel) of mRNA levels revealed heterogeneity in SOL, Gas and TA samples in both physiological and heat‐exposed groups (*n* = 5 or 6). (b) The distribution of 1421 differentially expressed genes (*p* value < 0.05 and |fold change| > 2) from the three tissues in four comparison groups (HS0 vs. NC group, HS8 vs. NC group, HS16 vs. NC group and HS24 vs. NC group) (left panel). The proportion of individual comparison group in each tissue (right panel). (c–e) Function analysis of the significant gene expression patterns (Profile 45) identified for samples of SOL, Gas and TA using the short time‐series expression miner (STEM) analysis. (f) Spearman correlation coefficient for each skeletal muscle among the five groups was calculated based on the expression of genes related to oxidative stress, unfolded protein response and myogenesis.

To investigate the transcriptional response to HS, we isolated SOL, Gas and TA muscles from bilateral hindlimbs of five groups (NC, HS0, HS8, HS16 and HS24 groups; *n* = 6 or 5) for RNA extraction and library construction. A total of 977.49‐Gb high‐quality data were generated using the 84 bulk RNA‐seq libraries. We detected expression of 15 206 genes in this atlas. Pairwise Spearman correlation analysis of biological replicates demonstrated exceptionally high reproducibility (*r* > 0.96) (Table S9). At the global transcriptional level, the results of PCA (Figure 2a, left panel), Spearman's coefficient (Figure 2a, middle panel) and Euclidean distance (Figure 2a, right panel) indicated different patterns across skeletal muscles; however, there were relatively similar patterns among NC, HS16 and HS24 group within a tissue (Figure 2a, right panel). Most of these changes were observed for HS0 versus NC group and then gradually decreased over time. In detail, more than 300 DEGs (82.34%–93.81%) (*p* value < 0.05 and |fold change| > 2) were identified between NC and HS0 group, and the number of DEGs in other comparison groups was fewer than 72 (HS8 vs. NC group, HS16 vs. NC group and HS24 vs. NC group) (Figure 2b, left panel). At 16 h postheat treatment (HS16 group), transcriptomic profiles returned to baseline (NC group levels). Gene expression exhibited consistency across NC, HS16 and HS24 groups in each tissue, with minimal DEGs. DEG proportions (SOL: 2.47%; Gas: 1.55%; TA: 4.03%) were no more than 4.03% identified in two comparison group (HS16 vs. NC group; HS24 vs. NC group), with intertissue variation not exceeding 2.11% (Figure 2b, right panel).

We modelled the transcriptional patterns over time using all expressed genes with STEM. Notably, Profile 45 showed an increasing trend after heat treatment (HS0 group) and then decreased to the NC group level after 16 h (HS16 group) in the SOL, Gas and TA (Figure 2c–e). Functional enrichment analysis of these genes revealed a HS response. Genes with known functions, oxidative stress, unfolded protein response and myogenesis genes displayed similar expression patterns between NC and HS16 for each skeletal muscle (Figure 2f). These results implied that the response of muscles to HS was eliminated at the tissue level in HS16 group.

### 4.3 Transcriptional Response to HS in Skeletal Muscles at Single‐Nucleus Level

**FIGURE 3:**
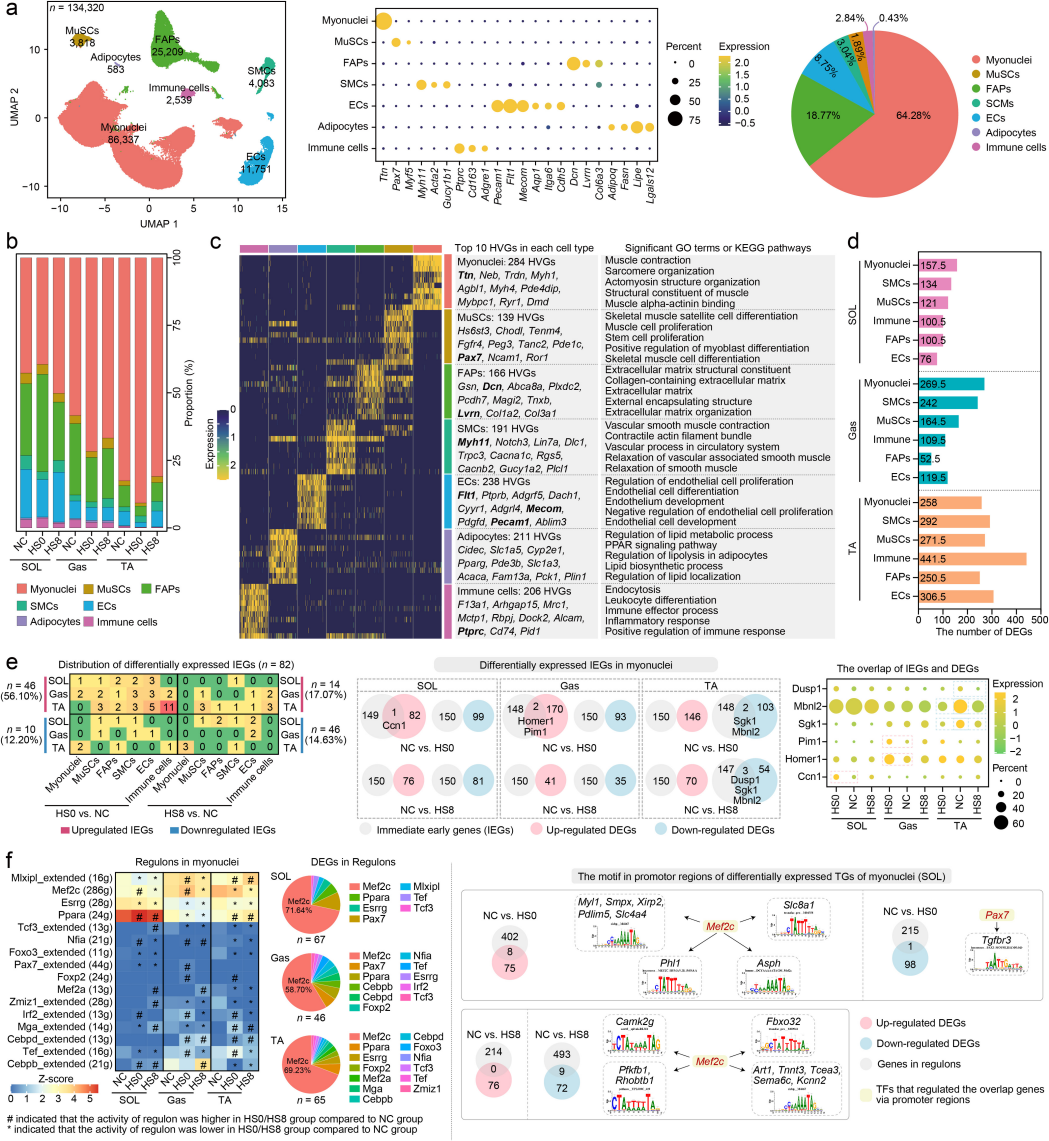
A single‐nucleus atlas of the mice skeletal muscles. (a) Uniform manifold approximation and projection (UMAP) visualisation of myonuclei, muscle stem cells (MuSCs), fibroadipogenic progenitors (FAPs), smooth muscle cells (SMCs), endothelial cells (ECs), adipocytes and immune cells (left panel). The expression levels of known maker genes in the seven cell types (middle panel). The pie chart showing the proportions of the seven cell types (right panel). These high‐quality nuclei were isolated from SOL, Gas and TA of NC, HS0 and HS8 groups (*n* = 2). (b) The proportion of the seven cell types in each group. (c) Functional analysis of the high variable genes (HVGs) in each cell type. (d) The number of differentially expressed genes (DEGs) in six cell types during heat treatment (HS0 vs. NC group) and 8 h of recovery (HS8 vs. HS0 group) after heat treatment. (e) Differentially expressed IEGs of myonuclei in SOL, Gas and TA. The heatmap represented the number of differentially expressed IEGs in the HS0 versus NC groups and HS8 versus NC groups of SOL, Gas and TA (left panel). The expression of differentially expressed IEGs of myonuclei (middle and right panel). (f) Regulons identified in Myonuclei. Mef2c regulon of myonuclei included abundant DEGs (left panel). Motifs in the promoter regions of DEGs identified from SOL binding by Mef2c (right panel).

To explore heat‐induced skeletal muscle injury at single‐nucleus level, samples from NC, HS0 and HS8 groups were assembled into a unified transcriptomic atlas containing 134 320 high‐quality nuclei expressing, on average, 831 unique molecular identifiers (UMIs) and 543 genes per nucleus (Figure 3a, left panel, and Figure S2a). Based on the expression levels of known marker genes (Figure 3a, middle panel; Figure S2b; and Table S1), we annotated myonuclei (64.28%), FAPs (18.77%), ECs (8.75%), smooth muscle cells (SMCs, 3.04%), MuSCs (2.84%), immune cells (1.89%) and adipocytes (0.43%) (Figure 3a, right panel), which were identified previously [17]. Seven cell types were identified in each group (Figure 3b and Tables S10 and S11). We also dissected the hypervariable genes (HVGs) (|fold change| > 1.5, adjusted *p* < 0.05) (Figure 3c) and cell type‐specific modules (hdWGCNA algorithm). Except for adipocytes, the other six cell types corresponded to one or two gene modules (Figure S2c–e). The GO analysis for each module was appropriate for the cell type, indicating the accuracy of cell‐type annotation.

We identified DEGs between neighbouring pairwise time points for each cell type, except for adipocytes (owing to their rare number) (Figure 3d and Table S11). The largest number of DEGs was identified in the myonuclei isolated from SOL and Gas, which indicated that the myonuclei of SOL and Gas were extremely sensitive to heat. The transcriptional changes in most cell types differed between tissues, with TA (1820 DEGs) showing greater changes than SOL (690 DEGs) and Gas (958 DEGs). This result showed that TA exhibited a relatively intense transcriptional response after approximately 3 h of HS. This phenomenon potentially correlated with more sensitive motor units in TA than in SOL and Gas [18]. Moreover, the DEGs between pairwise time points rarely overlapped across the six cell types (Figure S3), which highlighted their distinct transcriptional responses to heat.

We profiled 150 canonical IEGs (Table S2) and identified 82 that were differentially expressed in the three skeletal muscles suffered HS (HS0/HS8 vs. NC groups), revealing the activation of rapid response program. A majority of differentially expressed IEGs (73.17%, 60/82) were upregulated (Figures 3e and S4a and Table S12). The upregulated IEGs were involved in stress response (*Ccn1* [S1] and *Serpine1* [S2]), muscle atrophy (*Txnip* [S3] and *Pim1* [S4]) and immune response (*Cebpd* [S5]) (Figure 3e, middle and right panel). For example, spinal cord injury‐induced reduction in Gas fibre CSA is accompanied by an increase in *Pim1* expression [S4].

We applied the SCENIC algorithm to infer and quantify gene‐regulatory networks in myonuclei. The 16 regulons exhibiting differential activity between groups (Figure 3f, left panel). A total of 178 DEGs were identified overlap with target genes of the regulons. Notably, over half of DEGs were regulated by Mef2c (Figure 3f, left panel, and Tables S13–S15). Typically, genes associated with myofibre structure maintenance such as *Fbxo32* [S6], *Pfkfb3* [S7] and *Tnnt3* [S8], harboured the Mef2c motif in their promoter regions, were also differentially expressed under HS (Figure 3f, right panel, and Figure S4b).

### 4.4 Myonuclei Responded to HS

**FIGURE 4:**
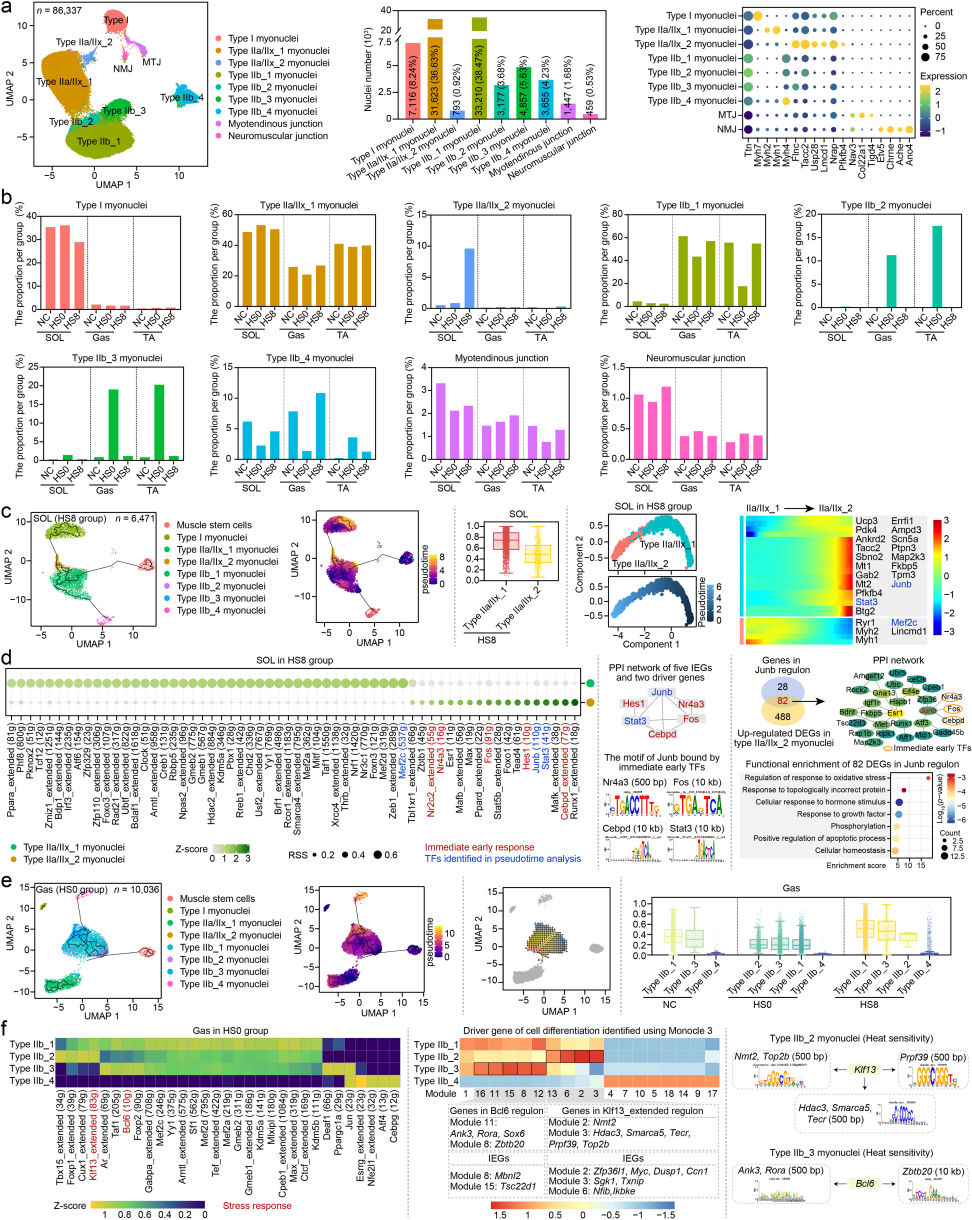
A single‐nucleus atlas of the heat‐exposed mice myonuclei. (a) Annotation of myonuclei subtypes. Uniform manifold approximation and projection (UMAP) visualisation of myonuclei (left panel). Proportion of myonuclei subtypes (middle panel). The expression levels of known maker genes in the myonuclei subtypes (right panel). (b) Proportion of myonuclei in each group. (c) The differentiation trajectory of muscle stem cells (MuSCs) and myonuclei in the HS8 group of SOL (left panel). The cell entropy of type IIa/IIx_1 and type IIa/IIx_2 myonuclei (middle panel). The differentiation trajectory and driver genes of Type IIa/IIx_1 and Type IIa/IIx_2 myonuclei (right panel). (d) Assessment of regulon activity using single‐cell regulatory network inference and clustering (SCENIC). Dot plot showing the specific regulons of type IIa/IIx_1 and type IIa/IIx_2 myonuclei (left panel). The protein–protein interaction (PPI) network of five IEGs and two driver genes of cell differentiation (middle panel). The PPI network and functional enrichment analysis of Junb regulon (right). (e) The differentiation trajectory (left panel) and RNA velocity (middle panel) of muscle stem cells (MuSCs) and myonuclei in the HS0 group of Gas. The cell entropy of type IIb myonuclei of Gas in SOL, Gas and TA (right). (f) The expression of regulons in type IIb myonuclei of Gas (left panel). The driver genes of cell differentiation and IEGs in regulons involved in stress response (middle panel). The predicted motif sites between TFs (*Klf13* and *Bcl6*) related to stress response and driver genes of differentiation of type IIb_2 and type IIb_3 myonuclei (right panel).

Myofibres are extremely heat‐sensitive parenchymal cells of skeletal muscles [8]. Myonuclei (*n* = 86 337, accounting for 39.48%–90.79% of the total nuclei) could be further clustered into nine groups, functionally distinguished into five myonuclei types, including type I myonuclei, type IIa/IIx myonuclei, type IIb myonuclei, myotendinous junction (MTJ) and neuromuscular junction (NMJ), by expression of MYHs and marker genes in previous reports (Figures 4a and S5a,b and Table S1) [17, 19, 20, 21, 22, 23]. The proportion of five myonuclei types was distinct across the skeletal muscles, with type I myonuclei mainly existing in oxidative muscle–SOL (Figure 4b), whereas type IIb myonuclei mainly existed in glycolytic muscle–Gas and TA (96.45%). Type IIa/IIx myonuclei were annotated in all tissues and showed subtle differences across SMTs (Table S16). The proportion of MTJ/NMJ ranged from 0.76%/0.28% to 3.32%/1.19% (Figure 4b).

Type IIa/IIx myonuclei included two subtypes, type IIa/IIx_1 and type IIa/IIx_2 myonuclei (Figure 4a). Type IIa/IIx_1 myonuclei highly expressed typical marker genes of type IIa/IIx myofibres, including *Ttn*, *Myh2* and *Myh1*. Type IIa/IIx_2 myonuclei showed high expression levels of *Ttn*, *Flnc*, *Tacc2*, *Usp28*, *Lmcd1*, *Nrap*, *Pfkfb4* and *Stat3* (Figures 4a and S6a–c). Type IIa/IIx_1 showed no difference in proportion between treatment groups, whereas type IIa/IIx_2 myonuclei mainly existed in the SOL from HS8 group (73.64%), and the number of this type of myonuclei in HS8 group was 16.69/11.23 times that in NC/HS0 group. This suggested that the type IIa/IIx_2 population is potentially thermosensitive. The expression pattern of maker genes for type IIa/IIx_2 myonuclei was similar to the ‘unidentified myonuclei’ labelled by *Ttn*, *Flnc*, *Tacc2*, *Usp28*, *Lmcd1*, *Nrap* and *Pfkfb4* identified in denervated muscles by Dos Santos et al. [17]. The ‘unidentified myonuclei’ could correspond to myonuclei in the area of myofibre damage to support fast repair [17, 24, 25].

We calculated the differentiation trajectories of MuSCs and myofibres collected from SOL in HS8 group. Type IIa/IIx_2 myonuclei arose from type IIa/IIx_1 myonuclei (Figure 4c, left and middle panels). We characterised two populations of type IIa/IIx_2 myonuclei using the function of HVGs (Figure S5c). Type IIa/IIx_1 myonuclei highly expressed genes that maintain muscle physiological functions, such as muscle system processes, actin binding and the growth of myofibrils, contractile fibres and sarcomeres. Besides genes that maintain muscle physiological function, type IIa/IIx_2 myonuclei highly expressed HVGs associated with cellular responses to heat, unfolded proteins, osmotic stress, apoptotic signalling pathways and myotube differentiation. We identified 570 upregulated (including 27 IEGs) and 412 downregulated DEGs (fold change > 1.5 or < 0.5 and *p* value < 0.05) in type IIa/IIx_1 versus type IIa/IIx_2 myonuclei (Figure S5d and Table S17). Subsequently, we identified the driver genes for this process during the transformation of physiological IIa/IIx (IIa/IIx_1) myonuclei into heat‐sensitive myonuclei (IIa/IIx_2) (Figure 4c, right panel). Moreover, using the SCENIC algorithm, we identified TF networks potentially regulating multiple biological processes. In type IIa/IIx_1 myonuclei, the active TF regulons were predicted to participate in cell development (Mef2c regulon) and fatty acid metabolism (Ppara_extended regulon) (Figure 4d, left panel). Active TF regulons were predicted to be involved in inflammatory response (Stat3, Stat5b_extended and Cebpd_extended regulons), apoptosis (Fos, Foxo1 and Nr4a3 regulons) and cell development (Hes1 and Tead4 regulons) in type IIa/IIx_2 myonuclei (Figure 4d, left panel). Among these TFs, we identified two genes that promote the formation of type IIa/IIx_2 myonuclei (*Stat3* and *Junb*) and four IEGs (*Nr2c2*, *Nr4a3*, *Fos*, *Hes1* and *Cebpd*) (Table S18). Although analysis based on the STRING database suggested that *Stat3* and *Junb* could be regulated by multiple IEGs (*Nr4a3*, *Fos*, *Hes1* and *Cebpd*), only motif binding sites for *Junb* were detected on the loci of *Nr4a3*, *Fos* and *Cebpd* in type IIa/IIx_2 myonuclei (heat‐sensitive myonuclei) (Figure 4d, middle panel). We subsequently focused on the function of 110 genes within the Junb regulon, in which 82 genes were upregulated DEGs in type IIa/IIx_2 versus type IIa/IIx_1 myonuclei. These 82 upregulated DEGs were primarily involved in the regulation of response to oxidative stress, response to topologically incorrect protein, cellular homeostasis and the positive regulation of the apoptotic process (Figure 4d, right panel).

Type IIb myonuclei were classified into four subtypes: type IIb_1, type IIb_2, type IIb_3 and type IIb_4 (Figures 4a and S6d–f). We characterised the four subtypes of myonuclei using the functions of HVGs (Figure S5c). HVGs expressed in type IIb_1 myonuclei play basic physiological roles, including fast‐twitch myofibres (*Myh4* and *Actn3*) [S9, S10], muscle structure development (*Casq1* and *Tpm1*) [S11, S12] and myofibril maintenance (*Myom2*, *Mybph* and *Mylk2*) [S13–S15]. In addition to the basic functions of muscles, HVGs of type IIb_2 myonuclei perform supramolecular fibre organisation (*Mapt*) [S16] and form polymeric cytoskeletal fibres (*Pdlim5*) [S17]. Type IIb_3 and type IIb_4 myonuclei highly expressed classic HS‐associated terms or pathways, including response to heat (*Hikeshi*, *Hspb1* and *Hsp90aa1*) [S18–S20], heat shock protein binding (*Hspa8* and *Hsp90*) [S21, S22], response to oxidative stress (*Sesn1* and *Sesn3*) [S23, S24] and macroautophagy (*Bag3*) [S25]. A critical difference between the two subpopulations was that type IIb_3 myonuclei expressed apoptosis‐related genes (*Trim63*) [S26].

For Gas and TA, type IIb_2 and IIb_3 myonuclei were observed primarily in HS0 group (IIb_2: 89.93%; IIb_3: 99.81%). This indicated type IIb_2 and IIb_3 myonuclei with thermosensitivity (Figure 4b). We used diffusion pseudotime analysis to assign pseudotime coordinates to MuSCs and myonuclei from HS0 group, given a root cell selected from MuSCs. MuSCs did not differentiate into type IIb_2, IIb_3 or IIb_4 myonuclei (Figure 4e). Type IIb_2 and IIb_3 myonuclei differentiated from type IIb_1 myonuclei. Type IIb_3 myonuclei differentiated into IIb_4 myonuclei.

We also calculated the activity of TF regulons in type IIb_1, IIb_2, IIb_3 and IIb_4 myonuclei (Figure 4f, left panel) using the SCENIC algorithm. In type IIb_1 myonuclei, the active TF regulons were associated with cell development and growth. In type IIb_3 myonuclei, active TF regulons played a role in adaptive thermogenesis, such as *Ppargc1a* [S27]. In type IIb_2 myonuclei, maintenance of glycolytic myofibre number (Tbx15_extended), cellular senescence (Foxp1_extended) and stress response (Klf13_extended) showed high activation compared with the other three types of type IIb myonuclei. In type IIb_4 myonuclei, active TF regulons exert an anti‐inflammatory response (*Cebpbg*) [S28], redox balance (*Nfe2l1*) [S29], antioxidative stress and anti‐autophagy (*Atf4*) [S30]. The function of these TF regulons is consistent with that of HVGs in type IIb myonuclei. There were two TF regulons involved in stress response (Klf13_extended and Bcl6 regulons) (Figure 4f, left panel). The Klf13_extended and Bcl6 regulons were highly expressed in type IIb_2 and type IIb_3 myonuclei, respectively. Within these TF regulons, six and four genes exhibited elevated expression specifically in type IIb_2 myonuclei (*Nmt2*, *Hdac3*, *Smarca5*, *Tecr*, *Prpf39* and *Top2b*) or type IIb_3 myonuclei (*Ank3*, *Rora*, *Sox6* and *Zbtb20*) (Figure 4f, middle panel). The motif enrichment predicted binding sites for *Klf13*, through which it potentially regulates a cohort of genes (*Nmt2*, *Top2b*, *Hdac3*, *Smarca5*, *Tecr* and *Prpf39*) implicated in type IIb_2 myonuclei differentiation (Figure 4f, right panel). Similarly, the analysis predicted *Bcl6* binding sites associated with another gene set (*Ank3*, *Rora* and *Zbtb20*) involved in type IIb_3 myonuclear formation (Figure 4f, right panel).

### 4.5 MuSCs Responded to HS

**FIGURE 5:**
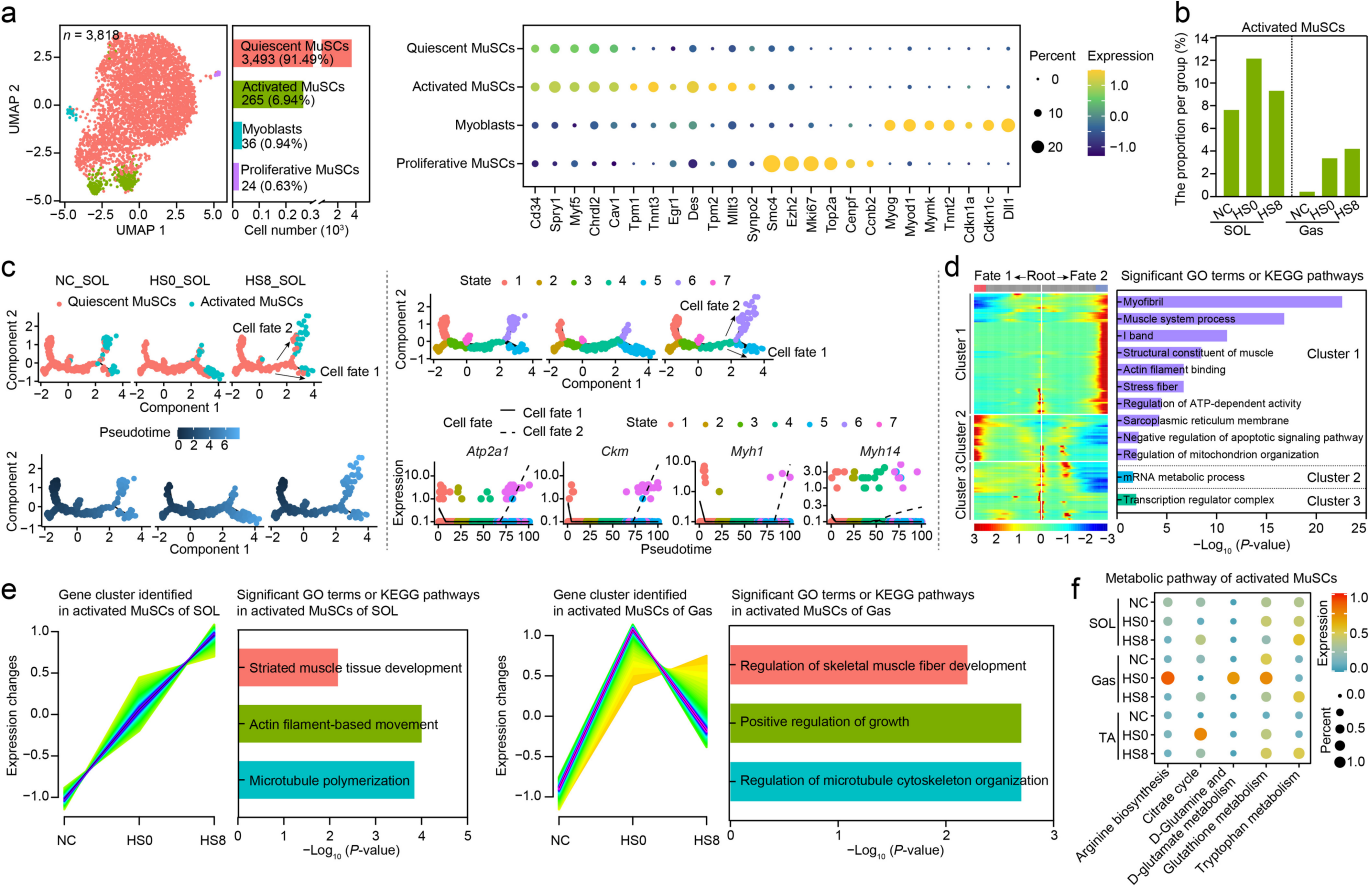
Muscle stem cells (MuSCs) participate in tissue repair after heat treatment. (a) The four subtypes of MuSCs and their proportions (left panel). The expression of known maker genes in the four subtypes of MuSCs, including quiescent MuSCs, activated MuSCs, myoblasts and proliferative MuSCs (right panel). (b) Proportion of activated MuSCs per group. (c) Differentiation trajectories (left panel) and diver genes (middle panel) from quiescent to activated MuSCs collected from SOL. Activated MuSCs were predominantly localised to State 5 (Cell Fate 1) and State 6 (Cell Fate 2). (d) GO terms and KEGG pathway analyses of the two states of activated MuSCs (right panel). (e) GO term and KEGG pathway analyses indicated that these genes play roles in skeletal muscle development. (f) Metabolic pathway analysis of activated MuSCs.

Heat exposure induces apoptosis in skeletal muscles, which could induce MuSCs proliferation and differentiation into mature myofibres to repair damage. To investigate the roles of MuSCs in response to HS, we reclustered (*n* = 3818) and separated them into four clusters corresponding to four subpopulations: quiescent MuSCs (*n* = 3493, 91.49%) (*Cd34*, *Spry1*, *Myf5*, *Chrdl2* and *Cav1* [26, 27]), activated MuSCs (*n* = 265, 6.94%) (*Tpm1*, *Tnnt3*, *Egr1*, *Des* and *Tpm2* [28, 29]), proliferative MuSCs (*n* = 24, 0.63%) (*Smc4*, *Ezh2*, *Mki67*, *Top2a*, *Cenpf* and *Ccnb2* [29, 30]) and myoblasts (*n* = 36, 0.94%) (*Myog*, *Myod1*, *Mymk*, *Tnnt2*, *Cdkn1a*, *Cdkn1c* and *Dll1* [30, 31]) (Figure 5a and Tables S1 and S19). The HVG functions of the four subtypes of MuSC underpinned our subtype annotation (Figure S7a). In addition, the results of RNA velocity showed that entropy was always higher in quiescent MuSCs than in activated MuSCs, except for TA from NC group, which suggested that quiescent MuSCs possessed a natural aptitude for differentiation rather than activated MuSCs (Figure S7b), consistent with their known properties [31]. This bias could be attributed to the extremely low proportion (less than 0.1%) of activated MuSCs in TA (Figure S8). Given the characteristically low abundance of activated MuSCs in TA, we excluded them from subsequent analysis [17]. In summary, the function of HVGs, velocity profiles and cell entropy support the MuSC subtype annotation.

Upon injury, activated MuSCs mediate skeletal muscle repair [11]. The number of activated MuSCs from SOL and Gas was higher in the HS groups than in NC group (Figure 5c). To evaluate the differentiation trend, pseudotime trajectories of quiescent and activated MuSCs were constructed using the Monocle 2 algorithm. The trajectory exhibited higher differentiation‐activated MuSCs from SOL and Gas in HS0 and HS8 than in NC group (Figure 5c, left panel, and Figure S7c, left panel). We identified the genes driving the differentiation process from quiescent to higher differentiation‐activated MuSCs from SOL and Gas (Table S20). In SOL, the driver genes included *Atp2a1*, *Ckm*, *Myh1*, *Myh14* and *Ttn* [19, 22, 32] (Figure 5c, right panel). *Aldoa* [S31], *Pdlim5* [S32] and *Snta1* [S33] were the drivers in Gas (Figure S7c, middle panel). These are key genes involved in skeletal muscle repair after heat‐induced injury. Additionally, we identified two distinct activated MuSC populations (Cell Fate 1: State 5; Cell Fate 2: State 6) on the pseudotime trajectory (Figure 5c). Gene Ontology (GO) and KEGG pathway analyses revealed that highly expressed genes in Cell Fate 1 were primarily associated with mRNA metabolic processes, whereas those in Cell Fate 2 were predominantly involved in skeletal muscle development (Figure 5d). Next, we used the Mufzz algorithm to identify time‐series expression profiles. For SOL and Gas, genes in Cluster 1 showed higher expression in HS0 and HS8 than in NC group. GO term and KEGG pathway analyses indicated that these genes play roles in skeletal muscle development (Figure 5e). The scMetabolism package was used to assess cell metabolic activity. We found that some anti‐HS substances and pathways were activated in HS groups of activated MuSCs (Figure 5f), including tryptophan [S34], glutathione [S35], d‐glutamine and d‐glutamate [S36], citrate cycle [S37] and arginine [S38].

### 4.6 Cellular Interactions Revealed the Reactions in Heat‐Induced Repair and Collagen Synthesis

**FIGURE 6:**
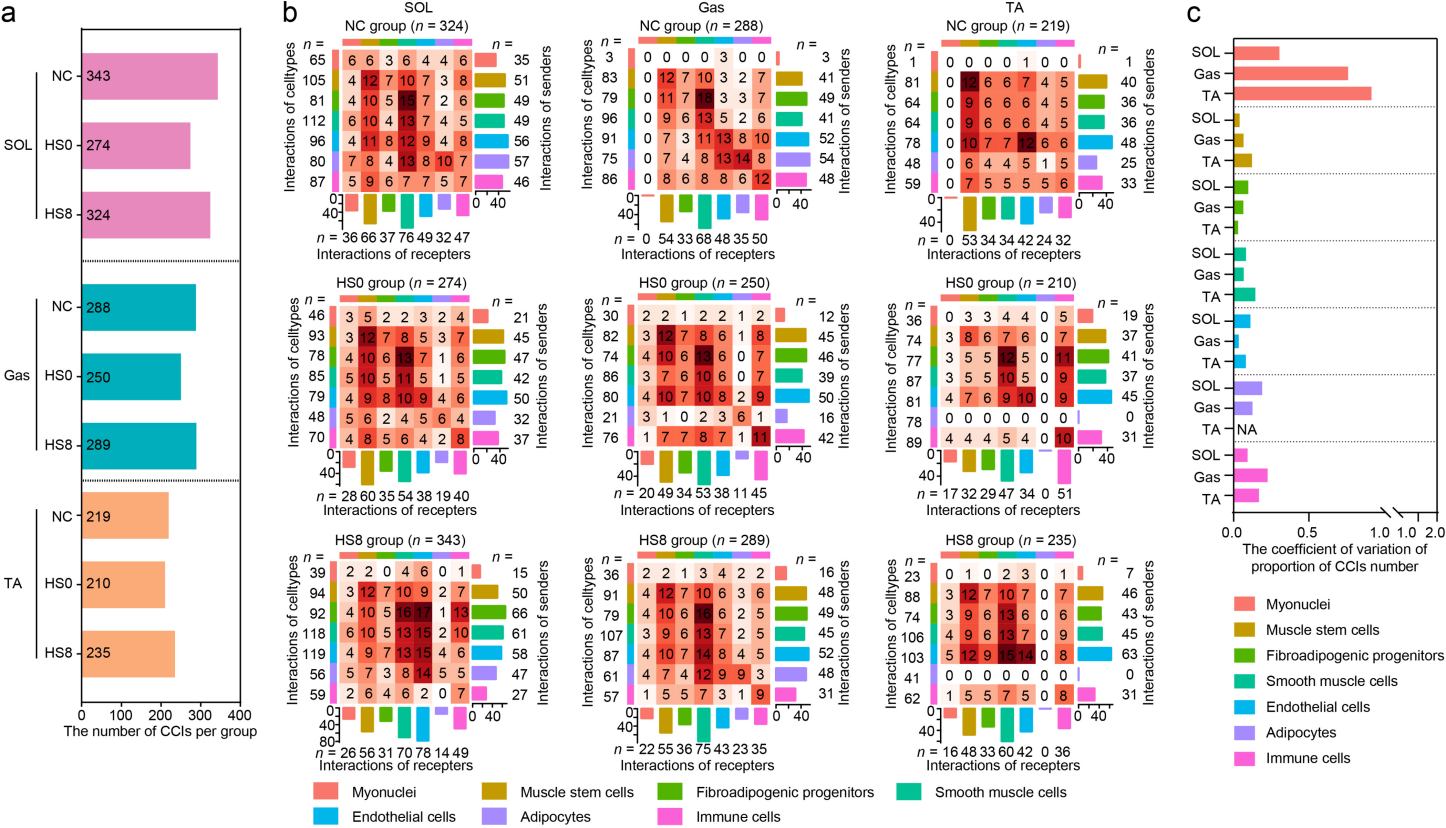
Analysis of the cellular communication network in heat‐exposed skeletal muscles. (a) The number of cell–cell interactions (CCIs) for each group. (b) Heatmap showing the number of interactions among the seven cell types. (c) The coefficient of variation distribution of CCI proportion. ‘NA’ represents a value with no statistical significance.

We calculated the significantly correlated ligand–receptor (L–R) pairs (*p* < 0.05) using CellChat algorithm. The number of CCIs in HS0 group that suffered the more severe stress was lower compared with that in NC and HS8 group (Figure 6a). Moreover, the variation in CCI numbers across groups was greater between myonuclei and other cell types than between other cell types (Figure 6b,c and Table S21). Therefore, we focused on the crosstalk between myonuclei to unveil these variations.

**FIGURE 7:**
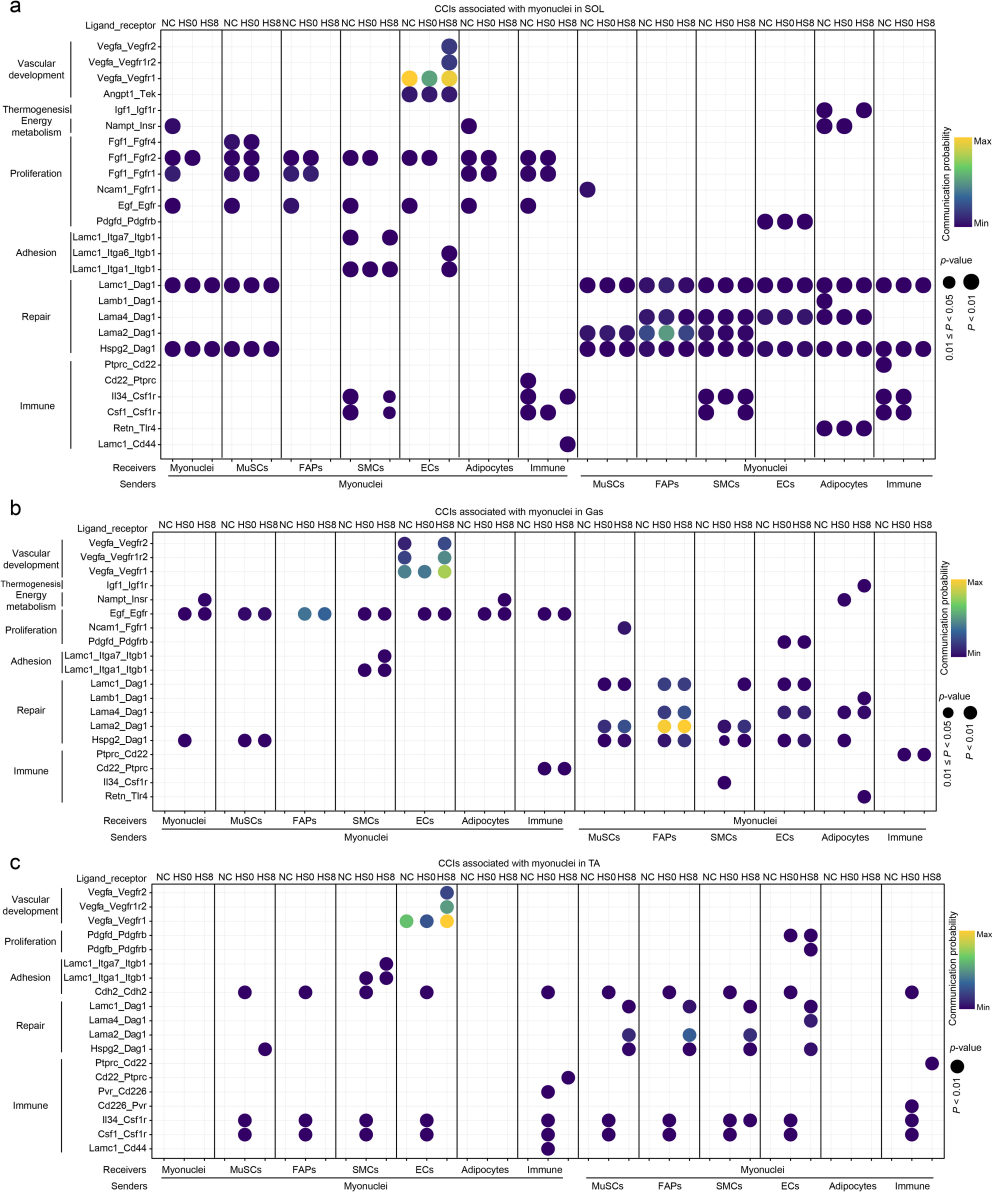
Bubble plots showing the interaction profiles and expression patterns of interaction pathways identified in myonuclei of soleus (SOL), gastrocnemius (Gas) and tibialis anterior (TA).

No significant differences were noted in the category or function of CCIs in the three muscles (Figure 7). These CCIs mainly participate in vascular development (*Vegfa*–*Vegfr*) [S39], proliferation (*Pdgfd*–*Pdgfrb*) [S40–S43], integrin‐mediated adhesion (*Itga1* and *Itga7*) [S44, S45], tissue repair (ligand–*Dag1*) [S46] and immunity (*Cd22*, *Ptprc* and *Csf1r*) [S47]. However, the temporal expression patterns of these common CCIs were distinct in the three muscles. For example, the *Egf*/*Fgf*‐induced proliferation signals were inhibited in SOL, and activated in Gas, and not detected in TA after HS (HS0 and HS8 group). The repair signal mediated by *Dag1* responded more rapidly in Gas (HS0 group) than in TA (HS8 group), whereas the expression levels of *ligand*–*Dag1* pathways did not change much in SOL.

**FIGURE 8:**
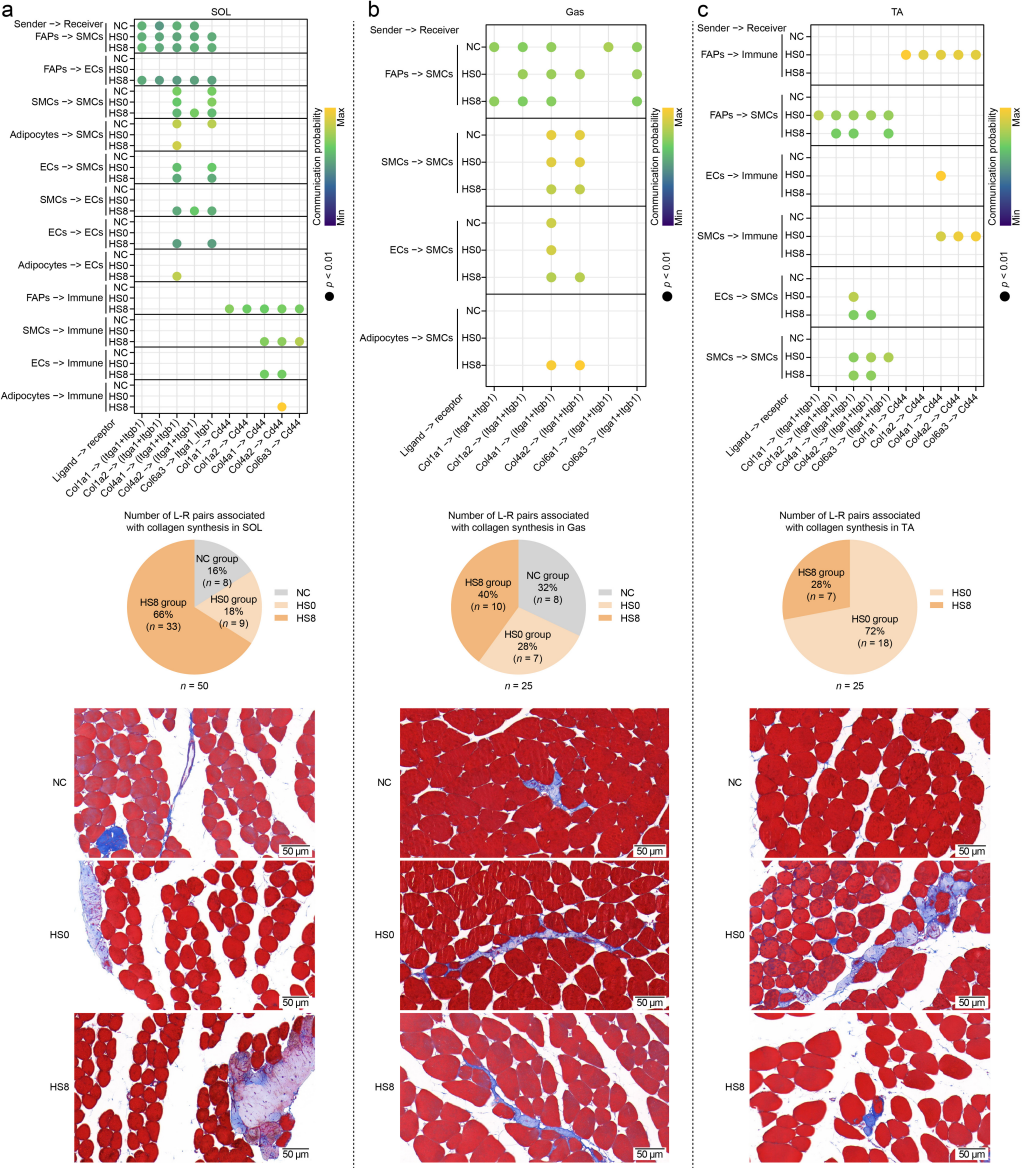
Ligand–receptor pairs related to collagen synthesis in (a) soleus (SOL), (b) gastrocnemius (Gas) and (c) tibialis anterior (TA). The bubble plots showing all significant ligand–receptor pairs associated with collagen synthesis. The pie charts displaying the proportion of ligand–receptor pairs related to collagen synthesis. Masson's trichrome staining indicating collagen deposition (blue).

We observed increased CCI signals associated with collagen synthesis in SOL and TA of HS groups, including the collagen–Cd44 and collagen–integrin pathways (Figure 8, upper and middle panels). Subsequently, Masson's trichrome staining revealed elevated collagen deposition in heat‐stressed SOL and TA (Figure 8, lower panel). These data suggested that the collagen–Cd44 and collagen–integrin pathways may contribute to HS‐induced collagen accumulation in skeletal muscles.

## 5 Discussion

Skeletal muscles showed various changes after HS. Mitochondrial swelling and fusion are closely associated with excessive oxidative stress in skeletal muscles [33]. In heat‐exposed skeletal muscles, lipid droplets act as antioxidant organelles that control polyunsaturated fatty acid storage in triglycerides to reduce membrane lipid peroxidation, preserve organelle function and prevent cell death [34]. Cells adapt and protect themselves by digesting oxidatively damaged proteins and organelles via autophagy [35]. At the tissue level, we detected apoptosis in skeletal muscles subjected to HS. Peak apoptosis occurred in different groups across SOL, Gas and TA, suggesting that the response mechanisms to HS are diverse across anatomical positions. Few heat‐exposed skeletal muscles have been profiled at the bulk RNA‐seq and single‐nucleus levels or across anatomical locations. To address this, we generated the first version of the transcriptomic atlas to facilitate further exploration.

At the tissue level, the transcription profile of the same skeletal muscle subjected to different treatments was smaller than the profile across anatomical positions within the same treatment. This difference could mainly result from different myonuclei composition of the three skeletal muscles [S48]. Euclidean distance, DEG proportions and STEM modules suggested that for mice subjected to an approximately 3‐h heat exposure, transcript dynamics of skeletal muscles were essentially eliminated at 16 h postexposure (HS16 group). In addition, we further observed an interesting phenomenon that the peak of HS‐induced transcriptional changes did not necessarily correspond to the peak of tissue injury. For instance, SOL and Gas in HS0 group exhibited the greatest transcriptional differences compared with SOL and Gas in NC group, whereas SOL and Gas in HS8 group showed more severe tissue damage. We speculated that there were two underlying reasons of this temporal dissociation. On one hand, some immune‐related genes and pathways were activated during the 8‐h recovery period (from NC to HS8 group) (Figure S9). On the other hand, the sequential progression of gene expression is the temporal dissociation between transcriptomic adjustments and phenotypic outcomes [36, 37].

For the first time, we constructed a single‐nucleus atlas with 134 320 high‐quality nuclei from heat‐exposed skeletal muscles. This atlas consists of seven cell types. From myonuclei, the most abundant cell type in skeletal muscles, we identified the largest number of DEGs in SOL and Gas during heat treatment and after 8 h of recovery, which implied that myonuclei were extraordinarily heat sensitive. In HS0 group, we identified two heat‐sensitive populations of type IIb myonuclei (type IIb_2 and IIb_3 myonuclei). Notably, a distinct heat‐sensitive population of type IIa/IIx_2 myonuclei was detected in the HS8 group. This population exhibited high expression of 27 IEGs. However, no heat‐sensitive type I myonuclei were observed in either the HS0 or HS8 groups. This suggested that type IIb myonuclei had the highest sensitivity, followed by type IIa myonuclei, and type I myonuclei were the least sensitive. This phenomenon may be related to antioxidative stress and contraction force. Slow muscle fibres are more tolerant to oxidative stress than fast muscle fibres [38]. Malak et al. [18] demonstrated that high temperature (41.5°C ± 0.5°C) significantly reduces the contraction force of fast fatigue and antifatigue motion units but has no significant effect on slow motion units. In addition, the binding of the IEGs *Nr4a3*, *Fos* and *Cebpd* to the *Junb* gene via different motif sites constitutes a critical pathway for the differentiation of type IIa/IIx_1 myonuclei into the type IIa/IIx_2 myonuclei. Gene networks regulated by two TFs, *Klf13* and *Bcl6*, were critical pathways that mediated the stress response in type IIb_2 and type IIb_3 myonuclei, respectively.

Following heat‐induced myofibre damage, quiescent MuSCs are activated for repair. Activated MuSCs were more abundant in HS (HS0 and HS8) groups than in NC. In contrast, activated MuSCs from HS0 and HS8 exhibited higher differentiation than those from NC. For instance, activated MuSCs of heat‐damaged SOL highly expressed key differentiation genes and TF regulons, including Egr1_extended and Cebpb regulons. Notably, metabolism was reprogrammed in activated MuSCs after HS. To resist HS, activated MuSCs secreted multiple anti‐HS substances (tryptophan [S34], glutathione [S35], d‐glutamine and d‐glutamate [S36], citrate cycle [S37] and arginine [S38]). In summary, the antistress substance metabolic pathway and cell development‐related genes are activated in activated MuSCs subjected to HS.

HS disrupts the cellular caloric balance maintained via the Igf1–Igf1r pathway [S49]. Thermally injured skeletal muscles repair themselves and maintain homeostasis through cell‐to‐cell crosstalk among multiple cell types. The variation in CCI numbers across groups was greater between myonuclei and other cell types than between other cell types. The expression patterns of CCIs that perform the same function differed among the three skeletal muscles, resulting in multiple phenotypes. For example, proliferation signals (Egf and Fgf) were inhibited in SOL, activated in Gas and were not detected in TA after HS (HS0 and HS8 groups). In addition, the collagen content in the extracellular matrix of SOL and TA was upregulated after thermal injury. The signals driving this process may be collagen–Cd44 and collagen–integrin pathways.

## 6 Conclusions

Our study provided the first single‐nucleus resolution atlas of heat‐stressed skeletal muscle, revealing distinct molecular and cellular responses that govern injury and repair. We demonstrated that HS (41.5°C ± 0.5°C, ~3 h) triggered rapid ultrastructural alterations and detected a transcriptomic recovery (Bulk RNA‐seq) within 16 h postexposure. At single‐nucleus resolution, we found that myofibres were the most heat‐sensitive cell type. Notably, myonuclear sensitivity exhibited a hierarchy, with type IIb > IIa/IIx > I nuclei.

## Funding

This study was supported by National Key R&D Program of China (2021YFA0805903, 2022YFD1301200 and 2020YFA0509500), the National Natural Science Foundation of China (32341050, 32272837, 32421005 and 32225046), the Program for Pig Industry Technology System Innovation Team of Sichuan Province (SCCXTD‐2024‐08‐16), the Sichuan Science and Technology Program (2021ZDZX0008, 2021YFYZ0009 and 2021YFYZ0030) and the Strategic Cooperation Funding Project Between Chongqing Municipal Government and Chinese Academy of Agricultural Sciences (23310).

## Conflicts of Interest

The authors declare no conflicts of interest.

## Supporting information


**Data S1:** Supporting Information.

**Data S2:** Supporting Information.

**Table S1:** Known marker genes used for cell annotation.
**Table S2:** The list of immediate early genes (IEGs).
**Table S3:** Rectal temperature of mice before, during and after heat exposure (°C).
**Table S4:** Body weight of mice before, during and after heat exposure (g).
**Table S5:** Cross‐sectional area (CSA) of each myofibre in TA based on HE staining (μm^2^).
**Table S6:** Cross‐sectional area (CSA) of each myofibre in SOL based on HE staining (μm^2^).
**Table S7:** Cross‐sectional area (CSA) of each myofibre in Gas based on HE staining (μm^2^).
**Table S8:** Cell apoptosis rate determination based on TUNEL staining.
**Table S9:** Pairwise Spearman correlation analysis of biological replicates (Rep).
**Table S10:** The nuclei number of the seven cell types in each group.
**Table S11:** The proportion of nuclei number of the seven cell types in each group.
**Table S12:** The differentially expressed immediate early genes (IEGs) identified in HS0/HS8 versus NC groups.
**Table S13:**. The differentially expressed genes (DEGs) identified in regulons of myonuclei collected in SOL.
**Table S14:**. The differentially expressed genes (DEGs) identified in regulons of myonuclei collected in Gas.
**Table S15:**. The differentially expressed genes (DEGs) identified in regulons of myonuclei collected in TA.
**Table S16:** The number of myonuclei in each group.
**Table S17:** The overlap of immediate early genes (IEGs) and DEGs between type IIa/IIx_1 and type IIa/IIx_2 myonuclei.
**Table S18:** Specific expression regulons in type IIa/IIx_2 myonuclei associated with cell differentiation and immediate early response (IER).
**Table S19:** The number of muscle stem cells (MuSCs) in each group.
**Table S20:** Driver genes in differentiation from quiescent to activated MuSCs.
**Table S21:** The coefficient of variation (CV) of proportion of CCIs number in each group.

**Data S4:** Supporting Information.

**Figure S1:** Representative photomicrographs of (a) soleus (SOL) and (b) gastrocnemius (Gas) stained with haematoxylin and eosin (HE). The black arrows indicated myolysis (middle panel). Cross‐sectional area (CSA) of TA based on HE staining (right panel). The values of ‘*n*’ represented the total number of myofibres for cross‐sectional area measurements. Myofibres were derived from three biological replicates, each with two technical replicates (*n* = 6). The CSA of each myofibre was shown in Tables S6 and S7.

**Figure S2:** Cell annotation in the atlas of skeletal muscles. (a) The number of genes and unique molecular identifier (UMI) counts per high‐quality nucleus. (b) Individual gene uniform manifold approximation and projection (UMAP) plots indicating the expression levels and distribution of known marker genes of myonuclei, fibroadipogenic progenitors (FAPs), endothelial cells (ECs), smooth muscle cells (SMCs), muscle stem cells (MuSCs), immune cells and adipocytes. (c,d) The identification of cell type–specific modules using high‐dimensional weighted gene coexpression network analysis (hdWGCNA). (c) UMAP plot showing the expression distribution of hub genes for each module across the seven cell types. (d) The average expression of module‐specific hub genes in different cell types. (e) The function description of hub genes in each cell type.

**Figure S3:** Differential gene expression of six cell types in each skeletal muscle (SOL, Gas and TA). Upset plots represent overlapping differentially expressed genes (DEGs) during (a) heat treatment (NC vs. HS0 group) and (b) 8 h of recovery from heat (HS8 vs. HS0 group).

**Figure S4:** Immediate early genes (IEGs) and transcriptional factor (TF) regulon. (a) The distribution of differentially expressed IEGs in muscle stem cells (MuSCs), fibroadipogenic progenitors (FAPs), endothelial cells (ECs), smooth muscle cells (SMCs) and immune cells. (b) Motifs in the promoter regions of DEGs identified from Gas and TA binding by Mef2c.

**Figure S5:** A single‐nucleus atlas for the mice myonuclei. (a) Individual gene uniform manifold approximation and projection (UMAP) plots indicating the expression levels and distribution of known marker genes for type I myonuclei, type IIa/IIx myonuclei, type IIb myonuclei, myotendinous junction (MTJ) and neuromuscular junction (NMJ). (b) The distribution of myonuclei in each group. (c) Functional analysis of the high variable genes (HVGs) in type IIa and IIb myonulei subtypes. (d) The 27 upregulated immediate early genes in type IIa/IIx_2 myonuclei compared with type IIa/IIx_2 myonuclei.

**Figure S6:** Immunohistochemistry or immunofluorescence of type IIa/IIx_2 and IIb_2 myofibres. (a) The mRNA and protein expression of *Stat3*, a type IIa/IIx_2 myonuclei‐specific marker, in SOL. (b) The distribution of type IIa/IIx_2 myonuclei in SOL. (c) The proportion of type IIa/IIx_2 myofibre/myonuclei identified by immunofluorescence, immunohistochemistry and snRNA‐seq. (d) The mRNA and protein expression of *Hsp70*, a type IIb_2 myonuclei‐specific marker, in Gas. (e) The distribution of type IIb_2 myonuclei in Gas. (f) The proportion of type IIb_2 myofibre/myonuclei identified by immunofluorescence and snRNA‐seq. In Figure S5, immunohistochemistry and immunofluorescence assays were based on two biological replicates with 5–11 technical replicates.

**Figure S7:** Muscle stem cells (MuSCs) participate in tissue repair after heat treatment. (a) The biological function of hypervariable genes (HVGs) in each subtype of MuSCs. (b) The velocity and cell entropy of MuSCs. (c) Differentiation trajectory (left panel) and driver genes (middle panel) from quiescent to activated MuSCs in Gas. Activated MuSCs were predominantly localised to State 5 (Cell Fate 1) and State 6 (Cell Fate 2). GO terms and KEGG pathway analyses of the two states of activated MuSCs (right panel). (d) GO terms and KEGG pathway analyses of high variable genes identified in activated MuSCs of SOL (upper panel) and Gas (lower panel) of HS8 group.

**Figure S8:** The number of activated MuSCs in TA. (a) UMAP projection of 3278 cells from the tibialis anterior (TA) (left panel). Dot plot displays expression levels of marker genes across TA cell types (right panel; Santos et al.). (b) Cell composition of the TA (Santos et al.). (c) UMAP visualisation of 98 MuSC subtypes in the TA (left panel). Dot plot shows marker gene expression patterns among MuSC subtypes (right panel; Santos et al.). (d) Proportion of activated MuSCs relative to total MuSCs/all cells in TA (left panel: data from Santos et al.; right panel: present study).

**Figure S9:** The expression of genes, GO terms and KEGG pathways related to immune response in (a) SOL and (b) Gas HS0 and HS8 groups.
