# Supplementary material for: Single‐Nucleus Transcriptome Reveals Cellular Heterogeneity and Transcriptional Response to Heat Stress in Skeletal Muscle
Source: J Cachexia Sarcopenia Muscle. 2026 Feb 12;17(1):e70217. doi: 10.1002/jcsm.70217 (PMC12895210; doi:10.1002/jcsm.70217)
Supplement: Supplementary file 1 — Data S1: Supporting Information. [file JCSM-17-e70217-s001.docx]

Supplemental Methods

***Histological observation***

Cell apoptosis was detected using TUNEL cell apoptosis detection kit (Servicebio, China). In detail, sections were treated with the environmentally friendly dewaxing agent and transparent agent to expose the skeletal muscles. For antigen retrieval, we add protease K working solution (20 μg/mL) to cover the tissue, and incubate at 37°C for 22 mins. After discarding the protease K working solution, phosphate buffer saline (PBS) buffer was used to wash tissues for 3 times. Subsequently, the permeabilize working solution was applied for the enhancement of cell membrane permeability, and the tissues was washed with PBS. To mark the apoptotic nuclei, the staining solution (volume ratio was 1 TDT enzyme: 5 dUTP: 50 buffers in the kit) was added to covering skeletal muscles at 37°C for 1 h. The cell nuclei in physiological condition were marked by 4′,6-diamidino-2-phenylindole (DAPI) solution. After washing the DAPI solution, coverslips were mounted onto slides with ProLong Gold mountant (Thermo Fisher Scientific, USA). Sections were imaged with florescent microscopy at two excitation wavelengths (330–380 nm and 465–490 nm).

Anti-Ttn (Abcam, USA) and anti-Stat3 (Abways, China) antibodies were used to mark type IIa/IIx_2 myofibers. The IIb_2 myofibers were marked by anti-Hsp70 antibody (HUABIO, China).

To observe heat-stress induced change at the subcellular level, skeletal muscles that preserved in TEM fixative were fixed with 2.5% glutaraldehyde. Then, the tissues refixed with 1% osmium tetroxide, and dehydrated with acetone ^1,2^. Dehydrated samples were successively infiltrated with a 3:1, 1:1, and 1:3 dehydrating agent–Epon812 mixture. These samples were embedded with pure Epon812 and cut into ultrathin slices (60-90 nm), and then shifted to copper grids. The slices were stained first with uranyl acetate (10-15 mins) and next with lead citrate stain (1-2 mins). The ultrathin sections of skeletal muscles were analyzed in a JEM-1400Flash transmission electron microscope (JEOL, Japan).

***Bulk RNA-seq analysis of heat-exposed skeletal muscle***

The downstream analysis of bulk RNA-seq analysis included the principal component analysis (PCA), Euclidean distance, Spearman correlation coefficient and short time-series expression miner (STEM) analysis. At first, the top three principal components were displayed on a multidimensional scatterplot using the R scatterplot3d function ^3^. We calculated Euclidean distance among all groups. In addition, three hallmark gene sets, including oxidative stress, unfolded protein response and myogenesis genes, were downloaded from the Gene Set Enrichment Analysis (GSEA) database (v4.3.3). Based on the expression of gene sets, we calculated Spearman correlation coefficient of each skeletal muscle among the five groups. Furthermore, STEM analysis was introduced to reveal the time-dependent dynamically changed mRNAs ^4^. We explored the differences of molecular mechanisms of SOL, Gas and TA in response to heat stress based on the functions of DEGs. All the GO enrichment and KEGG pathway analysis of the DEGs were realized through Metascape database ^5^.

***Data availability***

Sequencing data have been deposited in the GEO database (https://account.ncbi.nlm.nih.gov) under accession code PRJNA1171600 and PRJNA1183997. The 84 bulk RNA-seq data (all groups) and 12 snRNA-seq data (HS0 and HS8 group) were deposited in NCBI database with the BioProject ID PRJNA1171600. The other six snRNA-seq data (NC group) were download from NCBI database with the BioProject ID PRJNA1183997.

**References of** **supplemental methods**

1 Wan, T. *et al.* Astrocytic phagocytosis contributes to demyelination after focal cortical ischemia in mice. *Nature communications* **13**, 1134, doi:10.1038/s41467-022-28777-9 (2022).

2 Wang, X. *et al.* Treating cutaneous squamous cell carcinoma using 5-aminolevulinic acid polylactic-co-glycolic acid nanoparticle-mediated photodynamic therapy in a mouse model. *International journal of nanomedicine* **10**, 347-355, doi:10.2147/ijn.S71245 (2015).

3 Sheng, J. *et al.* Fate mapping analysis reveals a novel murine dermal migratory Langerhans-like cell population. *eLife* **10**, doi:10.7554/eLife.65412 (2021).

4 Wang, X. *et al.* A MicroRNA-Based Network Provides Potential Predictive Signatures and Reveals the Crucial Role of PI3K/AKT Signaling for Hepatic Lineage Maturation. *Frontiers in cell and developmental biology* **9**, 670059, doi:10.3389/fcell.2021.670059 (2021).

5 Zhou, Y. *et al.* Metascape provides a biologist-oriented resource for the analysis of systems-level datasets. *Nature communications* **10**, 1523, doi:10.1038/s41467-019-09234-6 (2019).
