# Supplementary material for: Single‐Nucleus Transcriptome Reveals Cellular Heterogeneity and Transcriptional Response to Heat Stress in Skeletal Muscle
Source: J Cachexia Sarcopenia Muscle. 2026 Feb 12;17(1):e70217. doi: 10.1002/jcsm.70217 (PMC12895210; doi:10.1002/jcsm.70217)
Supplement: Supplementary file 2 — Data S2: Supporting Information. [file JCSM-17-e70217-s004.docx]

Supplemental References

S1 Leong, N. L., Greskovich, K., Blommer, J. & Jiang, J. CCN1 expression is regulated by mechanical stimuli in tendons. Biochemical and biophysical research communications 663, 25-31, doi:10.1016/j.bbrc.2023.04.058 (2023).

S2 Kumar, P. V. et al. Muscle Transcriptome Sequencing Revealed Thermal Stress-Responsive Regulatory Genes in Farmed Rohu, Labeo rohita (Hamilton, 1822). Marine biotechnology (New York, N.Y.) 25, 1057-1075, doi:10.1007/s10126-023-10259-8 (2023).

S3 Maimaiti, Y. et al. FOXO regulation of TXNIP induces ferroptosis in satellite cells by inhibiting glutathione metabolism, promoting Sarcopenia. Cellular and molecular life sciences : CMLS 82, 81, doi:10.1007/s00018-025-05592-1 (2025).

S4 Lala-Tabbert, N., AlSudais, H., Marchildon, F., Fu, D. & Wiper-Bergeron, N. CCAAT/enhancer binding protein β is required for satellite cell self-renewal. Skeletal muscle 6, 40, doi:10.1186/s13395-016-0112-8 (2016).

S5 Yu X, Cao J, Wang B, et al. Silencing PIM1 inhibits ENO1-induced AKT activation and attenuates fibrillogenesis during spinal cord injury-induced skeletal muscle atrophy. J Biol Chem. 2025;301(8):110398. doi:10.1016/j.jbc.2025.110398.

S6 Gellhaus, B. et al. Foxo3 Knockdown Mediates Decline of Myod1 and Myog Reducing Myoblast Conversion to Myotubes. Cells 12, doi:10.3390/cells12172167 (2023).

S7 Salyers, Z. R., Coleman, M., Le, D. & Ryan, T. E. AAV-mediated expression of PFKFB3 in myofibers, but not endothelial cells, improves ischemic muscle function in mice with critical limb ischemia. American journal of physiology. Heart and circulatory physiology 323, H424-h436, doi:10.1152/ajpheart.00121.2022 (2022).

S8 Sandaradura, S. A. et al. Nemaline myopathy and distal arthrogryposis associated with an autosomal recessive TNNT3 splice variant. Human mutation 39, 383-388, doi:10.1002/humu.23385 (2018).

S9 Alessio E, Buson L, Chemello F, et al. Single cell analysis reveals the involvement of the long non-coding RNA Pvt1 in the modulation of muscle atrophy and mitochondrial network. *Nucleic Acids Res*. 2019;47(4):1653-1670. doi:10.1093/nar/gkz007.

S10 Garton FC, Houweling PJ, Vukcevic D, et al. The Effect of ACTN3 Gene Doping on Skeletal Muscle Performance. *Am J Hum Genet*. 2018;102(5):845-857. doi:10.1016/j.ajhg.2018.03.009.

S11 Michelucci A, Paolini C, Canato M, et al. Antioxidants protect calsequestrin-1 knockout mice from halothane- and heat-induced sudden death. *Anesthesiology*. 2015;123(3):603-617. doi:10.1097/ALN.0000000000000748.

S12 Zhang T, Wang T, Niu Q, et al. Transcriptional atlas analysis from multiple tissues reveals the expression specificity patterns in beef cattle. *BMC Biol*. 2022;20(1):79. Published 2022 Mar 29. doi:10.1186/s12915-022-01269-4.

S13 Cheng X, DeGiorgio M. Flexible Mixture Model Approaches That Accommodate Footprint Size Variability for Robust Detection of Balancing Selection. *Mol Biol Evol*. 2020;37(11):3267-3291. doi:10.1093/molbev/msaa134.

S14 Barefield DY, Puckelwartz MJ, Kim EY, et al. Experimental Modeling Supports a Role for MyBP-HL as a Novel Myofilament Component in Arrhythmia and Dilated Cardiomyopathy. *Circulation*. 2017;136(16):1477-1491. doi:10.1161/CIRCULATIONAHA.117.028585.

S15 Liu Z, Han S, Shen X, et al. The landscape of DNA methylation associated with the transcriptomic network in layers and broilers generates insight into embryonic muscle development in chicken. *Int J Biol Sci*. 2019;15(7):1404-1418. Published 2019 Jun 2. doi:10.7150/ijbs.35073.

S16 Schweighauser M, Shi Y, Murzin AG, et al. Distinct tau filament folds in human MAPT mutants P301L and P301T. *Nat Struct Mol Biol*. Published online May 29, 2025. doi:10.1038/s41594-025-01575-9.

S17 Yang Y, Wu S, Qu R, et al. Mechanical sensor PDLIM5 promotes the osteogenesis of human adipose-derived stem cells through microfilament alterations. *Genes Dis.* 2023;11(3):101023. Published 2023 Jul 11. doi:10.1016/j.gendis.2023.06.001.

S18 Kose S, Imai K, Watanabe A, et al. Lack of Hikeshi activates HSF1 activity under normal conditions and disturbs the heat-shock response. Life Sci Alliance. 2022 May 17;5(9):e202101241. doi: 10.26508/lsa.202101241. PMID: 35580988; PMCID: PMC9113944.

S19 De Maio A, Cauvi DM, Capone R, et al. The small heat shock proteins, HSPB1 and HSPB5, interact differently with lipid membranes. Cell Stress Chaperones. 2019 Sep;24(5):947-956. doi: 10.1007/s12192-019-01021-y. Epub 2019 Jul 23. PMID: 31338686; PMCID: PMC6717221.

S20 Hu L, Fang H, Abbas Z, et al. The HSP90AA1 gene is involved in heat stress responses and its functional genetic polymorphisms are associated with heat tolerance in Holstein cows. *J Dairy Sci*. 2024;107(7):5132-5149. doi:10.3168/jds.2023-24007.

S21 Wang F, Bonam SR, Schall N, et al. Blocking nuclear export of HSPA8 after heat shock stress severely alters cell survival. *Sci Rep*. 2018;8(1):16820. Published 2018 Nov 14. doi:10.1038/s41598-018-34887-6.

S22 Li P, Wang J, Zou Y, et al. Interaction of Hsp90AA1 with phospholipids stabilizes membranes under stress conditions. *Biochim Biophys Acta Biomembr*. 2019;1861(2):457-465. doi:10.1016/j.bbamem.2018.11.009.

S23 Fang C, Yang Z, Shi L, et al. Circulating Sestrin Levels Are Increased in Hypertension Patients. *Dis Markers*. 2020;2020:3787295. Published 2020 Jun 12. doi:10.1155/2020/3787295.

S24 Asadi G, Rezaei Varmaziar F, Karimi M, et al. Determination of the transcriptional level of long non-coding RNA NEAT-1, downstream target microRNAs, and genes targeted by microRNAs in diabetic neuropathy patients. *Immunol Lett*. 2021;232:20-26. doi:10.1016/j.imlet.2021.01.007.

S25 Zhang J, He Z, Xiao W, et al. Overexpression of BAG3 Attenuates Hypoxia-Induced Cardiomyocyte Apoptosis by Inducing Autophagy. *Cell Physiol Biochem*. 2016;39(2):491-500. doi:10.1159/000445641.

S26 Absalón-Aguilar A, Torres-Ruiz JJ, Mejía-Domínguez NR, et al. TRIM63 and Atrogin-1 are key drivers of systemic and muscle inflammation in patients with idiopathic inflammatory myopathies. *Clin Exp Rheumatol*. 2025;43(2):326-333. doi:10.55563/clinexprheumatol/p2lma6.

S27 Chen, Y. et al. Adipocyte IRE1α promotes PGC1α mRNA decay and restrains adaptive thermogenesis. Nature metabolism 4, 1166-1184, doi:10.1038/s42255-022-00631-8 (2022).

S28 Zhao, Y. et al. Neutrophil Membrane-Camouflaged Polyprodrug Nanomedicine for Inflammation Suppression in Ischemic Stroke Therapy. Advanced materials (Deerfield Beach, Fla.) 36, e2311803, doi:10.1002/adma.202311803 (2024).

S29 Liu, X., Xu, C., Xiao, W. & Yan, N. Unravelling the role of NFE2L1 in stress responses and related diseases. Redox biology 65, 102819, doi:10.1016/j.redox.2023.102819 (2023).

S30 Chakrabarty, Y., Yang, Z., Chen, H. & Chan, D. C. The HRI branch of the integrated stress response selectively triggers mitophagy. Molecular cell 84, 1090-1100.e1096, doi:10.1016/j.molcel.2024.01.016 (2024).

S31 Kuang Q, Liang Y, Zhuo Y, et al. The ALDOA Metabolism Pathway as a Potential Target for Regulation of Prostate Cancer Proliferation. *Onco Targets Ther*. 2021;14:3353-3366. Published 2021 May 24. doi:10.2147/OTT.S290284.

S32 Fu Y, Li S, Nie J, et al. Expression of PDLIM5 Spliceosomes and Regulatory Functions on Myogenesis in Pigs. *Cells*. 2024;13(8):720. Published 2024 Apr 21. doi:10.3390/cells13080720.

S33 Kilpeläinen TO, Bentley AR, Noordam R, et al. Multi-ancestry study of blood lipid levels identifies four loci interacting with physical activity. *Nat Commun*. 2019;10(1):376. Published 2019 Jan 22. doi:10.1038/s41467-018-08008-w.

S34 Ouyang, J. et al. Tryptophan alleviates chronic heat stress-induced impairment of antioxidant capacities, inflammatory response, and mitochondrial function in broilers. Tropical animal health and production 55, 425, doi:10.1007/s11250-023-03842-7 (2023).

S35 Song, J. H. et al. Korean Red Ginseng and Korean black ginseng extracts, JP5 and BG1, prevent hepatic oxidative stress and inflammation induced by environmental heat stress. Journal of ginseng research 44, 267-273, doi:10.1016/j.jgr.2018.12.005 (2020).

S36 Sifa, D. et al. Dietary glutamine improves meat quality, skeletal muscle antioxidant capacity and glutamine metabolism in broilers under acute heat stress. Journal of Applied Animal Research 46, 1412-1417, doi:10.1080/09712119.2018.1520113 (2018).

S37 Li, X. S. et al. Citric Acid Confers Broad Antibiotic Tolerance through Alteration of Bacterial Metabolism and Oxidative Stress. International journal of molecular sciences 24, doi:10.3390/ijms24109089 (2023).

S38 Yoon, S. Y. et al. Effects of Zinc Oxide and Arginine on the Intestinal Microbiota and Immune Status of Weaned Pigs Subjected to High Ambient Temperature. Animals : an open access journal from MDPI 10, doi:10.3390/ani10091537 (2020).

S39 Rossi, A. et al. Regulation of Vegf signaling by natural and synthetic ligands. Blood 128, 2359-2366, doi:10.1182/blood-2016-04-711192 (2016).

S40 Dougherty, U. et al. The renin-angiotensin system mediates EGF receptor-vitamin d receptor cross-talk in colitis-associated colon cancer. Clinical cancer research : an official journal of the American Association for Cancer Research 20, 5848-5859, doi:10.1158/1078-0432.Ccr-14-0209 (2014).

S41 Vanarsa, K. et al. Quantitative planar array screen of 1000 proteins uncovers novel urinary protein biomarkers of lupus nephritis. Annals of the rheumatic diseases 79, 1349-1361, doi:10.1136/annrheumdis-2019-216312 (2020).

S42 Asada, K. et al. Uncovering Prognosis-Related Genes and Pathways by Multi-Omics Analysis in Lung Cancer. Biomolecules 10, doi:10.3390/biom10040524 (2020).

S43 Huang, G. et al. Diabetes impairs cardioprotective function of endothelial progenitor cell-derived extracellular vesicles via H3K9Ac inhibition. Theranostics 12, 4415-4430, doi:10.7150/thno.70821 (2022).

S44 Tong, M. et al. Multi-omics landscapes of colorectal cancer subtypes discriminated by an individualized prognostic signature for 5-fluorouracil-based chemotherapy. Oncogenesis 5, e242, doi:10.1038/oncsis.2016.51 (2016).

S45 Horton, E. R. et al. Definition of a consensus integrin adhesome and its dynamics during adhesion complex assembly and disassembly. Nature cell biology 17, 1577-1587, doi:10.1038/ncb3257 (2015).

S46 Beedle, A. M. et al. Mouse fukutin deletion impairs dystroglycan processing and recapitulates muscular dystrophy. The Journal of clinical investigation 122, 3330-3342, doi:10.1172/jci63004 (2012).

S47 Davra, V. et al. Cyclophilin A Inhibitor Debio-025 Targets Crk, Reduces Metastasis, and Induces Tumor Immunogenicity in Breast Cancer. Molecular cancer research : MCR 18, 1189-1201, doi:10.1158/1541-7786.Mcr-19-1144 (2020).

S48 Wang, Q. et al. The hepatokine TSK maintains myofiber integrity and exercise endurance and contributes to muscle regeneration. JCI insight 7, doi:10.1172/jci.insight.154746 (2022).

S49 Soto, M., Cai, W., Konishi, M. & Kahn, C. R. Insulin signaling in the hippocampus and amygdala regulates metabolism and neurobehavior. Proceedings of the National Academy of Sciences of the United States of America 116, 6379-6384, doi:10.1073/pnas.1817391116 (2019).
