# Supplementary material for: Single‐Nucleus Transcriptome Reveals Cellular Heterogeneity and Transcriptional Response to Heat Stress in Skeletal Muscle
Source: J Cachexia Sarcopenia Muscle. 2026 Feb 12;17(1):e70217. doi: 10.1002/jcsm.70217 (PMC12895210; doi:10.1002/jcsm.70217)
Supplement: Supplementary file 3 — Table S1: Known marker genes used for cell annotation. Table S2: The list of immediate early genes (IEGs). Table S3: Rectal temperature of mice before, during and after heat exposure (°C). Table S4: Body weight of mice before, during and after heat exposure (g). Table S5: Cross‐sectional area (CSA) of each myofibre in TA based on HE staining (μm2). Table S6: Cross‐sectional area (CSA) of each myofibre in SOL based on HE staining (μm2). Table S7: Cross‐sectional area (CSA) of each myofibre in Gas based on HE staining (μm2). Table S8: Cell apoptosis rate determination based on TUNEL staining. Table S9: Pairwise Spearman correlation analysis of biological replicates (Rep). Table S10: The nuclei number of the seven cell types in each group. Table S11: The proportion of nuclei number of the seven cell types in each group. Table S12: The differentially expressed immediate early genes (IEGs) identified in HS0/HS8 versus NC groups. Table S13:. The differentially expressed genes (DEGs) identified in regulons of myonuclei collected in SOL. Table S14:. The differentially expressed genes (DEGs) identified in regulons of myonuclei collected in Gas. Table S15:. The differentially expressed genes (DEGs) identified in regulons of myonuclei collected in TA. Table S16: The number of myonuclei in each group. Table S17: The overlap of immediate early genes (IEGs) and DEGs between type IIa/IIx_1 and type IIa/IIx_2 myonuclei. Table S18: Specific expression regulons in type IIa/IIx_2 myonuclei associated with cell differentiation and immediate early response (IER). Table S19: The number of muscle stem cells (MuSCs) in each group. Table S20: Driver genes in differentiation from quiescent to activated MuSCs. Table S21: The coefficient of variation (CV) of proportion of CCIs number in each group. [file JCSM-17-e70217-s005.docx]

Supplemental Table

**Table S1.** Known marker genes used for cell annotation.

| Cell types or subtypes | Maker genes | References of Table S1 |
| --- | --- | --- |
| Myonuclei | *Ttn* | ^1-5^ |
| Muscle stem cells (MuSCs) | *Pax7* | ^1-3,6-8^ |
|  | *Myf5* | ^6,9^ |
| Fibroadipogenic progenitors  (FAPs) | *Dcn* | ^1,8^ |
|  | *Lvrn* | ^1^ |
|  | *Col6a3* | ^1^ |
| Smooth muscle cells (SMCs) | *Myh11* | ^3,5^ |
|  | *Acta2* | ^9,10^ |
|  | *Gucy1b1* | ^11^ |
| Endothelial cells (ECs) | *Pecam1* | ^3,8,9,12^ |
|  | *Flt1* | ^5^ |
|  | *Mecom* | ^5^ |
|  | *Aqp1* | ^6^ |
|  | *Itga6* | ^6^ |
|  | *Cdh5* | ^9,11,12^ |
| Adipocytes | *Adipoq* | ^8,13^ |
|  | *Fasn* | ^14^ |
|  | *Lipe* | ^14^ |
|  | *Lgals12* | ^14^ |
| Immune cells | *Ptprc* | ^8-10^ |
|  | *Cd163* | ^14^ |
|  | *Addgr1* | ^14^ |
| Type I myonuclei | *Myh7* | ^1,2,4,8^ |
| Type IIa/IIx_1 myonuclei | *Myh2* | ^15^ |
|  | *Myh1* | ^15^ |
| Type IIa/IIx_2 myonuclei | *Flnc, Tacc2, Usp28, Lmcd1, Nrap, Pfkfb4* | ^16^ |
| Type IIb myonuclei | *Myh4* | ^2,10^ |
| Myotendinous junction  (MTJ) | *Nav3* | ^1^ |
|  | *Col22a1* | ^1-3,17^ |
|  | *Tigd4* | ^4,17^ |
| Neuromuscular junction  (NMJ) | *Etv5* | ^1,17^ |
|  | *Chrne* | ^2,3,17^ |
|  | *Ache* | ^17^ |
|  | *Ano4* | ^17^ |
| Quiescent MuSCs | *Cd34* | ^18^ |
|  | *Spry1* | ^11,19,20^ |
|  | *Myf5* | ^18^ |
|  | *Chrdl2* | ^20^ |
|  | *Cav1* | ^20^ |
| Activated MuSCs | *Tpm1* | ^21^ |
|  | *Tnnt3* | ^22^ |
|  | *Egr1* | ^21^ |
|  | *Des* | ^21^ |
|  | *Tpm2* | ^22^ |
| Proliferative MuSCs | *Smc4* | ^22^ |
|  | *Ezh2* | ^22^ |
|  | *Mki67* | ^21,22^ |
|  | *Top2a* | ^21,22^ |
|  | *Cenpf* | ^21,22^ |
|  | *Ccnb2* | ^22,23^ |
| Myoblasts | *Myog* | ^7,23,24^ |
|  | *Myod1* | ^23,24^ |
|  | *Mymk* | ^5,23^ |
|  | *Tnnt2* | ^7^ |
|  | *Cdkn1a* | ^7,23^ |
|  | *Cdkn1c* | ^7^ |
|  | *Dll1* | ^23^ |

**Table S2.** The list of immediate early genes (IEGs).

| IEGs | IEGs | IEGs | IEGs | IEGs | IEGs | IEGs | IEGs | IEGs | IEGs |
| --- | --- | --- | --- | --- | --- | --- | --- | --- | --- |
| *Ccl3* | *Id2* | *Tsc22d1* | *Hbegf* | *Tlr2* | *Txnip* | *Nfkbid* | *Inhba* | *Socs3* | *Ifit3* |
| *Slc2a3* | *Btg2* | *Peli1* | *Pmaip1* | *F3* | *Ptgs2* | *Icam1* | *Ikbke* | *Bcl3* | *Ackr4* |
| *Ier3* | *Nfib* | *Acod1* | *Fosl1* | *Ccn1* | *Trib1* | *Serpine1* | *Maff* | *Ier2* | *Apold1* |
| *Fosb* | *Klf6* | *Mbnl2* | *Ccn2* | *Gem* | *Csrnp1* | *Klf10* | *Ccrl2* | *Homer1* | *Flg* |
| *Il12b* | *Arhgef3* | *Myc* | *Il23a* | *Gbp2* | *Egr3* | *Egr2* | *Zfp36* | *Per2* | *Cxcl12* |
| *Sod2* | *Klf2* | *Hes1* | *Il6* | *Nr4a3* | *Ifit1* | *Vcam1* | *Zfp36l2* | *Cebpb* | *Ccl7* |
| *Gadd45b* | *Sgk1* | *Arc* | *Rgs1* | *Rheb* | *Dusp5* | *Egr1* | *Npas4* | *Ier5* | *Saa2* |
| *Cd83* | *Per1* | *Rcan1* | *Rgs2* | *Fosl2* | *Cxcl10* | *Gdf15* | *Ifit2* | *Cxcl2* | *Saa1* |
| *Srf* | *Nfkbia* | *Nr4a1* | *Atf3* | *Cxcl1* | *Ccl5* | *Mcl1* | *Marcksl1* | *Nptx2* | *Ifit1b* |
| *Il10* | *Zfp36l1* | *Noct* | *Nr4a2* | *Bhlhe40* | *Fbxo33* | *Nrn1* | *Bdnf* | *Cxcl11* | *Ccl18* |
| *Irf1* | *Fos* | *Pim1* | *Traf1* | *Clec4e* | *Ccl12* | *Ncoa7* | *Ifnb1* | *Arih1* | *Ccnl1* |
| *Csf2* | *Gadd45g* | *Sik1* | *Dusp2* | *Cd69* | *Nfkbiz* | *Saa3* | *Rasd1* | *Crem* | *Cxcl3* |
| *Tnfaip3* | *Plk2* | *Dusp1* | *Il1b* | *Wee1* | *Ccl2* | *Thbs1* | *Mmp13* | *Jund* | *Gbp1* |
| *Dusp6* | *Plau* | *Map3k8* | *Il1a* | *Plat* | *Tnfsf9* | *Gbp2b* | *Jun* | *Egr4* | *Klhl21* |
| *Nup98* | *Arf4* | *Tnf* | *Pias1* | *Ldlr* | *Pcdh8* | *Ppp1r15a* | *Junb* | *Cebpd* | *Nr2c2* |

Note: The 150 IEGs were download from the studies of Wu et al. ^25^ and Tullai et al ^26^.

**Table S3.** Rectal temperature of mice before, during and after heat-exposure (℃).

| Replication | 0 min  (NC group) | 60 min | 90 min | 180 min  (HS0 group) |
| --- | --- | --- | --- | --- |
| 1 | 37.5 | 38.7 | 38.3 | 41.1 |
| 2 | 37.7 | 37.8 | 38.0 | 41.8 |
| 3 | 37.8 | 37.6 | 39.4 | 41.4 |
| 4 | 37.5 | 36.9 | 38.4 | 41.2 |
| 5 | 37.8 | 37.1 | 38.7 | - |
| 6 | 37.2 | 37.2 | 38.8 | - |
| 7 | 37.3 | 37.4 | 38.8 | - |
| 8 | 37.5 | 38.3 | 37.9 | - |
| 9 | 37.9 | 38.8 | 38.9 | - |

**Table S4.** Body weight of mice before, during and after heat-exposure (g).

| Replication | 0 min  (NC group) | 60 min | 90 min | 180 min  (HS0 group) |
| --- | --- | --- | --- | --- |
| 1 | 19.8 | 19.2 | 19.1 | 18.2 |
| 2 | 20.7 | 20.1 | 19.9 | 17.5 |
| 3 | 19.7 | 19.6 | 18.9 | 18.8 |
| 4 | 20.4 | 19.0 | 19.6 | 17.9 |
| 5 | 19.7 | 20.1 | 18.8 | 17.9 |
| 6 | 20.4 | 19.9 | 19.6 | 18.5 |
| 7 | 20.4 | 18.1 | 19.7 | 18.2 |
| 8 | 18.3 | 20.9 | 17.9 | 18.6 |
| 9 | 21.2 | 19.2 | 20.6 | 18.6 |

**Table S5.** Cross-sectional area (CSA) of each myofiber in TA based on HE staining (µm^2^).

| NC | NC | HS0 | HS0 | HS0 |
| --- | --- | --- | --- | --- |
| 3481.834 | 2052.336 | 2709.775 | 3395.329 | 2733.564 |
| 2949.827 | 3445.069 | 3581.315 | 4894.031 | 1762.543 |
| 1146.194 | 3492.647 | 1948.530 | 4688.582 | 2835.208 |
| 2577.855 | 6135.381 | 2993.080 | 5352.509 | 3821.367 |
| 2571.367 | 6578.720 | 4522.059 | 2218.858 | 2640.571 |
| 3765.139 | 5705.018 | 2262.111 | 3775.952 | 1879.325 |
| 3287.197 | 1881.488 | 3317.474 | 2216.696 | 1600.346 |
| 3583.478 | 1814.446 | 2357.267 | 1868.512 | 4643.167 |
| 1381.920 | 2383.218 | 1911.765 | 3096.886 | 1743.080 |
| 2971.453 | 6148.356 | 2802.768 | 1760.381 | 4463.668 |
| 2850.346 | 1658.737 | 2677.336 | 4312.284 | 1087.803 |
| 3425.606 | 3161.765 | 3602.941 | 2169.118 | 2009.083 |
| 2344.291 | 6120.243 | 3062.284 | 2311.851 | 5080.018 |
| 2716.263 | 949.395 | 2407.007 | 3070.934 | 3224.481 |
| 3481.834 | 1195.934 | 4753.460 | 1944.204 | 1665.225 |
| 5028.114 | 4054.931 | 5121.107 | 891.004 | 2545.415 |
| 2564.879 | 2763.841 | 2019.896 | 2683.824 | 3438.582 |
| 3553.201 | 2089.100 | 2404.844 | 2339.966 | 1939.879 |
| 2809.256 | 6686.852 | 1890.138 | 2303.201 | 1442.474 |
| 3198.530 | 3280.709 | 1256.488 | 1038.062 | 3157.439 |
| 6055.364 | 1820.934 | 3006.055 | 1717.128 | 1578.720 |
| 4839.966 | 4597.751 | 2848.184 | 1541.955 | 2588.668 |
| 3280.709 | 1898.789 | 2973.616 | 2443.772 | 2097.751 |
| 2028.547 | 2865.485 | 1970.156 | 2493.512 | 1524.654 |
| 3367.215 | 2255.623 | 3747.837 | 5179.499 | 4059.256 |
| 1567.907 | 1602.509 | 2173.443 | 2082.613 | 1371.107 |
| 2102.076 | 3561.851 | 5570.935 | 4855.104 | 4279.844 |
| 6526.817 |  | 2595.156 | 2802.768 | 2906.574 |
| 1833.910 |  | 2361.592 | 3933.824 | 1963.668 |
| 2530.277 |  | 1665.225 | 4937.284 | 2528.114 |
| 5460.640 |  | 4826.990 | 2130.190 | 3057.958 |
| 1929.066 |  | 2320.502 | 1630.623 | 1544.118 |
| 1695.502 |  | 2288.062 | 2830.883 | 2128.028 |

**Table S6.** Cross-sectional area (CSA) of each myofiber in SOL based on HE staining (µm^2^).

| NC | NC | NC | NC | HS0 | HS0 | HS0 | HS0 |
| --- | --- | --- | --- | --- | --- | --- | --- |
| 789.417 | 713.931 | 842.737 | 660.179 | 695.904 | 581.524 | 738.763 | 708.680 |
| 665.261 | 947.517 | 700.446 | 501.492 | 474.334 | 680.638 | 940.666 | 1029.964 |
| 716.566 | 653.326 | 1007.037 | 1255.929 | 322.122 | 703.315 | 550.773 | 953.746 |
| 467.636 | 864.747 | 958.677 | 761.529 | 677.098 | 703.868 | 280.262 | 634.364 |
| 800.422 | 731.601 | 686.651 | 1158.370 | 604.090 | 55.309 | 780.143 | 539.477 |
| 790.657 | 1043.307 | 754.852 | 1032.691 | 613.493 | 576.813 | 489.655 | 823.900 |
| 626.201 | 917.757 | 870.017 | 662.135 | 623.670 | 590.963 | 499.643 | 565.636 |
| 1110.267 | 632.556 | 955.112 | 668.370 | 492.918 | 697.860 | 540.904 | 645.660 |
| 798.562 | 823.362 | 673.786 | 667.881 | 733.957 | 338.526 | 943.995 | 746.017 |
| 790.192 | 940.232 | 698.586 | 552.594 | 639.046 | 682.640 | 1009.512 | 769.560 |
| 772.677 | 799.337 | 1174.437 | 705.780 | 1031.411 | 532.342 | 369.441 | 710.226 |
| 1063.922 | 992.467 | 803.057 | 771.676 | 803.204 | 276.338 | 720.690 | 789.774 |
| 944.882 | 544.361 | 826.307 | 707.125 | 706.744 | 415.933 | 676.932 | 466.231 |
| 673.166 | 583.421 | 1008.742 | 809.086 | 827.208 | 695.600 | 575.505 | 636.385 |
| 615.816 | 669.136 | 921.167 | 1277.324 | 744.355 | 1219.738 | 851.130 | 523.424 |
| 544.361 | 1157.387 | 855.912 | 643.185 | 639.709 | 642.806 | 784.185 | 779.191 |
| 1005.177 | 637.671 | 640.461 | 603.942 | 735.063 | 553.389 | 707.491 | 537.337 |
| 1212.722 | 905.512 | 799.492 | 555.284 | 629.422 | 561.356 | 597.384 | 811.177 |
| 559.086 | 668.516 | 629.766 | 774.977 | 781.744 | 958.264 | 371.344 | 717.241 |
| 857.927 | 943.797 | 957.902 | 606.998 | 1279.861 | 896.552 | 726.992 | 463.496 |
| 962.862 | 965.497 | 703.236 | 674.972 | 907.518 | 475.981 | 561.593 | 647.681 |
| 704.941 | 936.512 | 628.216 | 971.808 | 595.351 | 670.036 | 684.304 | 644.471 |
| 644.801 | 1076.167 | 689.751 | 853.831 | 1001.323 | 333.056 | 604.518 | 532.342 |
| 639.531 | 677.196 | 868.777 | 475.818 | 756.523 | 392.628 | 443.639 | 510.820 |
| 780.427 | 830.027 | 589.271 | 741.601 | 689.709 | 784.780 | 575.030 | 720.214 |
| 964.257 | 699.206 | 753.338 |  | 371.458 | 962.426 | 434.483 | 701.427 |
| 742.916 | 657.666 | 984.645 |  | 620.019 | 754.221 | 774.435 | 797.265 |
| 884.587 | 730.671 | 1179.153 |  | 765.040 | 783.948 | 726.992 | 902.497 |
| 265.826 | 777.482 | 879.383 |  | 163.605 | 602.140 | 924.257 | 573.008 |
| 929.382 | 948.137 | 810.920 |  | 644.687 | 725.565 | 922.354 | 514.863 |
| 846.922 | 633.796 | 780.356 |  | 780.306 | 753.389 | 517.479 | 497.146 |
| 495.071 | 1016.337 | 1051.518 |  | 322.233 | 654.221 | 668.609 | 741.736 |
| 750.047 | 638.291 | 877.060 |  | 767.917 | 604.518 | 438.288 | 722.949 |
| 177.010 | 1134.602 | 837.205 |  | 638.603 | 914.269 | 728.537 | 652.794 |
| 1141.732 | 992.777 | 1067.778 |  | 716.479 | 405.826 | 684.304 | 879.310 |
| 682.931 | 1018.507 | 929.630 |  | 776.102 | 569.084 | 739.596 | 416.528 |
| 854.052 | 630.076 | 887.207 |  | 1024.442 | 413.912 | 730.916 | 491.082 |
| 700.291 | 841.497 | 708.470 |  | 566.922 | 814.031 | 570.036 | 596.790 |
| 331.081 | 920.702 | 823.268 |  | 603.758 | 856.480 | 737.812 | 634.483 |

**Table S7.** Cross-sectional area (CSA) of each myofiber in Gas based on HE staining (µm^2^).

| NC | NC | NC | HS0 | HS0 | HS0 | HS0 |
| --- | --- | --- | --- | --- | --- | --- |
| 2289.180 | 3754.697 | 1838.843 | 1121.802 | 1698.759 | 1507.759 | 1555.250 |
| 1761.712 | 1408.086 | 1364.003 | 1567.570 | 1681.714 | 1494.823 | 1541.033 |
| 2219.144 | 1111.415 | 1387.297 | 1027.139 | 1292.865 | 559.912 | 1525.879 |
| 2044.590 | 134.245 | 1604.161 | 1181.157 | 1514.760 | 641.486 | 1422.300 |
| 1261.474 | 565.874 | 1856.397 | 1356.177 | 504.514 | 1315.542 | 1474.793 |
| 2012.604 | 786.445 | 1100.299 | 1612.315 | 1144.631 | 1155.284 | 1471.668 |
| 1646.492 | 1671.106 | 1637.947 | 930.650 | 1507.607 | 962.306 | 1396.835 |
| 1313.317 | 1675.505 | 1780.043 | 1708.347 | 435.876 | 1576.672 | 1628.990 |
| 440.904 | 1560.048 | 1017.237 | 853.641 | 1807.120 | 1154.628 | 1435.111 |
| 1588.228 | 1096.908 | 1578.669 | 1812.903 | 1212.204 | 945.379 | 1806.777 |
| 1780.499 | 1115.696 | 1947.509 | 1442.013 | 1706.521 | 1615.259 | 1437.454 |
| 1804.281 | 993.222 | 1369.123 | 893.363 | 1195.006 | 1605.045 | 1273.415 |
| 1802.378 | 874.673 | 1645.509 | 1732.089 | 1662.538 | 1814.293 | 1229.202 |
| 1139.477 | 789.536 | 1465.236 | 1571.832 | 705.863 | 1275.637 | 1628.365 |
| 1670.868 | 2053.401 | 1305.210 | 1819.295 | 1556.460 | 1217.331 | 1681.482 |
| 2078.240 | 1995.208 | 1688.687 | 1509.433 | 605.569 | 1793.298 | 1560.874 |
| 1961.593 | 2049.978 | 1929.823 | 704.646 | 813.310 | 1801.809 | 1125.623 |
| 1255.410 | 1185.633 | 969.912 | 1684.606 | 1172.938 | 1204.280 | 1633.364 |
| 1966.944 | 1601.423 | 947.226 | 1666.647 | 1463.015 | 1650.015 | 1056.414 |
| 1563.971 | 1784.073 | 1080.418 | 1019.530 | 1241.729 | 1455.804 | 1960.974 |
| 1658.621 | 1377.940 | 1896.769 | 525.669 | 1278.407 | 1167.112 | 1626.178 |
| 1723.306 | 1714.632 | 2015.203 | 986.961 | 462.053 | 1186.973 | 1675.702 |
| 1094.055 | 1781.261 | 1518.294 | 1634.839 | 1242.642 | 1301.740 | 1842.866 |
| 2072.652 | 1995.330 | 838.306 | 972.655 | 1491.323 | 1741.659 | 1657.579 |
| 457.194 | 2090.689 | 429.094 | 1525.261 | 1478.691 | 1550.995 | 1553.688 |
| 2038.763 | 1754.976 | 1424.254 | 1421.619 | 657.771 | 1674.700 | 1017.982 |
| 1202.021 | 258.815 | 1757.235 | 1814.577 | 245.789 | 1406.861 | 1487.760 |
| 1330.797 | 1800.822 | 1619.529 | 1619.011 | 1343.697 | 1462.614 | 1498.539 |
| 894.887 | 1741.528 | 1702.958 | 1016.942 | 1485.844 | 1494.675 | 857.848 |
| 1489.655 | 1993.374 | 1279.231 | 499.796 | 1384.484 | 1461.053 | 1258.417 |
| 1016.647 | 1782.972 | 1065.050 | 1732.241 | 620.027 | 1522.054 | 1290.756 |
| 1613.436 | 2183.481 | 1714.911 | 1365.460 | 938.259 | 1799.965 | 1382.618 |
| 1457.313 | 920.338 | 1707.836 | 742.998 | 1475.342 | 1459.209 | 1598.369 |
| 1747.681 | 1502.885 | 1232.394 | 1091.516 | 1532.414 | 1437.504 | 1662.110 |
| 812.723 | 1585.530 | 1361.195 | 763.544 | 1339.131 | 1623.345 | 1508.850 |
| 1128.537 | 1264.976 | 1462.553 | 1789.618 | 573.000 | 1516.664 | 1503.070 |
| 1237.693 | 1777.104 |  | 901.277 | 990.918 | 1788.616 | 1201.394 |
| 1451.367 | 1712.064 |  | 359.628 | 1311.889 | 1428.850 | 1054.852 |
| 905.707 | 1855.103 |  | 1070.057 | 1113.736 | 1424.175 |  |
| 835.553 | 1458.507 |  | 1166.699 | 667.663 | 1479.323 |  |

**Table S8.** Cell apoptosis rate determination based on TUNEL staining.

| Group | Replicates | DAPI^+^  nuclei | TUNEL^+^  nuclei | Total nuclei  per sample | Total nuclei per group | TUNEL^+^ nuclei per sample (%) | TUNEL^+^ nuclei (Mean±SD, %) |
| --- | --- | --- | --- | --- | --- | --- | --- |
| NC_SOL | 1 | 77 | 0 | 77 | 189 | 0.00 | 0.00 |
|  | 2 | 52 | 0 | 52 |  | 0.00 |  |
|  | 3 | 60 | 0 | 60 |  | 0.00 |  |
| HS0_SOL | 1 | 54 | 13 | 67 | 181 | 19.40 | 17.58±1.48 |
|  | 2 | 47 | 10 | 57 |  | 17.54 |  |
|  | 3 | 48 | 9 | 57 |  | 15.79 |  |
| HS8_SOL | 1 | 63 | 25 | 88 | 269 | 28.41 | 27.88±0.47 |
|  | 2 | 67 | 26 | 93 |  | 27.96 |  |
|  | 3 | 64 | 24 | 88 |  | 27.27 |  |
| HS16_SOL | 1 | 73 | 10 | 83 | 254 | 12.05 | 13.76±1.24 |
|  | 2 | 72 | 12 | 84 |  | 14.29 |  |
|  | 3 | 74 | 13 | 87 |  | 14.94 |  |
| HS24_SOL | 1 | 64 | 8 | 72 | 185 | 11.11 | 10.85±0.87 |
|  | 2 | 56 | 6 | 62 |  | 9.68 |  |
|  | 3 | 45 | 6 | 51 |  | 11.76 |  |
| NC_Gas | 1 | 31 | 0 | 31 | 108 | 0.00 | 0.00 |
|  | 2 | 49 | 0 | 49 |  | 0.00 |  |
|  | 3 | 28 | 0 | 28 |  | 0.00 |  |
| HS0_Gas | 1 | 42 | 9 | 51 | 148 | 17.65 | 14.80±2.07 |
|  | 2 | 43 | 7 | 50 |  | 14.00 |  |
|  | 3 | 41 | 6 | 47 |  | 12.77 |  |
| HS8_Gas | 1 | 51 | 17 | 68 | 237 | 25.00 | 25.03±1.82 |
|  | 2 | 71 | 21 | 92 |  | 22.83 |  |
|  | 3 | 56 | 21 | 77 |  | 27.27 |  |
| HS16_Gas | 1 | 61 | 6 | 67 | 136 | 8.96 | 12.07±3.68 |
|  | 2 | 36 | 4 | 40 |  | 10.00 |  |
|  | 3 | 24 | 5 | 29 |  | 17.24 |  |
| HS24_Gas | 1 | 34 | 4 | 38 | 159 | 10.53 | 7.23±2.79 |
|  | 2 | 52 | 2 | 54 |  | 3.70 |  |
|  | 3 | 62 | 5 | 67 |  | 7.46 |  |
| NC_TA | 1 | 31 | 0 | 31 | 108 | 0.00 | 0.00 |
|  | 2 | 49 | 0 | 49 |  | 0.00 |  |
|  | 3 | 28 | 0 | 28 |  | 0.00 |  |
| HS0_TA | 1 | 90 | 118 | 208 | 534 | 56.73 | 49.33±6.34 |
|  | 2 | 94 | 66 | 160 |  | 41.25 |  |
|  | 3 | 83 | 83 | 166 |  | 50.00 |  |
| HS8_TA | 1 | 73 | 38 | 111 | 343 | 34.23 | 34.71±1.58 |
|  | 2 | 79 | 39 | 118 |  | 33.05 |  |
|  | 3 | 72 | 42 | 114 |  | 36.84 |  |
| HS16_TA | 1 | 58 | 8 | 66 | 224 | 12.12 | 10.84±0.97 |
|  | 2 | 59 | 7 | 66 |  | 10.61 |  |
|  | 3 | 83 | 9 | 92 |  | 9.78 |  |
| HS24_TA | 1 | 63 | 3 | 66 | 218 | 4.55 | 6.75±1.59 |
|  | 2 | 78 | 7 | 85 |  | 8.24 |  |
|  | 3 | 62 | 5 | 67 |  | 7.46 |  |

**Table S9.** Pairwise Spearman correlation analysis of biological replicates (Rep).

|  | Rep 1 | Rep 2 | Rep 3 | Rep 4 | Rep 5 | Rep 6 | Rep 1 | Rep 2 | Rep 3 | Rep 4 | Rep 5 | Rep 6 |
| --- | --- | --- | --- | --- | --- | --- | --- | --- | --- | --- | --- | --- |
| Group | SOL_NC |  |  |  |  |  | Gas_HS16 |  |  |  |  |  |
| Rep 1 | 1.00 | 0.99 | 0.99 | 0.98 | 0.98 | - | 1.00 | 0.99 | 0.99 | 0.98 | 0.98 | 0.98 |
| Rep 2 | 0.99 | 1.00 | 0.99 | 0.98 | 0.98 | - | 0.99 | 1.00 | 0.99 | 0.98 | 0.98 | 0.99 |
| Rep 3 | 0.99 | 0.99 | 1.00 | 0.98 | 0.98 | - | 0.99 | 0.99 | 1.00 | 0.98 | 0.98 | 0.98 |
| Rep 4 | 0.98 | 0.98 | 0.98 | 1.00 | 0.99 | - | 0.98 | 0.98 | 0.98 | 1.00 | 0.98 | 0.99 |
| Rep 5 | 0.98 | 0.98 | 0.98 | 0.99 | 1.00 | - | 0.98 | 0.98 | 0.98 | 0.98 | 1.00 | 0.99 |
| Rep 6 | - | - | - | - | - | - | 0.98 | 0.99 | 0.98 | 0.99 | 0.99 | 1.00 |
| Group | SOL_HS0 |  |  |  |  |  | Gas_HS24 |  |  |  |  |  |
| Rep 1 | 1.00 | 0.98 | 0.99 | 0.98 | 0.97 | 0.97 | 1.00 | 0.99 | 0.99 | 0.98 | 0.98 | 0.99 |
| Rep 2 | 0.98 | 1.00 | 0.98 | 0.97 | 0.97 | 0.97 | 0.99 | 1.00 | 0.99 | 0.98 | 0.98 | 0.98 |
| Rep 3 | 0.99 | 0.98 | 1.00 | 0.98 | 0.97 | 0.97 | 0.99 | 0.99 | 1.00 | 0.98 | 0.98 | 0.98 |
| Rep 4 | 0.98 | 0.97 | 0.98 | 1.00 | 0.98 | 0.99 | 0.98 | 0.98 | 0.98 | 1.00 | 0.99 | 0.99 |
| Rep 5 | 0.97 | 0.97 | 0.97 | 0.98 | 1.00 | 0.98 | 0.98 | 0.98 | 0.98 | 0.99 | 1.00 | 0.99 |
| Rep 6 | 0.97 | 0.97 | 0.97 | 0.99 | 0.98 | 1.00 | 0.99 | 0.98 | 0.98 | 0.99 | 0.99 | 1.00 |
| Group | SOL_HS8 |  |  |  |  |  | TA_NC |  |  |  |  |  |
| Rep 1 | 1.00 | 0.99 | 0.98 | 0.97 | 0.97 | - | 1.00 | 0.99 | 0.99 | 0.98 | 0.98 | 0.98 |
| Rep 2 | 0.99 | 1.00 | 0.99 | 0.97 | 0.98 | - | 0.99 | 1.00 | 0.99 | 0.98 | 0.98 | 0.98 |
| Rep 3 | 0.98 | 0.99 | 1.00 | 0.98 | 0.98 | - | 0.99 | 0.99 | 1.00 | 0.98 | 0.98 | 0.98 |
| Rep 4 | 0.97 | 0.97 | 0.98 | 1.00 | 0.98 | - | 0.98 | 0.98 | 0.98 | 1.00 | 0.99 | 0.98 |
| Rep 5 | 0.97 | 0.98 | 0.98 | 0.98 | 1.00 | - | 0.98 | 0.98 | 0.98 | 0.99 | 1.00 | 0.99 |
| Rep 6 | - | - | - | - | - | - | 0.98 | 0.98 | 0.98 | 0.98 | 0.99 | 1.00 |
| Group | SOL_HS16 |  |  |  |  |  | TA_HS0 |  |  |  |  |  |
| Rep 1 | 1.00 | 0.99 | 0.99 | 0.98 | 0.98 | - | 1.00 | 0.99 | 0.99 | 0.98 | 0.98 | - |
| Rep 2 | 0.99 | 1.00 | 0.99 | 0.98 | 0.98 | - | 0.99 | 1.00 | 0.99 | 0.97 | 0.97 | - |
| Rep 3 | 0.99 | 0.99 | 1.00 | 0.98 | 0.98 | - | 0.99 | 0.99 | 1.00 | 0.98 | 0.97 | - |
| Rep 4 | 0.98 | 0.98 | 0.98 | 1.00 | 0.99 | - | 0.98 | 0.97 | 0.98 | 1.00 | 0.98 | - |
| Rep 5 | 0.98 | 0.98 | 0.98 | 0.99 | 1.00 | - | 0.98 | 0.97 | 0.97 | 0.98 | 1.00 | - |
| Group | SOL_HS24 |  |  |  |  |  | TA_HS8 |  |  |  |  |  |
| Rep 1 | 1.00 | 0.99 | 0.99 | 0.98 | 0.98 | 0.98 | 1.00 | 0.99 | 0.99 | 0.98 | 0.97 | 0.97 |
| Rep 2 | 0.99 | 1.00 | 0.99 | 0.98 | 0.98 | 0.98 | 0.99 | 1.00 | 0.99 | 0.97 | 0.97 | 0.97 |
| Rep 3 | 0.99 | 0.99 | 1.00 | 0.98 | 0.98 | 0.98 | 0.99 | 0.99 | 1.00 | 0.97 | 0.98 | 0.97 |
| Rep 4 | 0.98 | 0.98 | 0.98 | 1.00 | 0.99 | 0.99 | 0.98 | 0.97 | 0.97 | 1.00 | 0.98 | 0.98 |
| Rep 5 | 0.98 | 0.98 | 0.98 | 0.99 | 1.00 | 0.98 | 0.97 | 0.97 | 0.98 | 0.98 | 1.00 | 0.99 |
| Rep 6 | 0.98 | 0.98 | 0.98 | 0.99 | 0.98 | 1.00 | 0.97 | 0.97 | 0.97 | 0.98 | 0.99 | 1.00 |
| Group | Gas_NC |  |  |  |  |  | TA_HS16 |  |  |  |  |  |
| Rep 1 | 1.00 | 0.99 | 0.99 | 0.98 | 0.98 | 0.98 | 1.00 | 0.99 | 0.99 | 0.98 | 0.98 | 0.98 |
| Rep 2 | 0.99 | 1.00 | 0.99 | 0.98 | 0.99 | 0.98 | 0.99 | 1.00 | 0.99 | 0.98 | 0.98 | 0.98 |
| Rep 3 | 0.99 | 0.99 | 1.00 | 0.98 | 0.99 | 0.98 | 0.99 | 0.99 | 1.00 | 0.98 | 0.98 | 0.98 |
| Rep 4 | 0.98 | 0.98 | 0.98 | 1.00 | 0.99 | 0.99 | 0.98 | 0.98 | 0.98 | 1.00 | 0.98 | 0.99 |
| Rep 5 | 0.98 | 0.99 | 0.99 | 0.99 | 1.00 | 0.99 | 0.98 | 0.98 | 0.98 | 0.98 | 1.00 | 0.99 |
| Rep 6 | 0.98 | 0.98 | 0.98 | 0.99 | 0.99 | 1.00 | 0.98 | 0.98 | 0.98 | 0.99 | 0.99 | 1.00 |
| Group | Gas_HS0 |  |  |  |  |  | TA_HS24 |  |  |  |  |  |
| Rep 1 | 1.00 | 0.98 | 0.99 | 0.98 | 0.97 | 0.97 | 1.00 | 0.99 | 0.99 | 0.98 | 0.98 | - |
| Rep 2 | 0.98 | 1.00 | 0.98 | 0.97 | 0.97 | 0.96 | 0.99 | 1.00 | 0.99 | 0.98 | 0.98 | - |
| Rep 3 | 0.99 | 0.98 | 1.00 | 0.98 | 0.98 | 0.97 | 0.99 | 0.99 | 1.00 | 0.98 | 0.98 | - |
| Rep 4 | 0.98 | 0.97 | 0.98 | 1.00 | 0.98 | 0.98 | 0.98 | 0.98 | 0.98 | 1.00 | 0.99 | - |
| Rep 5 | 0.97 | 0.97 | 0.98 | 0.98 | 1.00 | 0.97 | 0.98 | 0.98 | 0.98 | 0.99 | 1.00 | - |
| Rep 6 | 0.97 | 0.96 | 0.97 | 0.98 | 0.97 | 1.00 | - | - | - | - | - | - |
| Group | Gas_HS8 |  |  |  |  |  | - | - | - | - | - | - |
| Rep 1 | 1.00 | 0.99 | 0.99 | 0.98 | 0.98 | - | - | - | - | - | - | - |
| Rep 2 | 0.99 | 1.00 | 0.99 | 0.98 | 0.98 | - | - | - | - | - | - | - |
| Rep 3 | 0.99 | 0.99 | 1.00 | 0.98 | 0.98 | - | - | - | - | - | - | - |
| Rep 4 | 0.98 | 0.98 | 0.98 | 1.00 | 0.98 | - | - | - | - | - | - | - |
| Rep 5 | 0.98 | 0.98 | 0.98 | 0.98 | 1.00 | - | - | - | - | - | - | - |

**Table S10.** The nuclei number of the seven cell types in each group.

|  | NC_SOL | HS0_SOL | HS8_SOL | NC_Gas | HS0_Gas | HS8_Gas | NC_TA | HS0_TA | HS8_TA | Total |
| --- | --- | --- | --- | --- | --- | --- | --- | --- | --- | --- |
| Myonuclei | 6,713 | 6,032 | 6,073 | 10,622 | 9,937 | 11,518 | 11,197 | 11,652 | 12,593 | 86,337 |
| MuSCs | 612 | 559 | 396 | 540 | 308 | 664 | 246 | 145 | 348 | 3,818 |
| FAPs | 4,180 | 5,515 | 2,614 | 4,805 | 2,284 | 3,193 | 1,062 | 474 | 1,082 | 25,209 |
| SMCs | 798 | 439 | 530 | 407 | 278 | 558 | 224 | 298 | 551 | 4,083 |
| ECs | 2,835 | 2,114 | 2,233 | 1,167 | 669 | 858 | 723 | 233 | 919 | 11,751 |
| Adipocytes | 94 | 68 | 52 | 96 | 105 | 138 | 20 | 2 | 8 | 583 |
| Immune cells | 482 | 552 | 208 | 541 | 276 | 320 | 86 | 30 | 44 | 2,539 |
| Total | 15,714 | 15,279 | 12,106 | 18,178 | 13,857 | 17,249 | 13,558 | 12,834 | 15,545 | 134,320 |

**Table S11.** The proportion of nuclei number of the seven cell types in each group.

|  | NC_SOL | HS0_SOL | HS8_SOL | NC_Gas | HS0_Gas | HS8_Gas | NC_TA | HS0_TA | HS8_TA |
| --- | --- | --- | --- | --- | --- | --- | --- | --- | --- |
| Myonuclei | 42.72% | 39.48% | 50.17% | 58.43% | 71.71% | 66.77% | 82.59% | 90.79% | 81.01% |
| MuSCs | 3.89% | 3.66% | 3.27% | 2.97% | 2.22% | 3.85% | 1.81% | 1.13% | 2.24% |
| FAPs | 26.60% | 36.10% | 21.59% | 26.43% | 16.48% | 18.51% | 7.83% | 3.69% | 6.96% |
| SMCs | 5.08% | 2.87% | 4.38% | 2.24% | 2.01% | 3.23% | 1.65% | 2.32% | 3.54% |
| ECs | 18.04% | 13.84% | 18.45% | 6.42% | 4.83% | 4.97% | 5.33% | 1.82% | 5.91% |
| Adipocytes | 0.60% | 0.45% | 0.43% | 0.53% | 0.76% | 0.80% | 0.15% | 0.02% | 0.05% |
| Immune cells | 3.07% | 3.61% | 1.72% | 2.98% | 1.99% | 1.86% | 0.63% | 0.23% | 0.28% |
| Total | 100.00% | 100.00% | 100.00% | 100.00% | 100.00% | 100.00% | 100.00% | 100.00% | 100.00% |

**Table S12.** The differentially expressed immediate early genes (IEGs) identified in HS0/HS8 versus NC groups.

| Group | Direction | DEGs | Tissue |
| --- | --- | --- | --- |
| NC vs. HS0 | Up in HS0 | *Ccn1* (Myonuclei), *Cebpd* (MuSCs, FAPs), *F3* (FAPs), Ccn2 (SMCs), *Serpine1* (SMCs), *Sgk1* (ECs)*, Klf6* (ECs)*, Txnip* (ECs, Immune) | SOL |
|  |  | *Homer1* (Myonuclei), *Pim1* (Myonuclei), *Txnip* (MuSCs), *Nfib* (MuSCs), *Dusp1* (FAPs, ECs), *Zfp36l1* (SMCs), *Klf6* (SMCs), *Mbnl2* (SMCs, ECs), *Sgk1* (ECs), *Per1* (Immune) | Gas |
|  |  | *Txnip* (MuSCs, SMCs, ECs), *Vcam1* (MuSCs), *Ccnl1* (MuSCs, FAPs, SMCs, ECs, Immune), *Ccn2* (FAPs), *Klf6* (SMCs, Immune), *Sgk1* (ECs), *Dusp1* (ECs), *Mbnl2* (ECs), *Ldlr* (Immune), *Nfkbia* (Immune), *Nfkbiz* (Immune), *Ncoa7* (Immune), *Id2* (Immune), *Mcl1* (Immune), *Zfp36l2* (Immune), *Nup98* (Immune), *Map3k8* (Immune) | TA |
|  | Down in HS0 | *Per2* (MuSCs, FAPs, SMCs) | SOL |
|  |  | *Hes1* (MuSCs, SMCs)*, Cxcl12* (ECs) | Gas |
|  |  | *Sgk1* (Myonuclei)*, Mbnl2* (Myonuclei, FAPs, Immune) | TA |
| NC vs. HS8 | Up in HS8 | *Ccn2* (SMCs) | SOL |
|  |  | *Ldlr* (MuSCs), *Mbnl2* (SMCs), *Klf2* (ECs), *Dusp1* (ECs), *Txnip* (Immune) | Gas |
|  |  | *Ldlr* (MuSCs), *Txnip* (FAPs, SMCs, Immune), *Mbnl2* (ECs), *Ccnl1* (Immune), *Klf6* (Immune) | TA |
|  | Down in HS8 | *Per2* (MuSCs, FAPs, SMCs, ECs), *Per1* (FAPs, ECs) | SOL |
|  |  | *Per2* (SMCs), *Hes1* (SMCs), *Arf4* (Immune) | Gas |

**Table S13.** The differentially expressed genes (DEGs) identified in regulons of myonuclei collected in SOL.

| Group | Direction | DEGs in regulons |
| --- | --- | --- |
| SOL  NC vs. HS0 | Up in HS0 | Mef2c (286g)  Motif 1 (scertf__spivak.RLM1, 10 kb): *Myo18a, Sgcd, Sorbs1, Tacc2*  Motif 2 (cisbp__M6341, 10 kb): *Acsl1, Filip1, Rps6ka3, Spata1, Atp2c1*  Motif 3 (jaspar__MA0369.1, 10 kb): *Fbxo40*  Motif 4 (transfac_pro__M04758, 10 kb): *Usp28*  Motif 5 (dbcorrdb__MEF2C__ENCSR000BNG_1__m1, 10 kb): *Pdlim3*  Motif 6 (taipale_cyt_meth__MEF2C_CCWWATWWRG_FL_meth_repr, 10 kb): *Pdk4*  Motif 7 (cisbp__M4467, 500 bp): *Myl1, Pdlim5, Slc4a4, Smpx, Xirp2*  Motif 8 (hocomoco__MEF2C_HUMAN.H11MO.0.A, 500 bp): *Fhl1*  Motif 9 (homer__DCYAAAAATAGM_Mef2c, 500 bp): *Asph*  Motif 10 (transfac_pro__M04758, 500 bp): *Slc8a1* |
|  |  | Ppara (24g)  Motif 1 (hocomoco__PPARA_HUMAN.H11MO.0.B, 10 kb): *Gbe1*, *Myom3*  Motif 2 (transfac_pro__M00763, 10 kb): *Lmcd1* |
|  | Down in HS0 | Esrrg (28g): *Tnk2* (cisbp__M5412, 10 kb) |
|  |  | Tef_extended (16g): *Tef* (transfac_public__M00228, 10 kb) |
|  |  | Mlxipl_extended (16g): *Tnk2* (yetfasco__YOR344C_397, 10 kb) |
|  |  | Pax7_extended (44g)  Motif 1 (hocomoco__PAX3_HUMAN.H11MO.0.D, 10 kb): *Rora*  Motif 2 (hocomoco__PAX3_MOUSE.H11MO.0.D, 500 bp): *Tgfbr3* |
| SOL  NC vs. HS8 | Up in HS8 | Tcf3_extended (13g): *Clic5* (transfac_public__M00066, 10 kb) |
|  |  | Ppara (24g)  Motif 1 (hocomoco__PPARA_HUMAN.H11MO.0.B, 10 kb): *Esrrg, Myom3*  Motif 2 (transfac_pro__M00763, 10 kb): *Actn2, Lmcd1* |
|  | Down in HS8 | Mlxipl_extended (16g): *Tnk2* (yetfasco__YOR344C_397, 10 kb) |
|  |  | Pax7_extended (44g): *Rora* (hocomoco__PAX3_HUMAN.H11MO.0.D, 10 kb) |
|  |  | Tef_extended (16g): *Tef* (transfac_public__M00228, 10 kb) |
|  |  | Esrrg (28g): *Tnk2, Filip1l, Itgb6* (cisbp__M5412, 10 kb) |
|  |  | Mef2c (286g)  Motif 1 (scertf__spivak.RLM1, 500 bp): *Camk2g*  Motif 2 (yetfasco__YPL089C_419, 10 kb): *Pfkfb1, Rhobtb1*  Motif 3 (scertf__spivak.RLM1, 10 kb): *Atp2a1, Klhl33, Ky, Mylk2, Pfkm, Tbx15*  Motif 4 (transfac_pro__M00941, 500 bp): *Fbxo32*  Motif 5 (cisbp__M6341, 10 kb): *Ghr, Osbpl9, Tmtc1*  Motif 6 (swissregulon__hs__MEF2_A_B_C_D_.p2, 10 kb): *Gpcpd1, Neat1*  Motif 7 (stark__GNCTANWWATA, 10 kb): *Cdc14a*  Motif 8 (transfac_pro__M07326, 10 kb): *Tnk2*  Motif 8 (cisbp__M4467, 10 kb): *Sec14l5*  Motif 9 (dbcorrdb__MEF2C__ENCSR000BNG_1__m1, 10 kb): Mlf1, Rilpl1  Motif 10 (scertf__morozov.SMP1, 10 kb): *Map2k6*  Motif 11 (taipale_cyt_meth__MEF2C_CCWWATWWRG_FL_meth_repr, 10 kb): *Eepd1*  Motif 12 (cisbp__M4467, 500 bp): *Art1, Kcnn2, Sema6c, Tcea3, Tnnt3* |

**Table S14.** The differentially expressed genes (DEGs) identified in regulons of myonuclei collected in Gas.

| Group | Direction | DEGs in regulons |
| --- | --- | --- |
| Gas  NC vs. HS0 | Up in HS0 | Cebpd_extended (13g): *Ppp1r3c* (cisbp__M5318, 10 kb) |
|  |  | Irf2_extended (13g): *Cbfb* (dbcorrdb__MTA3__ENCSR000BRH_1__m1, 10 kb) |
|  |  | Pax7_extended (44g)  Motif 1 (hocomoco__PAX3_HUMAN.H11MO.0.D, 10 kb): *Adcy2, Zhx2*  Motif 2 (hocomoco__PAX3_MOUSE.H11MO.0.D, 500 bp): *Tgfbr3, Nedd4l* |
|  |  | Tef_extended (16g)  Motif 1 (hocomoco__TEF_HUMAN.H11MO.0.D, 10 kb): *Zhx2*  Motif 2 (stark__TTAYGTAA, 10 kb): *Anxa11* |
|  |  | Foxp2 (24g)  Motif 1 (cisbp__M0751, 10 kb): *Foxp2*  Motif 2 (cisbp__M0750, 500 bp): *Nedd4l* |
|  |  | Mef2c (286g)  Motif 1 (scertf__spivak.RLM1, 10 kb): *Golga4, Ikzf2, Sgcd, Tacc2*  Motif 2 (transfac_pro__M00941, 500 bp): *Fbxo32*  Motif 3 (cisbp__M6341, 10 kb): *Filip1, Snta1, Spata1*  Motif 4 (swissregulon__hs__MEF2_A_B_C_D_.p2, 10 kb): *Pdzrn3*  Motif 5 (cisbp__M4467, 10 kb): *Atp2c1*  Motif 6 (transfac_pro__M04758, 10 kb): *Rbm20*  Motif 7 (dbcorrdb__MEF2C__ENCSR000BNG_1__m1, 10 kb): *Mapt*  Motif 8 (taipale_cyt_meth__MEF2C_CCWWATWWRG_FL_meth_repr, 10 kb): *Pdk4*  Motif 9 (cisbp__M4467, 500 bp): *Atxn1, Homer1, Ldb3, Pdlim5, Slc4a4, Sox6*  Motif 10 (homer__DCYAAAAATAGM_Mef2c, 500 bp): *Asph*  Motif 11 (transfac_pro__M04758, 500 bp): *Dmd, Slc8a1* |
|  |  | Nfia (21g): *Zbtb20* (jaspar__MA0670.1, 10 kb) |
|  | Down in HS0 | Cebpb_extended (21g): *Atp5b, Pvalb* (dbcorrdb__CREB1__ENCSR000BRB_1__m1, 500 bp) |
|  |  | Tcf3_extended (13g): *Srsf5* (taipale_cyt_meth__BHLHA15_NMCATATGKN_eDBD_meth, 10 kb) |
|  |  | Ppara (24g)  Motif 1 (hocomoco__PPARA_HUMAN.H11MO.0.B, 10 kb): *Tpm2*  Motif 2 (transfac_pro__M00763, 10 kb): *Myh2* |
| Gas  NC vs. HS8 | Up in HS8 | Cebpd_extended (13g): *Ppp1r3c* (cisbp__M5318, 10 kb) |
|  |  | Nfia (21g): *Nfia* (jaspar__MA0670.1, 10 kb) |
|  | Down in HS8 | Esrrg (28g): *Mybpc1* (cisbp__M5412, 10 kb) |
|  |  | Mef2c (286g)  Motif 1 (yetfasco__YPL089C_419, 500 bp): *Rhobtb1*  Motif 2 (cisbp__M4675, 10 kb): *Hk2*  Motif 3 (cisbp__M4467, 500 bp): *Mat2a, Myl1*  Motif 4 (hocomoco__MEF2C_HUMAN.H11MO.0.A, 500 bp): *Myoz1* |
|  |  | Ppara (24g): *Myh2* (transfac_pro__M00763, 10 kb) |

**Table S15.** The differentially expressed genes (DEGs) identified in regulons of myonuclei collected in TA.

| Group | Direction | DEGs in regulons |
| --- | --- | --- |
| TA  NC vs. HS0 | Up in HS0 | Cebpd_extended (13g): *Dnajb4*  (taipale_tf_pairs__TEAD4_CEBPD_NTTRCGYAANNNNNNNNRGWATGY_CAP_repr, 10 kb) |
|  |  | Mga_extended (14g): *Cstf3, Mga*  (taipale_tf_pairs__MGA_DLX3_YNATTANRGGTGTGAN_CAP_repr, 10 kb) |
|  |  | Tef_extended (16g): *Anxa11* (stark__TTAYGTAA, 10 kb) |
|  |  | Foxp2 (24g)  Motif 1 (cisbp__M0751, 10 kb): *Foxp2*  Motif 2 (cisbp__M0750, 500 bp): *Nedd4l* |
|  |  | Ppara (24g)  Motif 1 (hocomoco__PPARA_HUMAN.H11MO.0.B, 10 kb): *Tpm2*  Motif 2 (transfac_pro__M00763, 10 kb): *Myh2* |
|  | Down in HS0 | Cebpb_extended (21g): *Ldha* (dbcorrdb__CREB1__ENCSR000BRB_1__m1, 500 bp) |
|  |  | Foxo3_extended (11g): *Pik3r1* (cisbp__M2261, 10 kb)  Tcf3_extended (13g): *Srsf5* (taipale_cyt_meth__BHLHA15_NMCATATGKN_eDBD_meth, 10 kb)  Zmiz1_extended (28g): Zmiz1 (dbcorrdb__ZMIZ1__ENCSR000EFQ_1__m1, 500 bp)  Esrrg (28g): *Filip1l* (cisbp__M5412, 10 kb)  Mef2a (13g): *Sgk1* (cisbp__M3550, 10 kb) |
|  |  | Mef2c (286g)  Motif 1 (transfac_pro__M07424, 500 bp): *Tfdp2*  Motif 2 (scertf__spivak.RLM1, 10 kb): *Atp2a1, Cmya5, Hs3st5, Klhl33, Pcbp2, Pfkm, Tbx15*  Motif 3 (cisbp__M6341, 10 kb): *Gpd2, Osbpl9, Pde4dip, Tmem233*  Motif 4 (swissregulon__hs__MEF2_A_B_C_D_.p2, 10 kb): *Gpcpd1, Neat1*  Motif 5 (stark__GNCTANWWATA, 10 kb): *Ano5, Cdc14a*  Motif 6 (yetfasco__YPL089C_419, 10 kb): *Foxn3*  Motif 7 (dbcorrdb__MEF2C__ENCSR000BNG_1__m1, 10 kb): *Mlf1*  Motif 8 (taipale_cyt_meth__MEF2C_CCWWATWWRG_FL_meth_repr, 10 kb): *Eepd1*  Motif 9 (cisbp__M4467, 500 kb): *Art1, Casq1, Kbtbd12, Phka1, Slc8a3, Tbc1d1, Tnnt3, Tpm1, Trim55* |
| TA  NC vs. HS8 | Up in HS8 | Ppara (24g): *Tpm2* (hocomoco__PPARA_HUMAN.H11MO.0.B, 10 kb) |
|  | Down in HS8 | Esrrg (28g): *Aldh1a1, Mybpc1* (cisbp__M5412, 10 kb) |
|  |  | Mef2a (13g): *Sgk1* (cisbp__M3550, 10 kb) |
|  |  | Mef2c (286g)  Motif 1 (transfac_pro__M07424, 500 bp): *Tfdp2*  Motif 2 (scertf__spivak.RLM1, 10 kb): *Exoc4, Tacc2, Tbx15, Tob1*  Motif 3 (scertf__morozov.SMP1, 500 bp): *Ppp1r12b*  Motif 4 (stark__GNCTANWWATA, 10 kb): *Cdc14a, Jph1*  Motif 5 (transfac_pro__M01301, 10 kb): *Ube2b*  Motif 6 (taipale_cyt_meth__MEF2B_CCWWATWWRG_eDBD, 500 bp): *Pde4d*  Motif 7 (cisbp__M4467, 500 bp): *Mat2a, Phka1, Ppp1r3a, Tbc1d1, Xirp2*  Motif 8 (hocomoco__MEF2C_HUMAN.H11MO.0.A, 500 bp): *Myoz1* |
|  |  | Nfia (21g): *Zbtb20* (jaspar__MA0670.1, 10 kb) |

**Table S16.** The number of myonuclei in each group.

|  | NC_SOL | HS0_SOL | HS8_SOL | NC_Gas | HS0_Gas | HS8_Gas | NC_TA | HS0_TA | HS8_TA | Total |
| --- | --- | --- | --- | --- | --- | --- | --- | --- | --- | --- |
| Type I myonuclei | 2,376 | 2,178 | 1,756 | 227 | 163 | 184 | 50 | 78 | 104 | 7,116 |
| Type IIa/IIx_1 myonuclei | 3,271 | 3,211 | 3,070 | 2,751 | 2,076 | 3,088 | 4,588 | 4,545 | 5,023 | 31,623 |
| Type IIa/IIx_2 myonuclei | 35 | 52 | 584 | 13 | 23 | 22 | 21 | 3 | 40 | 793 |
| Type IIb_1 myonuclei | 299 | 170 | 147 | 6,508 | 4,325 | 6,569 | 6,225 | 2,066 | 6,901 | 33,210 |
| Type IIb_2 myonuclei | 2 | 12 | 3 | 0 | 1,116 | 3 | 0 | 2,038 | 3 | 3,177 |
| Type IIb_3 myonuclei | 21 | 88 | 21 | 93 | 1,888 | 138 | 94 | 2,363 | 151 | 4,857 |
| Type IIb_4 myonuclei | 415 | 136 | 278 | 834 | 137 | 1,249 | 25 | 421 | 160 | 3,655 |
| MTJ | 223 | 128 | 142 | 156 | 163 | 221 | 163 | 89 | 162 | 1,447 |
| NMJ | 71 | 57 | 72 | 40 | 46 | 44 | 31 | 49 | 49 | 459 |
| Total | 6,713 | 6,032 | 6,073 | 10,622 | 9,937 | 11,518 | 11,197 | 11,652 | 12,593 | 86,337 |

**Table S17.** The overlap of immediate early genes (IEGs) and DEGs between type IIa/IIx_1 and type IIa/IIx_2 myonuclei.

| Gene set | Number | The overlap genes |
| --- | --- | --- |
| Down-regulated IEGs | 4 | *Nfib, Tsc22d1, Dusp1, Homer1* |
| Up-regulated IEGs | 27 | *Gadd45b, Nup98, Btg2, Per1, Fos, Mbnl2, Hes1, Nr4a1, Pim1, Sik1, Atf3, Ccn1, Nr4a3, Bhlhe40, Ldlr, Txnip, Nfkbiz, Serpine1, Mcl1, Ppp1r15a, Zfp36, Bdnf, Junb, Cebpb, Cebpd, Ccnl1, Nr2c2* |

**Table S18.** Specific expression regulons in type IIa/IIx_2 myonuclei associated with cell differentiation and immediate early response (IER).

| Regulon | TFs | Target genes |
| --- | --- | --- |
| Junb (110g)  (Cell differentiation) | Junb | ***Junb, Anxa7, Atp13a3, Atp1a1, Atp2a2, Chmp4b, Fkbp5, Flnc, Klf9, Mafk, Map2k3, Midn, Mt2, Nuak1, Ppp2ca, Ripk1, Runx1, Sat1, Tfdp2, Ubc, Eif4e, Gab2, Gabarapl1, Gna13, Smchd1, Zfp143, Esr1, Hk2, Pfkfb4, Slc10a6, Trafd1, Slc25a25, Tiparp, Adamts1, Rrad, Ampd3, Eef2k, Gbe1, Macf1, Ptpn3, Slc39a14, Syt12, Tbc1d15, Tcp11l2, Tpm3, Tsc22d3, Cnksr1, Aff1, Arhgef12, Bmpr1b, Chd7, Fzd4, Hspb1, Met, Mt1, Musk, Pde4b, Pi4k2a, Ppard, Prune2, Rock2, Rusc2, Sdc4, Slc7a11, Wdr26, Cpeb1, Igf1r, Spen, Ubr5, Dab2ip, Gramd1b, Lpin1, Prdx1, Rap1b, Gadd45b, Atf3, Nr4a3, Bhlhe40, Txnip, Mcl1, Zfp36, Bdnf,*** *Sgk1, Crem, Capn2, Jak2, Mapk6, Ddx52, Klhl15, Mical2, Atp6ap2, Dram2, Asb5, Cul3, Fbxo30, H3f3b, Me1, Rab21, Tead4, Ypel5, Lonp2, Myl2, Notch2, Park7, Plcd1, Kras, Qsox1, Stab2, Yes1, Zfp281* |
| Nr2c2_extended (55g)  (IER) | Nr2c2 | *9930021J03Rik, Chd7, Glul, Lpgat1, Lrp5, Nr2c2, Rab2a, Rbm33, Satb1, Scamp1, Tab2, Taf4b, Top2b, Ugcg, Yes1, Nr2c1, Slc25a34, Ccnk, Cdc14b, Cbfb, Nr4a3, Mrpl45, Ypel5, Ccnl1, Cstf3, Dab2ip, Dnajc13, Mtx1, Piwil2, Bcl2l1, Ccny, Fgfr2, Fkbp5, Hspd1, Lgr4, Lrig2, Osbpl2, Pcid2, Pcnx, Smg6, Wdr43, Zbtb1, Actr3, Ap1b1, Fbxw4, Fos, Klf9, Macrod2, Mid1, Phc2, Plbd1, Plekha1, Por, Sel1l3, Tpm3* |
| Nr4a3 (16g)  (IER) | Nr4a3 | *Fermt2, Junb, Pank1, Pfkfb1, Slc25a28, Sorbs1, Txnrd1, Bsdc1, Gm13889, Klf9, Fos, Rab21, Slc4a4, Tfdp2, Tmem140* |
| Fos (91g)  (IER) | Fos | *Aff1, Anxa7, Arhgap42, Arhgef12, Asb5, Atp13a3, Atp2a2, Bdnf, Bhlhe40, Bmpr1b, Chmp4b, Cpeb1, Ddi2, Flnc, Gab2, Gadd45g, Glis3, Hk2, Hspb1, Junb, Mafk, Map2k3, Map4k5, Me1, Midn, Mt2, Nuak1, Pim1, Ppp2ca, Prkg1, R3hdm2, Rapgef6, Ripk1, Rnf10, Rnf2, Rock2, Runx1, Sat1, Sdc4, Setd7, Slc10a6, Slc38a2, Smpx, Tbca, Tfdp2, Tnfaip2, Trib1, Txnrd1, Ubap1, Wdr1, Wdr26, Zfp36, Zfp655, Hes1, Osbp, Pi4k2a, Slc19a2, Abca5, Cbl, Clpx, Gls, Herpud2, Igf1r, Khnyn, Mt1, Myh4, Pcnx, Prkab2, Purg, Rusc2, Slc11a2, Slc41a1, Slc7a11, Smad1, Zfand5, Zfp395, 2900026A02Rik, Ampd3, Arid5a, Cast, Gna13, Hccs, Lrrc58, Mcl1, Nr2c2, Polr3a, Rap1b, Rrad, Zfp281, Pde4b* |
| Hes1 (10g)  (IER) | Hes1 | *Bhlhe40, Ccdc6, Hes1, Atp2a2, Fos, Gadd45b, Gna13, Macrod1, Mt2, Mynn* |
| Cebpd_extended (77g)  (IER) | Cebpd | *Arid5a, Atp13a3, BC005537, Btbd9, Cebpb, Ciapin1, Clu, Eif5, Gab2, H3f3b, Hspa5, Ivns1abp, Junb, Kdm6b, Khnyn, Mafk, Map2k3, Midn, Nfil3, Nr4a3, Rab5a, Sdc4, Slc10a6, Slc25a25, Sorbs1, Spag9, Srsf7, Synj2, Tiprl, Tmem140, Zfp36, Zfp516, Adarb1, Aldh1a1, Bhlhe40, Fzd4, Hspg2, Klf9, Mcl1, Pde4b, Sat1, Smad1, Smad3, Smad5, Vps13a, Aff1, Ampd3, Atf4, B4galt1, Bcl2l11, Btg2, Cab39, Cblb, Cdc14a, Fam98a, Fbxl20, Fkbp5, Gabarapl1, Kank1, Lrrc58, Mid1, Mrpl48, P2ry2, Ptpn3, Qsox1, Rhbdf2, Riok2, Runx1, Rusc2, Sbno2, Sdad1, Slc41a1, Smad4, Tcp11l2, Ube2o, Zfp281* |

Note: The bold font indicates DEGs that were up-regulated in type IIa/IIx_2 myonuclei compared with type IIa/IIx_2 myonuclei.

**Table S19.** The number of muscle stem cells (MuSCs) in each group.

|  | NC_SOL | HS0_SOL | HS8_SOL | NC_Gas | HS0_Gas | HS8_Gas | NC_TA | HS0_TA | HS8_TA | Total |
| --- | --- | --- | --- | --- | --- | --- | --- | --- | --- | --- |
| Quiescent MuSCs | 556 | 483 | 350 | 241 | 137 | 325 | 500 | 294 | 607 | 3493 |
| Activated MuSCs | 48 | 70 | 38 | 1 | 5 | 15 | 28 | 11 | 49 | 265 |
| Myoblasts | 5 | 5 | 3 | 2 | 2 | 3 | 8 | 1 | 7 | 36 |
| Proliferative MuSCs | 3 | 1 | 5 | 2 | 1 | 5 | 4 | 2 | 1 | 24 |
| Total | 612 | 559 | 396 | 246 | 145 | 348 | 540 | 308 | 664 | 3818 |

**Table S20.** Driver genes in differentiation from quiescent to activated MuSCs.

| Tissue | Cluster | State | Genes |
| --- | --- | --- | --- |
| SOL | Cluster 1 | Cell fate 2 | *Gm51425, Gm12966, Gm26549, Med30, Ubn2, Zdhhc1, Dst, Cdk11b, Gm47283, Ccnyl1, Pisd, Rhoc, Bmi1, 4930599N23Rik, Pld3, Acot8, Mospd2, H19, Tnnc2, Csrp3, Tnni2, Lpl, Nrap, Loxl3, Rabggta, Gm7832, Fgf10, Wdr54, Hspb6, Pja1, Ryr1, Tnnt3, Plin4, Cystm1, Pdha1, Tpm3, Supt7l, Dusp12, Cped1, Tpm1, Myl1, Tnnt1, Acadvl, Ckm, Acta1, Atp2a1, Tpm2, Nexn, Pdlim3, Stim1, Mettl15, Add1, Rnf145, Map2k3, Cdc42ep5, Ldlr, Gm26691, Gm16675, Gm49620, Ttn, Tnnc1, Dcn, Mb, Myl3, Sorbs1, Acaa2, Kif1b, Map4, Asb2, D2hgdh, Dgat1* |
|  | Cluster 2 | Cell fate 1 | *Rpl18, Gm15500, Fam98b, Gm13436, Gpx3, Gas1, Vcam1, Slc25a30, Skp2, Gstcd, Mettl25, Dennd11, Hmgxb3, Adamts15, Snx2, Csad, Tia1, Gpm6b, 2700062C07Rik, Rbmxl1, 9230114K14Rik, Tspyl4, Gm9800, Dars2, Jrkl, Acp2, Mrto4* |
|  | Cluster 3 | Root | *Xrcc6, Drg2, Borcs7, Atoh8, Pip4k2c, Obsl1, Zfp354c, Trabd, Sharpin, Hdac2, Vps13c, Dhx40, Heatr5b, Ncoa7, Usp40, Supt3, Thbs3, Slc35e2, Gm22003, Ifit1, Cd109, Tmem242, Nid2, Agap2, Abt1, Ephb6, Psme2, BB365896, Tor1a, Pir, Dpp7, Guca1b, Lbh, Cntn5* |
| Gas | Cluster 1 | Cell fate 1 | *Urb2, Slc2a8, Arhgef19, Itpr3, Tmem223, Cwc22, Rpl7l1, Cep170, Iars, Nat10, Hycc1, D130007C19Rik, Slu7, Erp29, Qsox2, Nckap5, Jph1, Trdn, Slc2a4, Cep85, Vipr2, Mtln, Mitf* |
|  | Cluster 2 | Cell fate 2 | *D330041H03Rik, Cep57l1, Gm42826, Mideas, Pkn2, Slc2a3, Rab11b, Gm19810, Gm10288, Mettl16, Ube2n, Gm28876, Dbnl, Ints1, Dmc1, Dr1, 1700028E10Rik, Zdhhc21, Creb3, Surf1, Rpsa, Gm46546, Rps21, Rps15, Dbt, Mier1, Dhcr24, Vgll3, Medag, Vcam1, Psmg2, Rcn1, Syngap1, Rpl11, Gpx3, Rpl14−ps1, Rps27rt, Rpl13, Efna5, Man2c1os, Serpini1, Mrtfb, Ercc8, Prkcsh, Med31, Acp2, Mageh1* |
|  | Cluster 3 | Root | *Mrto4, Zdhhc4, Nacc1, Fibp, Gm5124, Scrn1, Skp2, Eif2b4, Cyp39a1, Kctd3, Tmem41b, Hsbp1, Map3k7, Jmjd8, S1pr3, Haus8, Gm15246, Cyrib, 5031434O11Rik, Lurap1l, Hirip3, Zfp36, 6720427I07Rik, Ntn5, Brdt, Nebl, Erap1, Olfml3, Ybx1, Mob1a, Soga1, Mrps10, Nup214, Zer1, Gm13611, Zfp335* |

**Table S21.** The coefficient of variation (CV) of proportion of CCIs number in each group.

|  | Myonuclei | MuSCs | FAPs | SMCs | ECs | Adipocytes | Immune cells |
| --- | --- | --- | --- | --- | --- | --- | --- |
| NC_SOL | 22.45% | 30.61% | 23.62% | 32.65% | 27.99% | 23.03% | 25.07% |
| HS0_SOL | 16.79% | 31.39% | 27.74% | 31.02% | 28.83% | 16.42% | 25.18% |
| HS8_SOL | 12.04% | 29.01% | 28.40% | 36.42% | 34.26% | 17.28% | 21.30% |
| Mean | 17.09% | 30.34% | 26.58% | 33.36% | 30.36% | 18.91% | 23.85% |
| STDEV | 0.0521 | 0.0121 | 0.0259 | 0.0277 | 0.0340 | 0.0359 | 0.0221 |
| CV | 0.3050 | 0.0399 | 0.0975 | 0.0830 | 0.1121 | 0.1900 | 0.0928 |
| NC_Gas | 1.04% | 28.82% | 26.04% | 33.33% | 30.21% | 26.04% | 29.86% |
| HS0_Gas | 12.00% | 32.80% | 29.60% | 32.80% | 32.00% | 20.80% | 30.40% |
| HS8_Gas | 12.46% | 31.49% | 27.34% | 37.02% | 30.10% | 21.45% | 19.72% |
| Mean | 8.50% | 31.04% | 27.66% | 34.39% | 30.77% | 22.76% | 26.66% |
| STDEV | 0.0646 | 0.0203 | 0.0180 | 0.0230 | 0.0107 | 0.0286 | 0.0601 |
| CV | 0.7604 | 0.0654 | 0.0651 | 0.0669 | 0.0346 | 0.1255 | 0.2256 |
| NC_TA | 0.46% | 36.99% | 29.22% | 29.22% | 35.62% | 21.92% | 26.94% |
| HS0_TA | 17.14% | 29.05% | 30.95% | 35.24% | 32.86% | 0 | 34.29% |
| HS8_TA | 9.79% | 34.89% | 29.79% | 39.15% | 38.72% | 0 | 25.11% |
| Mean | 9.13% | 33.64% | 29.99% | 34.54% | 35.73% | 7.31% | 28.78% |
| STDEV | 0.0836 | 0.0411 | 0.0088 | 0.0500 | 0.0293 | 0.1265 | 0.0486 |
| CV | 0.9161 | 0.1223 | 0.0294 | 0.1448 | 0.0821 | 1.7321 | 0.1688 |

**References of Table S1 and Table S2**

1 Han, L. *et al.* Cell transcriptomic atlas of the non-human primate Macaca fascicularis. *Nature* **604**, 723-731, doi:10.1038/s41586-022-04587-3 (2022).

2 Petrany, M. J. *et al.* Single-nucleus RNA-seq identifies transcriptional heterogeneity in multinucleated skeletal myofibers. *Nature communications* **11**, 6374, doi:10.1038/s41467-020-20063-w (2020).

3 Chemello, F. *et al.* Degenerative and regenerative pathways underlying Duchenne muscular dystrophy revealed by single-nucleus RNA sequencing. *Proceedings of the National Academy of Sciences of the United States of America* **117**, 29691-29701, doi:10.1073/pnas.2018391117 (2020).

4 Min, S.-K. *et al.* Quantifying human impact on the 2018 summer longest heat wave in South Korea. *Bulletin of the American Meteorological Society* **101**, S103–S108 (2020).

5 Dos Santos, M. *et al.* Opposing gene regulatory programs governing myofiber development and maturation revealed at single nucleus resolution. *Nature communications* **14**, 4333, doi:10.1038/s41467-023-40073-8 (2023).

6 Rubenstein, A. B. *et al.* Single-cell transcriptional profiles in human skeletal muscle. *Scientific reports* **10**, 229, doi:10.1038/s41598-019-57110-6 (2020).

7 Dell'Orso, S. *et al.* Single cell analysis of adult mouse skeletal muscle stem cells in homeostatic and regenerative conditions. *Development (Cambridge, England)* **146**, doi:10.1242/dev.174177 (2019).

8 Soule, T. G. *et al.* A protocol for single nucleus RNA-seq from frozen skeletal muscle. *Life science alliance* **6**, doi:10.26508/lsa.202201806 (2023).

9 Farup, J. *et al.* Human skeletal muscle CD90(+) fibro-adipogenic progenitors are associated with muscle degeneration in type 2 diabetic patients. *Cell metabolism* **33**, 2201-2214.e2211, doi:10.1016/j.cmet.2021.10.001 (2021).

10 Orchard, P. *et al.* Human and rat skeletal muscle single-nuclei multi-omic integrative analyses nominate causal cell types, regulatory elements, and SNPs for complex traits. *Genome research* **31**, 2258-2275, doi:10.1101/gr.268482.120 (2021).

11 Kimmel, J. C., Yi, N., Roy, M., Hendrickson, D. G. & Kelley, D. R. Differentiation reveals latent features of aging and an energy barrier in murine myogenesis. *Cell reports* **35**, 109046, doi:10.1016/j.celrep.2021.109046 (2021).

12 Potluri, T. *et al.* An estrogen-sensitive fibroblast population drives abdominal muscle fibrosis in an inguinal hernia mouse model. *JCI insight* **7**, doi:10.1172/jci.insight.152011 (2022).

13 Wang, L. *et al.* Single-nucleus and bulk RNA sequencing reveal cellular and transcriptional mechanisms underlying lipid dynamics in high marbled pork. *NPJ science of food* **7**, 23, doi:10.1038/s41538-023-00203-4 (2023).

14 Sárvári, A. K. *et al.* Plasticity of Epididymal Adipose Tissue in Response to Diet-Induced Obesity at Single-Nucleus Resolution. *Cell metabolism* **33**, 437-453.e435, doi:10.1016/j.cmet.2020.12.004 (2021).

15 Lin, H. *et al.* Reprogramming of cis-regulatory networks during skeletal muscle atrophy in male mice. *Nature communications* **14**, 6581, doi:10.1038/s41467-023-42313-3 (2023).

16 Dos Santos, M. *et al.* Single-nucleus RNA-seq and FISH identify coordinated transcriptional activity in mammalian myofibers. *Nature communications* **11**, 5102, doi:10.1038/s41467-020-18789-8 (2020).

17 Williams, K., Yokomori, K. & Mortazavi, A. Heterogeneous Skeletal Muscle Cell and Nucleus Populations Identified by Single-Cell and Single-Nucleus Resolution Transcriptome Assays. *Frontiers in genetics* **13**, 835099, doi:10.3389/fgene.2022.835099 (2022).

18 Beauchamp, J. R. *et al.* Expression of CD34 and Myf5 defines the majority of quiescent adult skeletal muscle satellite cells. *The Journal of cell biology* **151**, 1221-1234, doi:10.1083/jcb.151.6.1221 (2000).

19 Shea, K. L. *et al.* Sprouty1 regulates reversible quiescence of a self-renewing adult muscle stem cell pool during regeneration. *Cell stem cell* **6**, 117-129, doi:10.1016/j.stem.2009.12.015 (2010).

20 Barruet, E. *et al.* Functionally heterogeneous human satellite cells identified by single cell RNA sequencing. *eLife* **9**, doi:10.7554/eLife.51576 (2020).

21 Wang, J. *et al.* Myoblast deactivation within engineered human skeletal muscle creates a transcriptionally heterogeneous population of quiescent satellite-like cells. *Biomaterials* **284**, 121508, doi:10.1016/j.biomaterials.2022.121508 (2022).

22 De Micheli, A. J. *et al.* Single-Cell Analysis of the Muscle Stem Cell Hierarchy Identifies Heterotypic Communication Signals Involved in Skeletal Muscle Regeneration. *Cell reports* **30**, 3583-3595.e3585, doi:10.1016/j.celrep.2020.02.067 (2020).

23 Yartseva, V. *et al.* Heterogeneity of Satellite Cells Implicates DELTA1/NOTCH2 Signaling in Self-Renewal. *Cell reports* **30**, 1491-1503.e1496, doi:10.1016/j.celrep.2019.12.100 (2020).

24 Zhang, C. *et al.* Age-related decline of interferon-gamma responses in macrophage impairs satellite cell proliferation and regeneration. *Journal of cachexia, sarcopenia and muscle* **11**, 1291-1305, doi:10.1002/jcsm.12584 (2020).

25 Wu, Y. E., Pan, L., Zuo, Y., Li, X. & Hong, W. Detecting Activated Cell Populations Using Single-Cell RNA-Seq. *Neuron* **96**, 313-329.e316, doi:10.1016/j.neuron.2017.09.026 (2017).

26 Tullai, J. W. *et al.* Immediate-early and delayed primary response genes are distinct in function and genomic architecture. *The Journal of biological chemistry* **282**, 23981-23995, doi:10.1074/jbc.M702044200 (2007).
