# Supplementary material for: Single‐Nucleus Transcriptome Reveals Cellular Heterogeneity and Transcriptional Response to Heat Stress in Skeletal Muscle
Source: J Cachexia Sarcopenia Muscle. 2026 Feb 12;17(1):e70217. doi: 10.1002/jcsm.70217 (PMC12895210; doi:10.1002/jcsm.70217)
Supplement: Supplementary file 4 — Data S4: Supporting Information. [file JCSM-17-e70217-s008.docx]

**Supplemental figure legend**

**Fig S1. Representative photomicrographs of soleus (SOL) (a) and gastrocnemius (Gas) (b) stained with hematoxylin and eosin (HE).** The black arrows indicated myolysis (middle panel). Cross-sectional area (CSA) of TA based on HE staining (right panel). The values of “*n*” represented the total number of myofibers for cross sectional area measurements. Myofibers were derived from 3 biological replicates, each with 2 technical replicates (*n* = 6). The CSA of each myofiber was shown in Table S6-7.

**Fig S2. Cell annotation in the atlas of skeletal muscles.** (a) The number of genes and unique molecular identifier (UMI) counts per high quality nucleus. (b) Individual gene uniform manifold approximation and projection (UMAP) plots indicating the expression levels and distribution of known marker genes of myonuclei, fibroadipogenic progenitors (FAPs), endothelial cells (ECs), smooth muscle cells (SMCs), muscle stem cells (MuSCs), immune cells and adipocytes. (c-d) The identification of cell-type-specific modules using high-dimensional weighted gene co-expression network analysis (hdWGCNA). (c) UMAP plot showing the expression distribution of hub genes for each module across the seven cell types. (d) The average expression of module-specific hub genes in different cell types. (e) The function description of hub genes in each cell type.

**Fig S3. Differential gene expression of six cell types in each skeletal muscle (SOL, Gas, and TA).** Upset plots represent overlapping differentially expressed genes (DEGs) during (a) heat treatment (NC vs. HS0 group) and (b) 8 hours of recovery from heat (HS8 vs. HS0 group).

**Fig S4. Immediate early genes (IEGs) and transcriptional factor (TF) regulon. (a) The distribution of differentially expressed** **IEGs in muscle stem cells (MuSCs),** **fibroadipogenic progenitors (FAPs), endothelial cells (ECs), smooth muscle cells (SMCs), and immune cells. (b) Motifs in the promoter regions of DEGs identified from Gas and TA binding by Mef2c.**

**Fig S5. A single-nuclei atlas for the mice myonuclei.** (a) Individual gene uniform manifold approximation and projection (UMAP) plots indicating the expression levels and distribution of known marker genes for type I myonuclei, type IIa/IIx myonuclei, type IIb myonuclei, myotendinous junction (MTJ), and neuromuscular junction (NMJ). (b) The distribution of myonuclei in each group. (c) Functional analysis of the high variable genes (HVGs) in type IIa and IIb myonulei subtypes. **(d)** **The 27 up-regulated immediate early genes in type IIa/IIx_2 myonuclei compared to type IIa/IIx_2 myonuclei.**

**Fig S6. Immunohistochemistry or immunofluorescence of type IIa/IIx_2 and IIb_2 myofibers.** (a) The mRNA and protein expression of *Stat3*, a type IIa/IIx_2 myonuclei-specific marker, in SOL. (b) The distribution of type IIa/IIx_2 myonuclei in SOL. (c) The proportion of type IIa/IIx_2 myofiber/myonuclei identified by immunofluorescence, immunohistochemistry, and snRNA-seq. (d) The mRNA and protein expression of *Hsp70*, a type IIb_2 myonuclei-specific marker, in Gas. (e) The distribution of type IIb_2 myonuclei in Gas. (f) The proportion of type IIb_2 myofiber/myonuclei identified by immunofluorescence and snRNA-seq. In the Fig S5, immunohistochemistry and immunofluorescence assays were based on two biological replicates with 5 to 11 technical replicates.

**Fig S7. Muscle stem cells (MuSCs) participate in tissue repair after heat treatment.** (a) The biological function of hypervariable genes (HVGs) in each subtype of MuSCs. (b) The velocity and cell entropy of MuSCs. (c) Differentiation trajectoriey (left panel) and driver genes (middle panel) from quiescent to activated MuSCs in Gas. Activated MuSCs were predominantly localized to State 5 (Cell fate 1) and State 6 (Cell fate 2). GO terms and KEGG pathway analyses of the two states of activated MuSCs (right panel). (d) GO terms and KEGG pathway analyses of high variable genes identified in activated MuSCs of SOL (upper panel) and Gas (lower panel) of HS8 group.

**Fig S8. The number of activated MuSCs in TA.** (a) UMAP projection of 3,278 cells from the tibialis anterior (TA) (left panel). Dot plot displays expression levels of marker genes across TA cell types (right panel; Santos et al.). (b) Cell composition of the TA (Santos et al.). (c) UMAP visualization of 98 MuSC subtypes in the TA (left panel). Dot plot shows marker gene expression patterns among MuSC subtypes (right panel; Santos et al.). (d) Proportion of activated MuSCs relative to total MuSCs/all cells in TA (left panel: data from Santos et al.; right panel: present study).

**Fig S9. The expression of genes, GO terms and KEGG pathways related to immune response in SOL (a) and Gas (c) HS0 and HS8 groups.**
