# Supplementary figures and images for: Single‐Nucleus Transcriptome Reveals Cellular Heterogeneity and Transcriptional Response to Heat Stress in Skeletal Muscle
Source: J Cachexia Sarcopenia Muscle. 2026 Feb 12;17(1):e70217. doi: 10.1002/jcsm.70217 (PMC12895210; doi:10.1002/jcsm.70217)

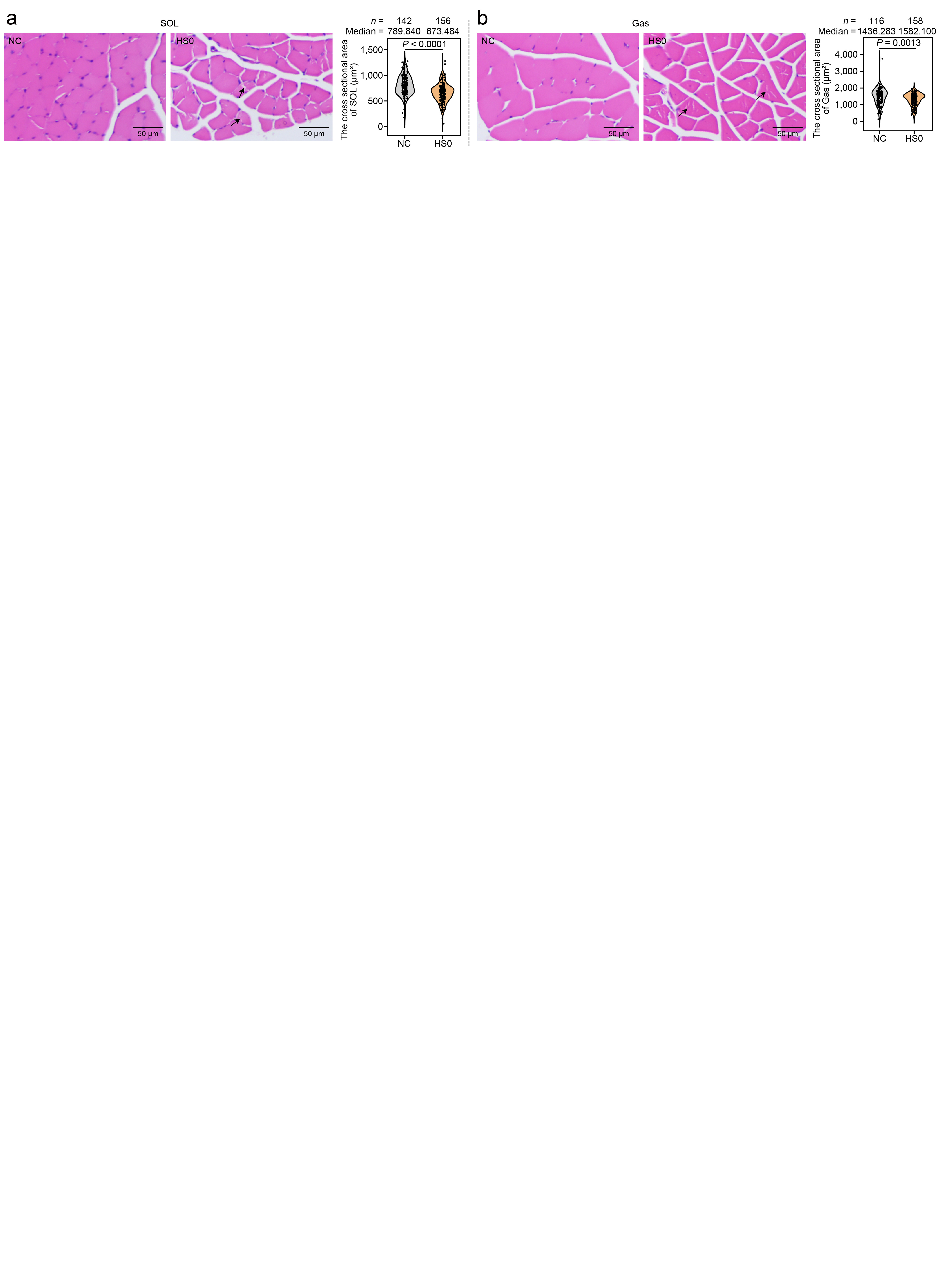

Supplement: Supplementary file 5 — Figure S1: Representative photomicrographs of (a) soleus (SOL) and (b) gastrocnemius (Gas) stained with haematoxylin and eosin (HE). The black arrows indicated myolysis (middle panel). Cross‐sectional area (CSA) of TA based on HE staining (right panel). The values of ‘n’ represented the total number of myofibres for cross‐sectional area measurements. Myofibres were derived from three biological replicates, each with two technical replicates (n = 6). The CSA of each myofibre was shown in Tables S6 and S7. [file JCSM-17-e70217-s011.jpg]

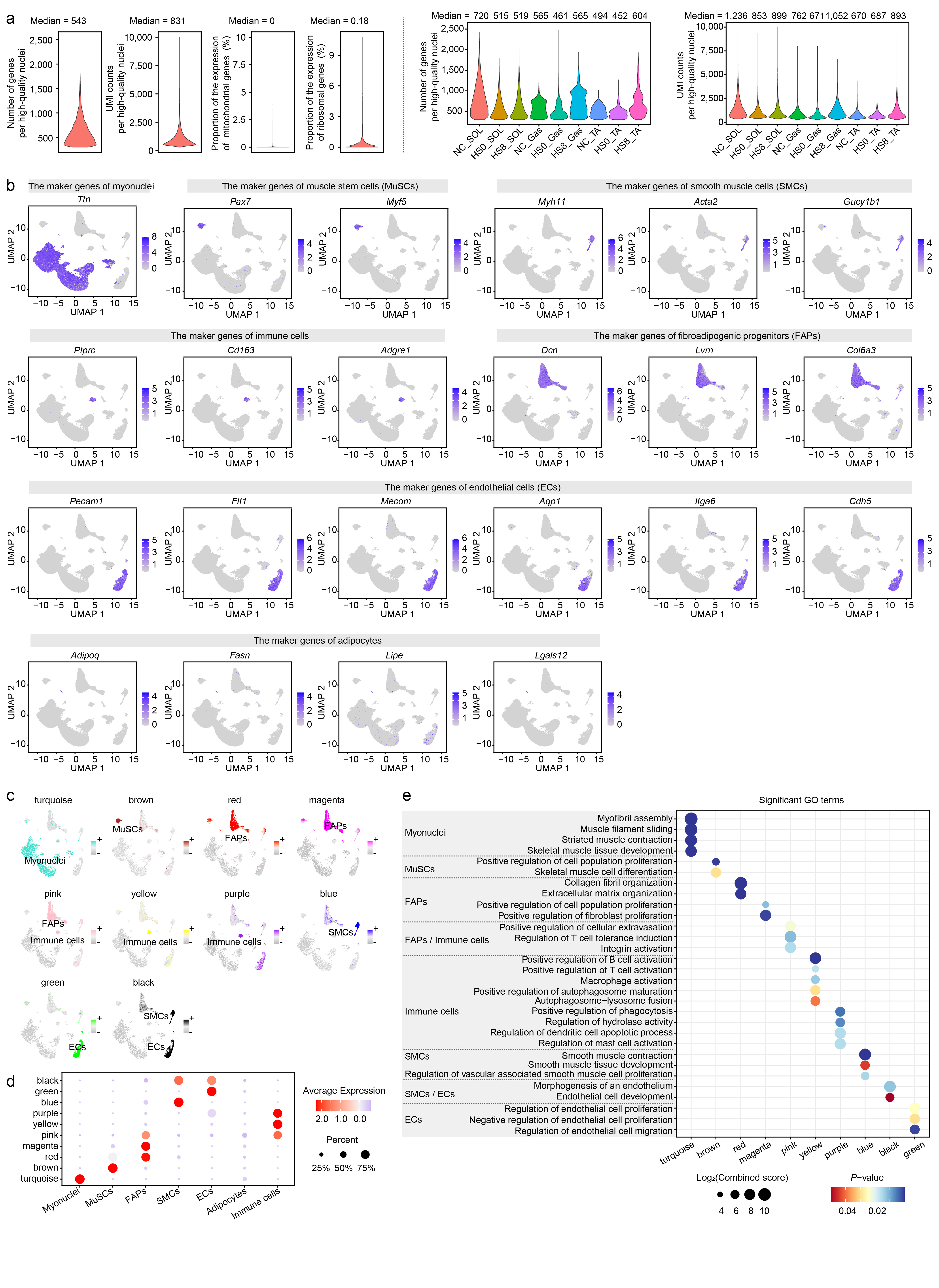

Supplement: Supplementary file 6 — Figure S2: Cell annotation in the atlas of skeletal muscles. (a) The number of genes and unique molecular identifier (UMI) counts per high‐quality nucleus. (b) Individual gene uniform manifold approximation and projection (UMAP) plots indicating the expression levels and distribution of known marker genes of myonuclei, fibroadipogenic progenitors (FAPs), endothelial cells (ECs), smooth muscle cells (SMCs), muscle stem cells (MuSCs), immune cells and adipocytes. (c,d) The identification of cell type–specific modules using high‐dimensional weighted gene coexpression network analysis (hdWGCNA). (c) UMAP plot showing the expression distribution of hub genes for each module across the seven cell types. (d) The average expression of module‐specific hub genes in different cell types. (e) The function description of hub genes in each cell type. [file JCSM-17-e70217-s009.jpg]

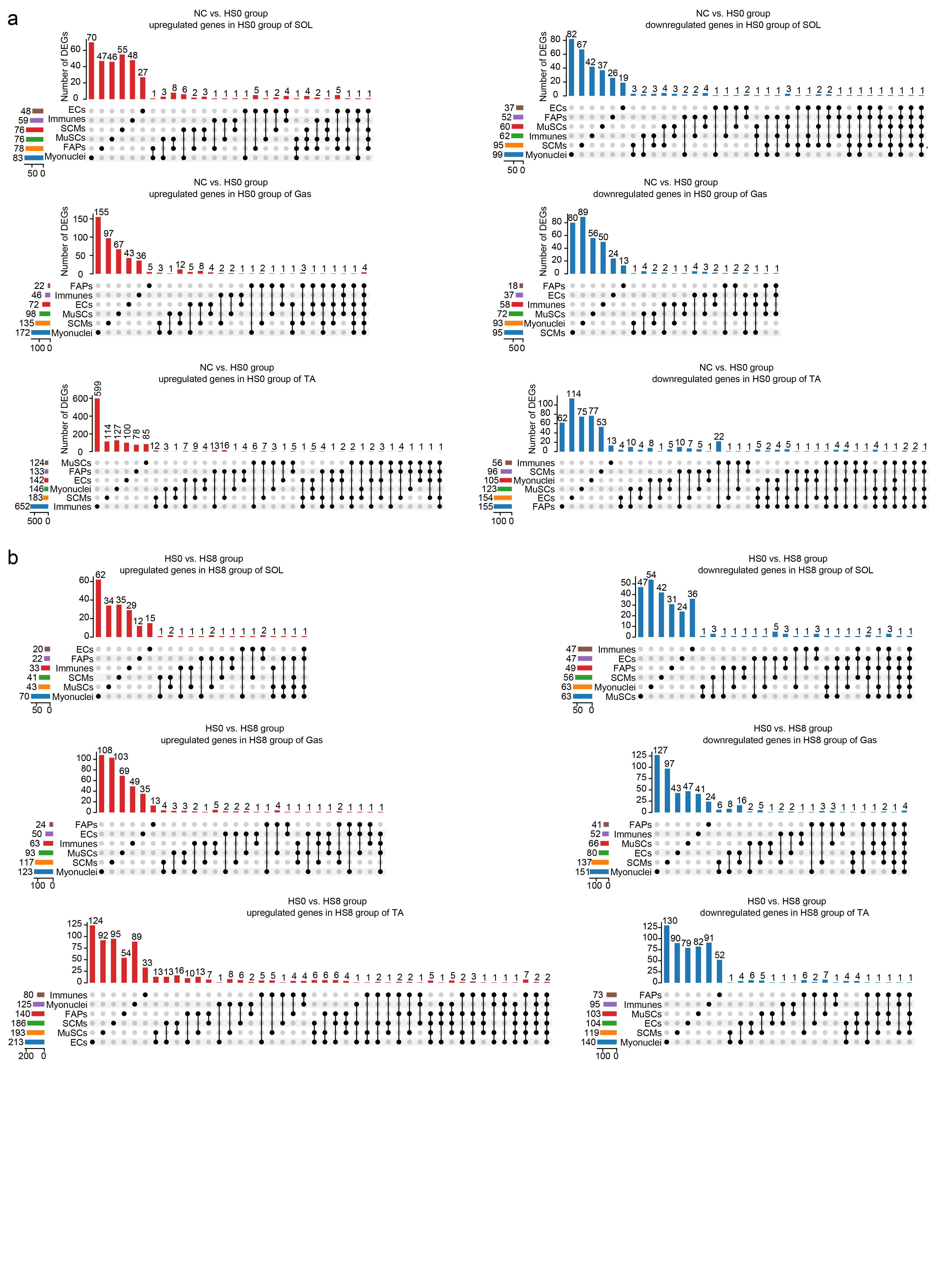

Supplement: Supplementary file 7 — Figure S3: Differential gene expression of six cell types in each skeletal muscle (SOL, Gas and TA). Upset plots represent overlapping differentially expressed genes (DEGs) during (a) heat treatment (NC vs. HS0 group) and (b) 8 h of recovery from heat (HS8 vs. HS0 group). [file JCSM-17-e70217-s013.jpg]

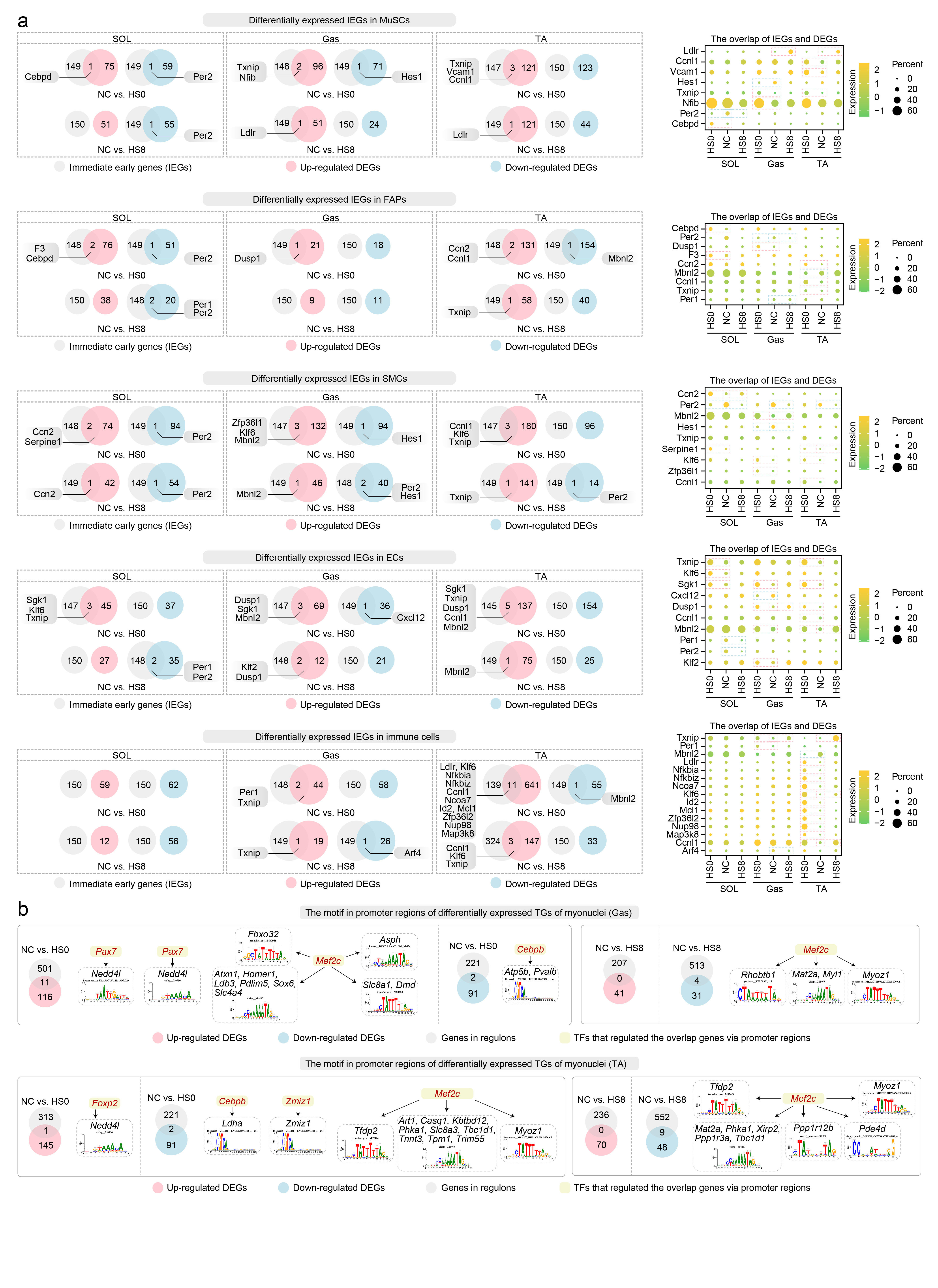

Supplement: Supplementary file 8 — Figure S4: Immediate early genes (IEGs) and transcriptional factor (TF) regulon. (a) The distribution of differentially expressed IEGs in muscle stem cells (MuSCs), fibroadipogenic progenitors (FAPs), endothelial cells (ECs), smooth muscle cells (SMCs) and immune cells. (b) Motifs in the promoter regions of DEGs identified from Gas and TA binding by Mef2c. [file JCSM-17-e70217-s006.jpg]

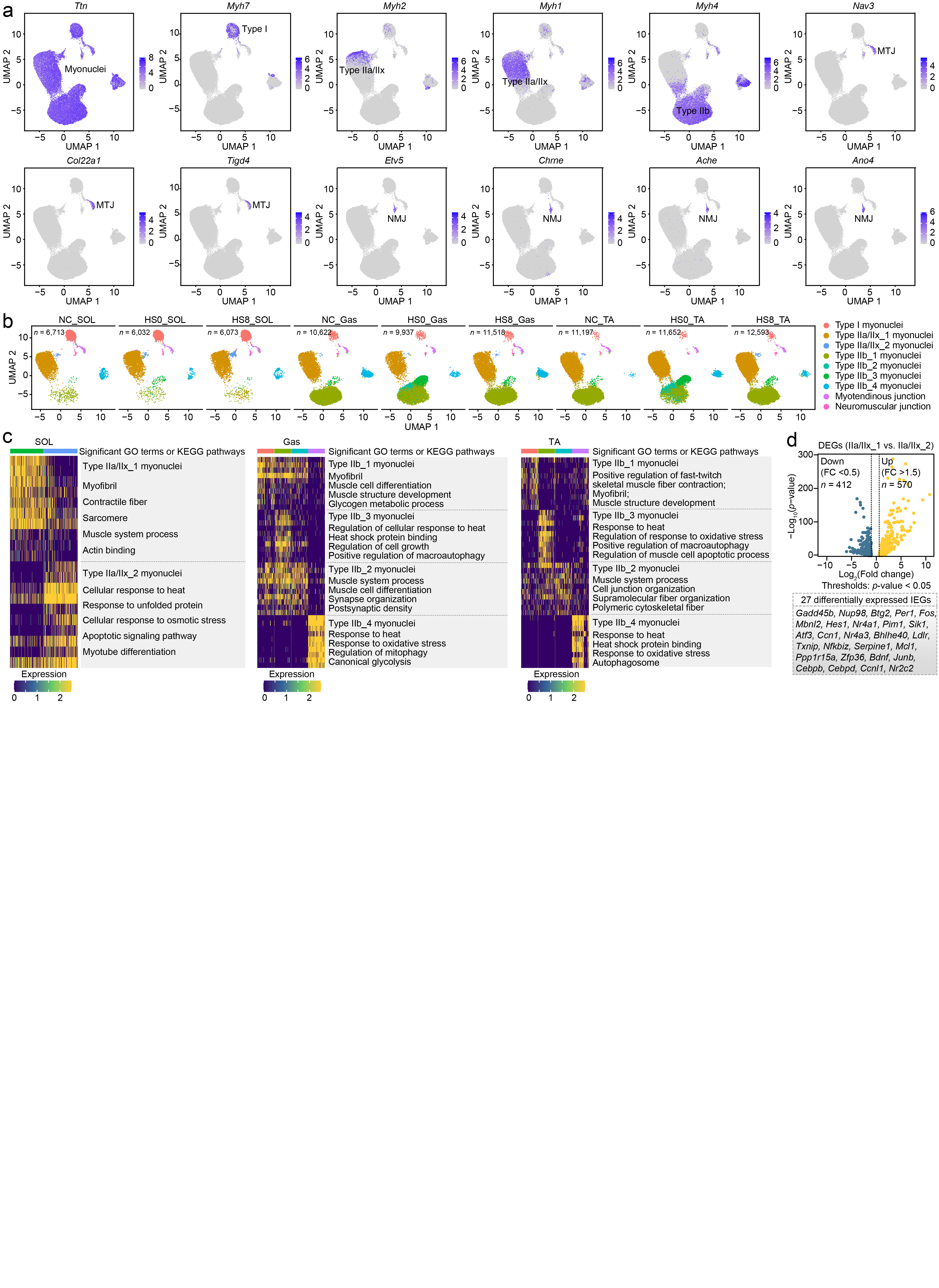

Supplement: Supplementary file 9 — Figure S5: A single‐nucleus atlas for the mice myonuclei. (a) Individual gene uniform manifold approximation and projection (UMAP) plots indicating the expression levels and distribution of known marker genes for type I myonuclei, type IIa/IIx myonuclei, type IIb myonuclei, myotendinous junction (MTJ) and neuromuscular junction (NMJ). (b) The distribution of myonuclei in each group. (c) Functional analysis of the high variable genes (HVGs) in type IIa and IIb myonulei subtypes. (d) The 27 upregulated immediate early genes in type IIa/IIx_2 myonuclei compared with type IIa/IIx_2 myonuclei. [file JCSM-17-e70217-s012.jpg]

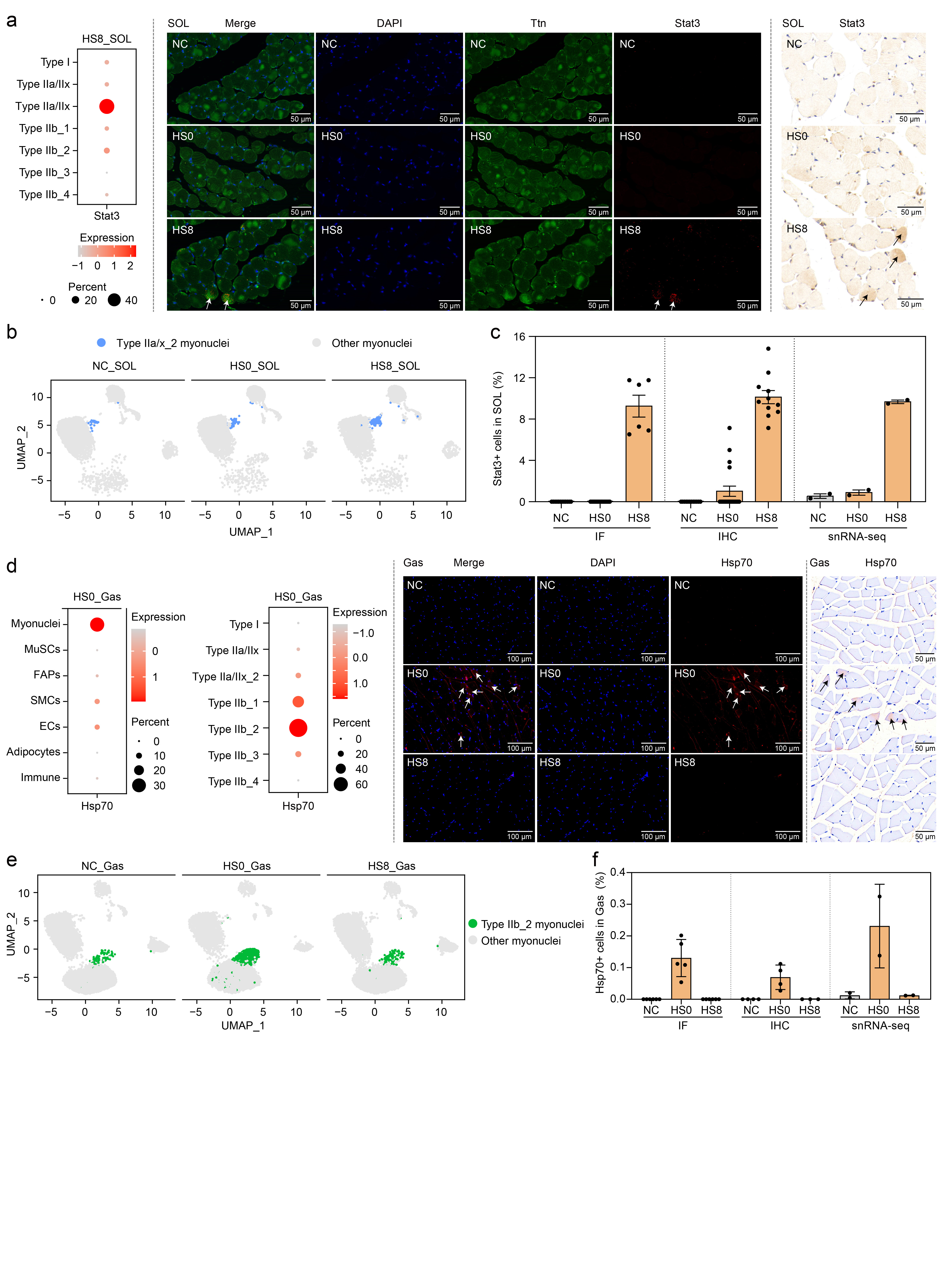

Supplement: Supplementary file 10 — Figure S6: Immunohistochemistry or immunofluorescence of type IIa/IIx_2 and IIb_2 myofibres. (a) The mRNA and protein expression of Stat3, a type IIa/IIx_2 myonuclei‐specific marker, in SOL. (b) The distribution of type IIa/IIx_2 myonuclei in SOL. (c) The proportion of type IIa/IIx_2 myofibre/myonuclei identified by immunofluorescence, immunohistochemistry and snRNA‐seq. (d) The mRNA and protein expression of Hsp70, a type IIb_2 myonuclei‐specific marker, in Gas. (e) The distribution of type IIb_2 myonuclei in Gas. (f) The proportion of type IIb_2 myofibre/myonuclei identified by immunofluorescence and snRNA‐seq. In Figure S5, immunohistochemistry and immunofluorescence assays were based on two biological replicates with 5–11 technical replicates. [file JCSM-17-e70217-s003.jpg]

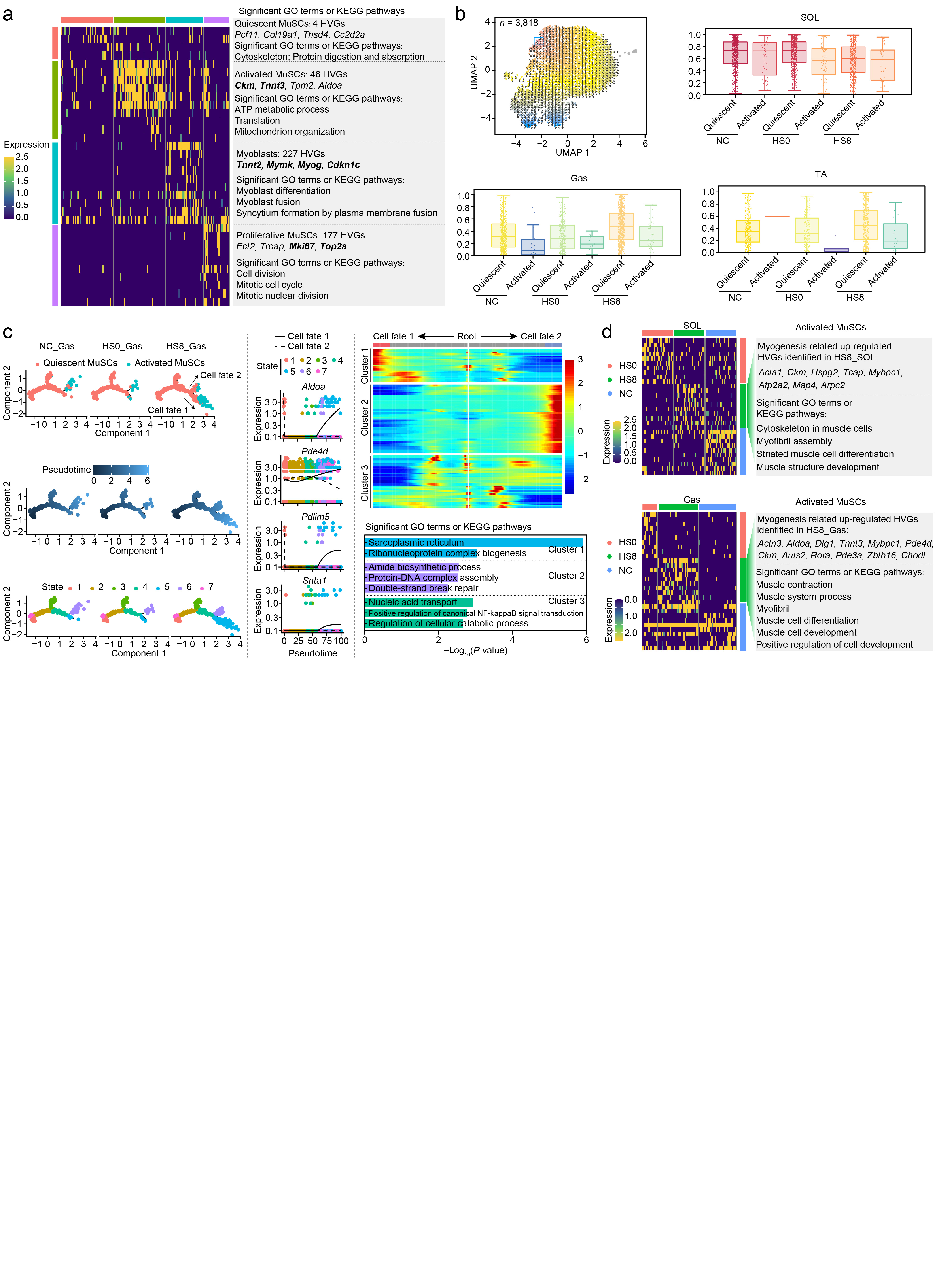

Supplement: Supplementary file 11 — Figure S7: Muscle stem cells (MuSCs) participate in tissue repair after heat treatment. (a) The biological function of hypervariable genes (HVGs) in each subtype of MuSCs. (b) The velocity and cell entropy of MuSCs. (c) Differentiation trajectory (left panel) and driver genes (middle panel) from quiescent to activated MuSCs in Gas. Activated MuSCs were predominantly localised to State 5 (Cell Fate 1) and State 6 (Cell Fate 2). GO terms and KEGG pathway analyses of the two states of activated MuSCs (right panel). (d) GO terms and KEGG pathway analyses of high variable genes identified in activated MuSCs of SOL (upper panel) and Gas (lower panel) of HS8 group. [file JCSM-17-e70217-s010.jpg]

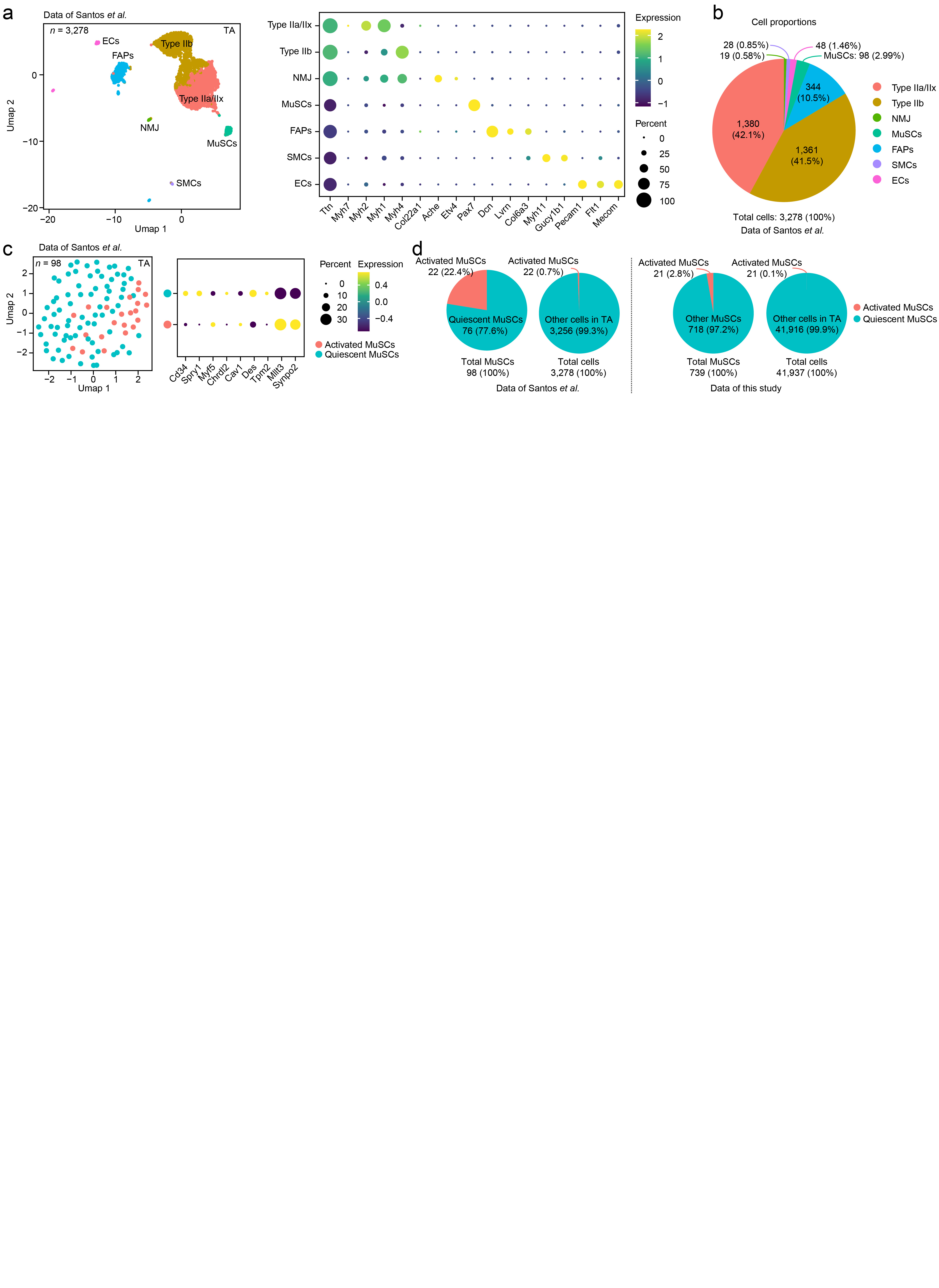

Supplement: Supplementary file 12 — Figure S8: The number of activated MuSCs in TA. (a) UMAP projection of 3278 cells from the tibialis anterior (TA) (left panel). Dot plot displays expression levels of marker genes across TA cell types (right panel; Santos et al.). (b) Cell composition of the TA (Santos et al.). (c) UMAP visualisation of 98 MuSC subtypes in the TA (left panel). Dot plot shows marker gene expression patterns among MuSC subtypes (right panel; Santos et al.). (d) Proportion of activated MuSCs relative to total MuSCs/all cells in TA (left panel: data from Santos et al.; right panel: present study). [file JCSM-17-e70217-s007.jpg]

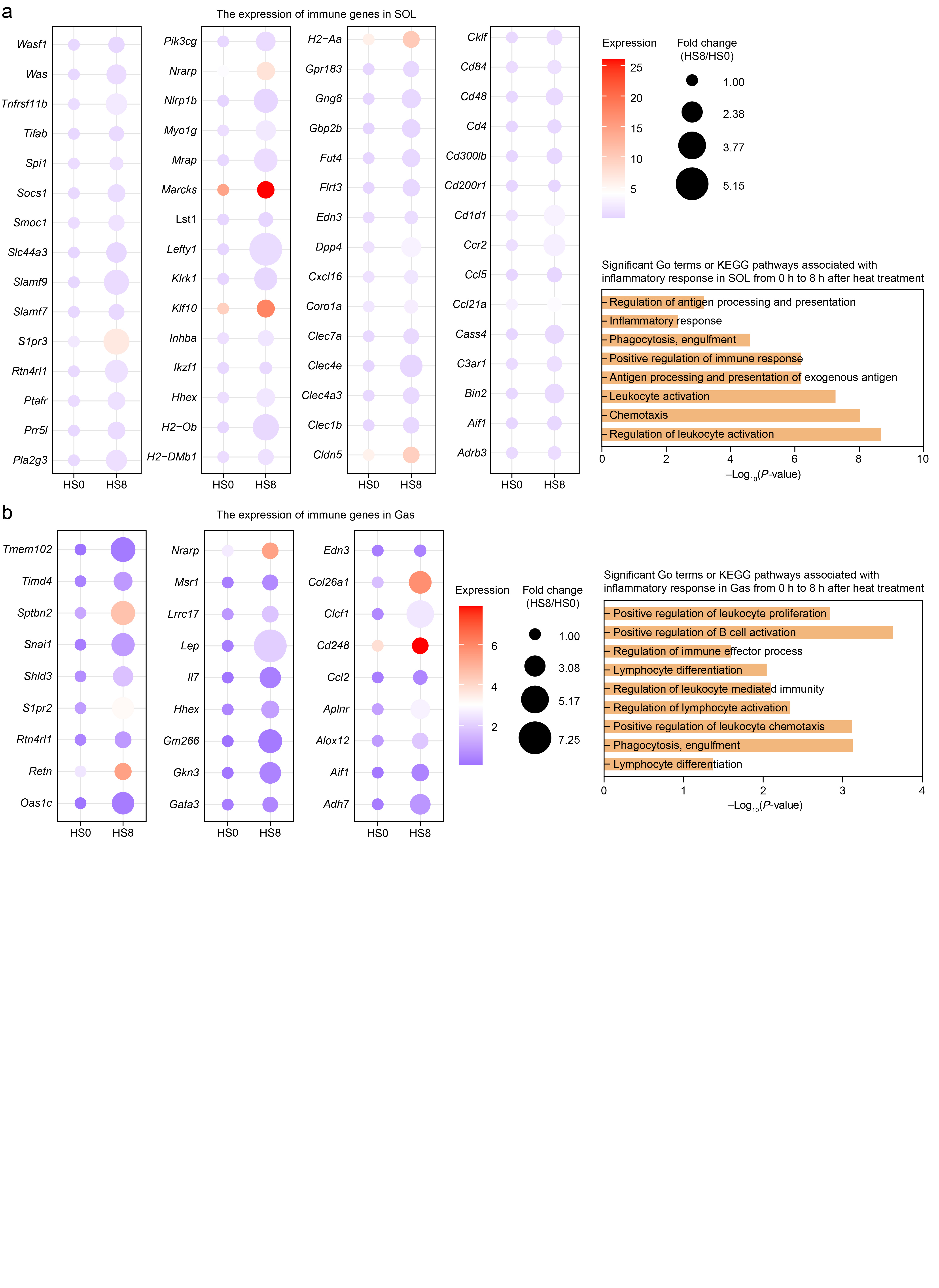

Supplement: Supplementary file 13 — Figure S9: The expression of genes, GO terms and KEGG pathways related to immune response in (a) SOL and (b) Gas HS0 and HS8 groups. [file JCSM-17-e70217-s002.jpg]
